# HCG supplement did not accelerate tunica albuginea remodeling to facilitate penile growth

**DOI:** 10.1038/s41598-023-38888-y

**Published:** 2023-10-02

**Authors:** Tao Li, Yuan Tian, Quliang Zhong, Peng Chen, Junhao Zhang, Guangshi Du, Lei Li, Yiting Jiang, Kehua Jiang

**Affiliations:** 1 Department of Urology, Guizhou Provincial People’s Hospital, 184 Zhongshan East Road, Guiyang, 550002 Guizhou People’s Republic of China; 2 Department of Urology, Affiliated Hospital of Guizhou Medical University, Guiyang, People’s Republic of China; 3 Translational Medicine Research Center of Guizhou Medical University, Guiyang, People’s Republic of China; 4 Gastrointestinal Surgery Center, Sichuan Cancer Hospital and Institute, Sichuan Cancer Center, School of Medicine, University of Electronic Science and Technology of China, Chengdu, People’s Republic of China; 5 Department of Otorhinolaryngology, Affiliated Hospital of Guizhou Medical University, Guiyang, People’s Republic of China

**Keywords:** Diseases, Urology

## Abstract

Penile size is closely concerned and short penis contributes serious sexual dysfunction and tremendous psychological problems to couples. Androgen is essential for penile development and testosterone replacement is recommended to patients with micropenis. We previously proved that inhibiting activity of lysyl oxidase (Anti-lysyl oxidase, Anti-LOX) combined with vacuum erectile device (VED) lengthened penis by remodeling tunica albuginea. We thus explored whether HCG supplement could accelerate tunica albuginea remodeling (induced by Anti-LOX + VED) to promote penile growth. Forty-two SD male rats (4 weeks old) were purchased and divided into 7 groups: control, Anti-LOX, HCG, VED (with a negative aspirated pressure of − 300 mmHg), Anti-LOX + VED, HCG + VED, and Anti-LOX + HCG + VED. After an intervention for 4 weeks, all rats’ penile length, exposed penile length, and erectile function were measured. Serum samples were collected to detect hormone levels and penile corpus cavernosum were harvested for histo-pathological analysis. All intervention groups showed significantly longer penis than controlled rats. Anti-LOX sharply increased penile length and exposed length by 15% and 9% respectively, this lengthening effect was more obvious in Anti-LOX + VED group (26% and 19%, respectively). Although HCG promoted penile length by 8%, this effect was slight for exposed length (3%). Moreover, Anti-LOX + HCG + VED dramatically increased penile length and exposed length by 22% and 18%, respectively, which was similar with that in Anti-LOX + VED (26% and 19%, respectively). HCG dramatically stimulated testosterone and dihydrotestosterone secretions than control group, whether with or without Anti-LOX and VED; while it induced more AR expression than other groups. Finally, all procedures did not improve or deteriorate normal erectile function. Although we verified that Anti-LOX + VED lengthened penis by inducing tunica albuginea remodeling, however, HCG supplement did not synergize with Anti-LOX + VED to accelerate albuginea remodeling to facilitate penile growth.

## Introduction

As an essential male reproductive organ and indicator for sexual development, penis was a symbol of strength and masculinity throughout human history^1–3^. Short penis which refers micropenis (normally formed but stretched length < 2.5 SD of normal median, like < 7.5 cm for adult men) or acquired penile retraction (Peyronie’s disease, post-trauma, post-infection, and post-priapism)^4–9^ has contributed various psychological problems^3,4^ and affected the sexual quality of life, fear of sexual relationship, premature ejaculation (PE), as well as impotence^10,11^. These patients always have strong desire to possess a larger penis to satisfy partners or just improve self-esteem^1,3^, which present considerable challenges for urologists/plastic surgeons^9,12^.

Currently, numerous techniques and methods have been recommended to lengthen or enlarge penis. However, the widely advertised non-invasive techniques like penile extender, penoscrotal rings, and botulinum toxin are limited by insufficient scientific evidence^6^. The invasive phalloplasties like inverted V–Y plasty closure, penile suspensory ligament division, and venous grafing for the corpora cavernosa remain controversial^6^ and experimental^1,5^, considering the lengthening effect is unclear and the surgical method or indication is poorly standard^5,6^. Even though, more than 10 000 males received penile lengthening surgery in US during 1991–1998^1^. Recently, more individuals with a normal penile size also consult to enlarge penis just for aesthetic reasons^1,4^. Although a suitable psychotherapy to convince they have a normal size is the best solution^1,9^, some authors has recommended penile lengthening as an aesthetic plastic surgery rather than just reconstructive operation, considering the huge potential population^4^. Therefore, more explorations to lengthen penis are necessary, except intensive psychosexual counselling.

Penile size during tumescence is determined by tunica albuginea which mainly composed by abundant thick collagen bundles and ample elastic fibers^13,14^. We previously found that inhibiting activity of lysyl oxidase (Anti-lysyl oxidase, Anti-LOX) remodeled tunica albuginea by reducing collagen crosslinking to increase penile length by 10.8% for adult rats, while a vacuum erectile device (VED) force induced collagen realignment to lengthen penis by 8.2%^15^. Moreover, Anti-LOX combined with VED (Anti-LOX + VED) contributed more remarkable albugineas remodeling and lengthened penis by 17.4% for adult rats^15^ and 19.84% for pubertal rats^16^.

Human penile development is closely dependent on androgen, especially during the third periods of late gestation, first 4 years after birth, and puberty^17–19^. Penile growth is relatively slow after birth but reaches a peak from 12 to 16 years, which is coinciding with the spurt of testicular development and testosterone secretion^15,20^. Although remained some controversy, testosterone replacement has been recommended for patients with congenital micropenis^9,12,15,19,21,22^. Considering its crucial role in regulating penile growth, we wondered whether HCG (FDA approved testosterone releasing agent without impairing spermatogenesis^23^) supplement could synergize with Anti-LOX + VED to accelerate tunica albugineas remodeling, and finally promote penile length.

## Material and methods

This study was approved by Animal Ethics Committee of Guizhou Provincial People’s Hospital (No. 2020064), Guiyang, China. All rats were housed under standard guidelines, while all methods were carried out in accordance with relevant regulations and ARRIVE guidelines. Forty-two Sprague–Dawley male rats (4 weeks old, about 130 g) were purchased (Dashuo Experimental Animal, Co Ltd, Chengdu, Sichuan Procince, China) and randomly divided into 7 groups (6 rats for each group) after adaptive feeding for 3 days, including groups of control (gavaged with saline), Anti-LOX, HCG (human chorionic gonadotropin), VED, Anti-LOX + VED, HCG + VED, and Anti-LOX + HCG + VED.

Anti-LOX was applied by intragastric gavaged with a specific LOX inhibitor, *β*-aminopropionitrile (BAPN) fumarate (Shanghai Aladdin Biochemical Technology Co., Ltd., Shanghai, China) with a dose of 100 mg/kg/d^15,16,24,25^. HCG was intramuscularly administrated using a standard protocol of 100 IU/kg (3 times per week)^26–28^. VED meant the penis was stretched by a VED force with a negative aspiration pressure of -300 mmHg (Chengdu Xin Wei Cheng Technology Co., Ltd., Chengdu, China), the procedure was performed twice daily (each session lasting 5-min, with a 2-min interval) from Monday to Friday^15,16,29^. VED aspiration was ceased for 1 or 2 days when prepuce bleed was serious. In addition, groups with VED procedure were performed under isoflurane anesthesia, thus all the other groups including control were also anesthetized. The total intervention duration last for 4 weeks before the rats were used for the following analysis.

### Penile length measurement

On the last intervention day, rat’s penile length was measured after body weight was recorded. As described previously^15,16,30^, the injection end of a modified 2.5 mL disposable syringe was connected to VED device (Chengdu Xin Wei Cheng Technology Co., Ltd., Chengdu, China), the other side of syringe was placed over penis and tightly pressed to the pubis. After a unified aspirated precession (− 300 mmHg of 5 min for twice, with a 2 min interval), stretched penile length was read and recorded as a ruler close to the syringe flanged end.

### Erectile function assessment

After a 1-week washout period since penile length measurement, the erectile function was assessed as previously described^15,16,31–33^. Briefly, rat was anesthetized by isoflurane, then the cavernous never, penile crus, and carotid were successively exposed or isolated. As the cavernous never was electrically stimulated (5 V, 20 Hz, pulse width of 5.0 ms, for 50 s), the intracavernous pressure (ICP) in penile crus and mean arterial pressure (MAP) in carotid were simultaneously monitored by a BL420 biofunctional experiment system (TME Technology Co. Ltd. Chengdu, China). The maximum ICP/ MAP ratio was recorded and analyzed.

### Exposed penile length measurement

Exposed penile length was recorded after erectile function assessment. Briefly, the penis was completely exposed and vertically stretched with the tip of glans cartilage clipped by vessel forceps, until rat’s back/rump leave animal experimental table. Exposed penile length was measured from the junction of urethral bulb and corpus cavernosum to the tip of glans cartilage^15^.

Arterial blood was then collected from carotid artery for hormone detection, while the corpus cavernosum was washed with cold phosphate buffered saline (PBS) and cut into two sections (proximal and distal) for further analysis.

### Hormone detection

Arterial blood was clotted at room temperature for 2 h and centrifuged for serum (then immediately stored at − 80 °C). Serum samples were used to detect levels of HCG (Shanghai Enzyme-linked Biotechnology Co., Ltd), testosterone (E-EL-0155c; Elabscience Biotechnology Co. Ltd), and dihydrotestosterone (CSB-E07879r; CUSABIO BIOTECH CO., Ltd) by enzyme linked immunosorbent assay (ELISA) kits according to manufacturer’s instructions. The final density of each sample was determined by a microplate reader (450) nm, and hormone concentrations were calculated according affiliated standard curve.

### Western blot

The distal corpus cavernosum was snipped and homogenized in radio immunoprecipitation assay (RIPA) lysis buffer, and then centrifuged at 12000*g* for 20 min at 4 °C. The supernatant was collected and protein concentration was determined by Coomassie brilliant blue G-250 working buffer. Equal amount protein was loaded to 10% sodium dodecyl sulfate–polyacrylamide gel electrophoresis (SDS-PAGE) for electrophoresis, the protein were then wet-transferred to polyvinylidene difuoride membrane (Merck Millipore) according to standard procedures. After blocking by 5% non-fat dry milk in TBS-t, the membranes were cut into pieces (referred to molecular weighs as preliminary experiment) and incubated with primary antibodies of anti-endothelial nitric oxide synthases (anti-eNOS) (1:1000, Abcam), anti-a-smooth muscle actin (anti-α-SMA) (1:1000, Abcam), and anti-androgen receptor (anti-AR) (1:1000, Abcam) for 24 h (at 4℃). The secondary antibody of *β*-Actin (1:200, Zen BioScience Co., Ltd. Chengdu, Sichuan Province, China) was incubated after membranes were washed. The densitometry of protein band was collected by Bio-Rad ChemiDoc MP (Bio-Rad, Berkeley, CA, USA) and its intensities were quantified by Image J sofware (National Institute of Health, Bethesda, MD, USA).

### LOX activity

Activity of LOX protein was determined by Amplite™ Fluorimetric LOX assay kit (AAT Bioquest Inc., Sunnyvale, CA, USA) as previous described^15,16,34,35^. Briefly, the proximal corpus cavernosum was finely snipped and homogenized in PBS (at 4 °C) and centrifuged at 10000*g* for 30 min to obtain supernatants. After standard procedures according the manufacturer’s instruction, the fluorescence was recorded using BioTek Synergy Mx (BioTek Instruments Co., Ltd., Winooski, VT, USA) with excitation and emission wavelengths at 560 and 590 nm, respectively. The activity of LOX protein was normalized and expressed as RFUs/ug protein.

### Statistical analyses

All results were shown as mean ± SD and analyzed by GraphPad Prism 5.0 (GraphPad Sofware, San Diego, CA, USA). For statistical differences among multiple groups, one-way ANOVA analysis was used and followed by Tukey’s test to compare all pairs of columns. Student’s t-test was performed to obtain p value for some groups while a *p* < 0.05 was considered significant.

## Results

### Primary and final body weight

As was shown, no significant difference was found for rats’ primary and final body weight (*p* > 0.05). Although HCG, HCG + VED, and Anti-LOX + HCG + VED dramatically decreased bilateral testicular weight when compared with control, Anti-LOX, VED, and Anti-LOX + VED (*p* < 0.05), no other difference was found (Fig. S1).

### Penile length

**Figure 1:**
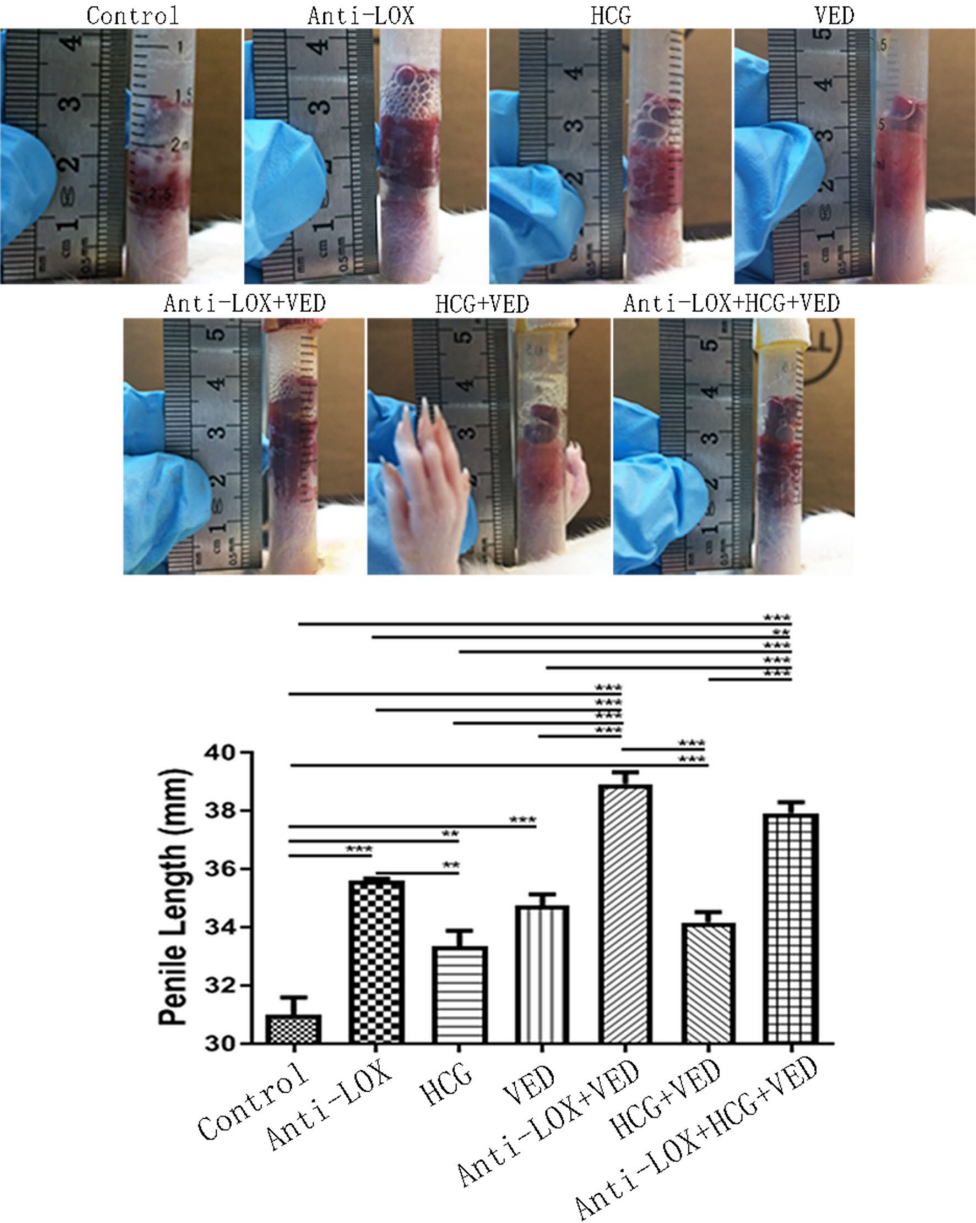
Representative images of penile length and statistical analysis of penile size. Penile length were measured after a unified VED aspiration (− 300 mmHg for 5 min for twice, with 2 min interval). * < 0.05, ** < 0.01, *** < 0.001.

All experimental rats showed significantly longer penis than control group (31.00 ± 1.32 mm). Specifically, Anti-LOX (35.58 ± 0.19 mm), HCG (33.33 ± 1.21 mm), VED (34.75 ± 0.85 mm), Anti-LOX + VED (38.92 ± 0.89 mm), HCG + VED (34.17 ± 0.80 mm), and Anti-LOX + HCG + VED (37.92 ± 0.84 mm) dramatically lengthened penis (*p* < 0.05) by 15% (4.58 mm), 8% (2.33 mm), 12% (3.75 mm), 26% (7.92 mm), 10% (3.17 mm), and 22% (6.92 mm), respectively. Interestingly, Anti-LOX + VED and Anti-LOX + HCG + VED presented the longest penises which were longer than the other five groups (*p* < 0.05), however, no significant difference was found between these two groups (*p* > 0.05). Moreover, HCG showed a similar penile length with VED (*p* > 0.05) but was significantly shorter than Anti-LOX (*p* < 0.05), while HCG combined with VED (HCG + VED) could not lengthen penis than Anti-LOX or VED group (*p* > 0.05) (Fig. 1, Tables 1, 2).

**Table 1:** Data collection.

	Control	Anti-LOX	HCG	VED	Anti-LOX + VED	HCG + VED	Anti-LOX + HCG + VED	*p*
Primary weight (g)	132.17 ± 1.77	131.67 ± 1.25	132.50 ± 2.63	132.00 ± 1.83	136.67 ± 3.45	133.33 ± 2.36	134.50 ± 5.44	0.1292
Final weight (g)	264.67 ± 12.41	269.17 ± 11.54	268.50 ± 6.65	258.50 ± 12.61	253.17 ± 7.73	249.33 ± 18.94	246.33 ± 9.66	0.0185
Testicular weight (g)	16.05 ± 1.00	15.66 ± 0.60	11.83 ± 2.26	15.95 ± 1.39	16.35 ± 0.73	11.08 ± 2.24	9.93 ± 2.68	< 0.0001
Penile length (mm)	31.00 ± 1.32	35.58 ± 0.19	33.33 ± 1.21	34.75 ± 0.85	38.92 ± 0.89	34.17 ± 0.80	37.92 ± 0.84	< 0.0001
Exposed penile length (mm)	28.08 ± 0.53	30.50 ± 0.65	28.83 ± 0.69	30.75 ± 0.25	33.42 ± 0.89	31.17 ± 1.55	33.25 ± 0.80	< 0.0001
ICP	63.45 ± 14.70	71.89 ± 5.11	61.21 ± 8.31	79.61 ± 23.17	82.54 ± 18.61	68.06 ± 11.44	77.02 ± 12.33	0.1824
MAP	114.21 ± 10.48	111.87 ± 12.18	121.70 ± 8.40	130.75 ± 12.44	125.31 ± 24.69	118.00 ± 11.26	114.06 ± 18.32	0.4036
ICP/MAP	0.56 ± 0.12	0.65 ± 0.06	0.51 ± 0.08	0.60 ± 0.16	0.65 ± 0.06	0.58 ± 0.05	0.68 ± 0.04	0.0540

**Table 2:** Penile length comparison in relative and percentage value [(Longer-Shorter)/Shorter].

Penile length	Control	Anti-LOX	HCG	VED	Anti-LOX + VED	HCG + VED
Anti-LOX (mm, %)	4.58 (15%)	–	–	–	–	–
HCG (mm, %)	2.33 (8%)	2.25 (7%)	–	–	–	–
VED (mm, %)	3.75 (12%)	0.83 (2%)	1.42 (4%)	–	–	–
Anti-LOX + VED (mm, %)	7.92 (26%)	3.33 (9%)	5.58 (17%)	4.17 (12%)	–	–
HCG + VED (mm, %)	3.17 (10%)	1.42 (4%)	0.83 (2%)	0.58 (2%)	4.75 (14%)	–
Anti-LOX + HCG + VED (mm, %)	6.92 (22%)	2.33 (7%)	4.58 (14%)	3.17 (9%)	1.0 (3%)	3.75 (11%)

### Exposed penile length

**Figure 2:**
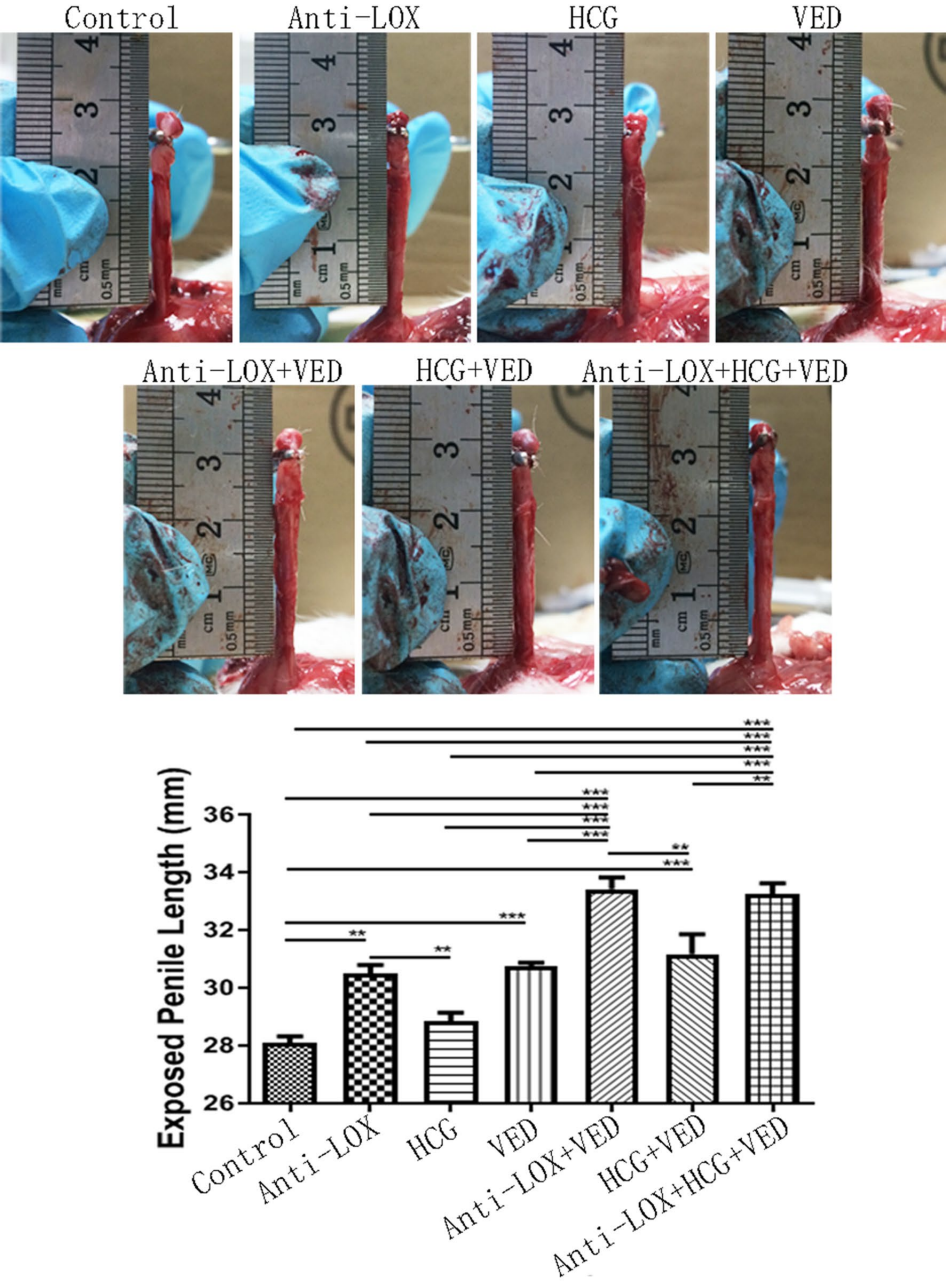
Representative images of exposed penile length and statistical analysis of exposed size. * < 0.05, ** < 0.01, *** < 0.001.

A similar trend was found for exposed penile length when compared with control group (28.08 ± 0.53 mm). That Anti-LOX (30.50 ± 0.65 mm), HCG (28.83 ± 0.69 mm), VED (30.75 ± 0.25 mm), Anti-LOX + VED (33.42 ± 0.89 mm), HCG + VED (31.17 ± 1.55 mm), and Anti-LOX + HCG + VED (33.25 ± 0.80 mm) significantly increased exposed length (*p* < 0.05) by 9% (2.42 mm), 3% (0.75 mm), 9% (2.67 mm), 19% (5.33 mm), 11% (3.08 mm), and 18% (5.17 mm), respectively. Exposed penile length in HCG group was still similar with VED (*p* > 0.05) but significantly shorter than Anti-LOX (*p* < 0.05), while HCG + VED also showed similar exposed length with Anti-LOX or VED group (*p* > 0.05). Finally, there was no significant difference for exposed penile size between Anti-LOX + VED and Anti-LOX + HCG + VED groups (*p* > 0.05) (Fig. 2, Tables 1, 3).

**Table 3:** Exposed penile length comparison in relative and percentage value [(Longer-Shorter)/Shorter].

Exposed penile length	Control	Anti-LOX	HCG	VED	Anti-LOX + VED	HCG + VED
Anti-LOX (mm, %)	2.42 (9%)	–	–	–	–	–
HCG (mm, %)	0.75 (3%)	2.42 (9%)	–	–	–	–
VED (mm, %)	2.67 (9%)	0.25 (1%)	2.67 (9%)	–	–	–
Anti-LOX + VED (mm, %)	5.33 (19%)	2.92 (10%)	5.33 (19%)	2.67 (9%)	–	–
HCG + VED (mm, %)	3.08 (11%)	0.67 (2%)	3.08 (11%)	0.42 (1%)	2.25 (7%)	–
Anti-LOX + HCG + VED (mm, %)	5.17 (18%)	2.75 (9%)	5.17 (18%)	2.50 (8%)	0.17 (1%)	2.08 (7%)

### Erectile function assessment: ICP and ICP/MAP ratio

**Figure 3:**
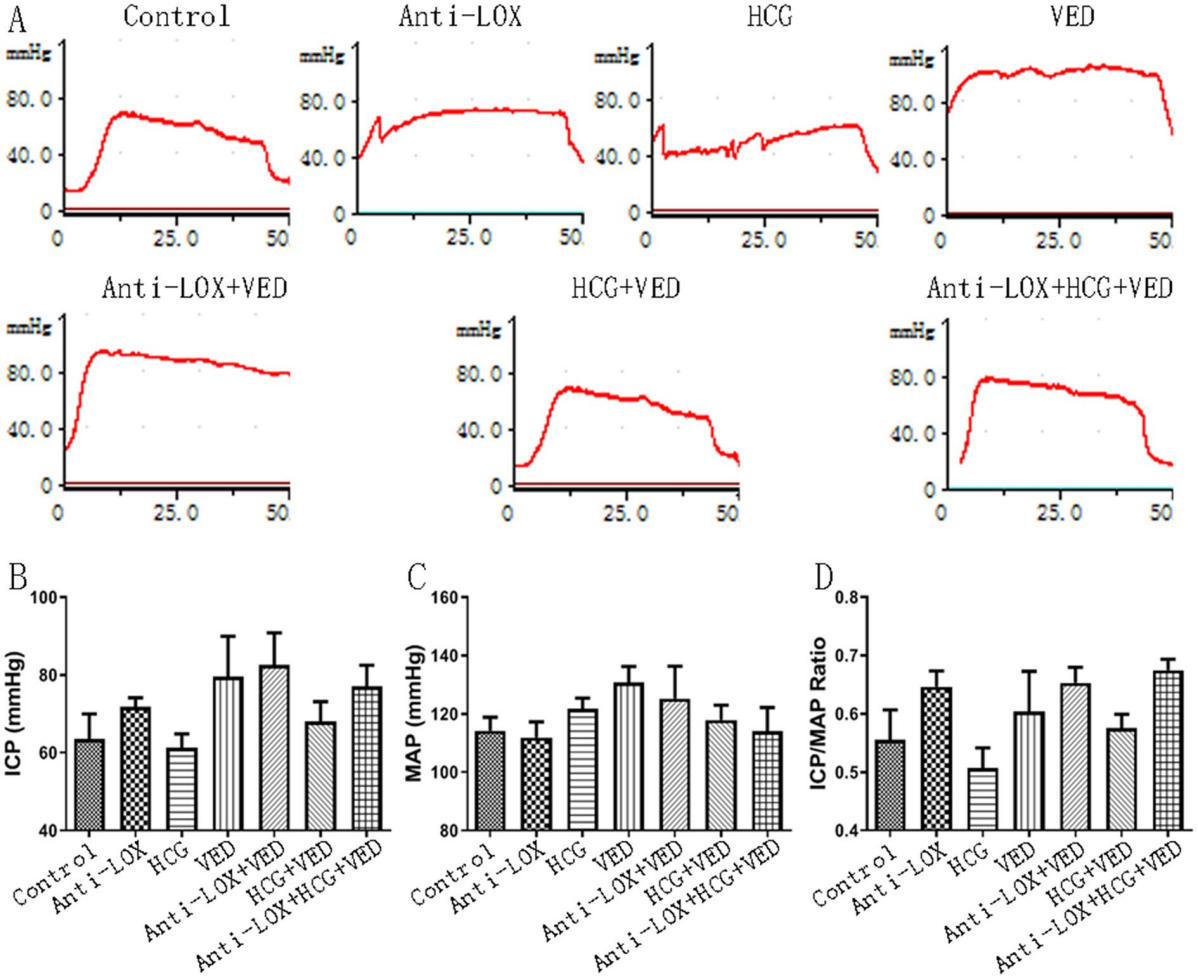
Androgen supplement combined with Anti-LOX + VED had no impact on normal erectile function, whether alone or in combination. (**A**) Representative images of maximum ICP under cavernous nerve stimulation. (**B**) Statistical analysis of ICP. (**C**) Statistical analysis of MAP. (**D**) Statistical analysis of ICP/MAP ratio.

Although Anti-LOX, VED, Anti-LOX + VED, HCG + VED, and Anti-LOX + HCG + VED presented slightly higher ICP and ICP/MAP ratio than control and HCG groups, no significant difference was found (*p* > 0.05). The MAP was basically similar among the seven groups (*p* > 0.05) (Fig. 3, Table 1).

### Erectile function related biomarker: eNOS and α-SMA

**Figure 4:**
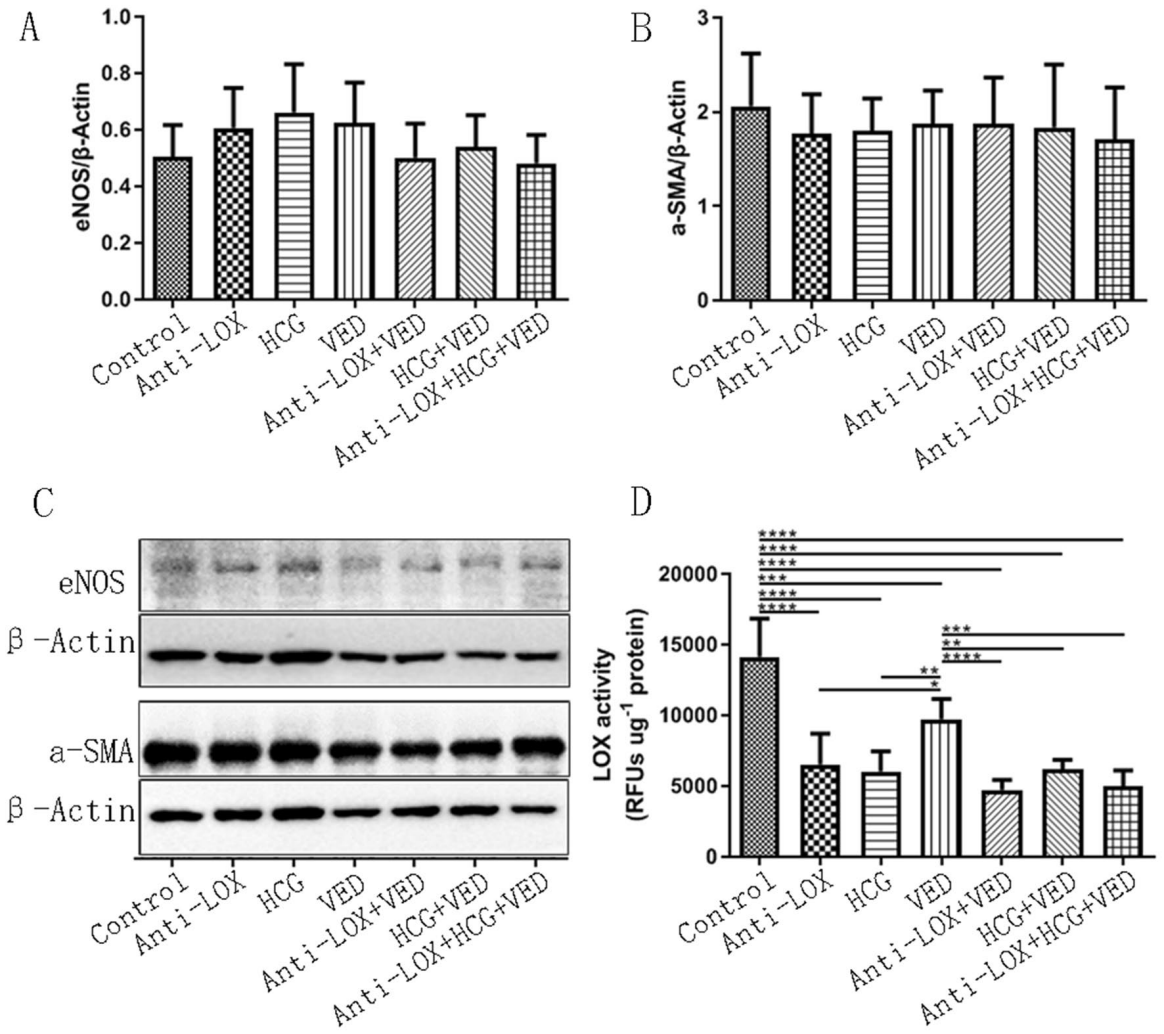
(**A**, and **B**) Statistical analysis of eNOS and α-SMA protein expression. (**C**) Representative images of eNOS and α-SMA expression. (**D**): Statistical analysis of LOX activity. * < 0.05, ** < 0.01, *** < 0.001.

As two classical molecular biomarker for erectile function assessment, eNOS and α-SMA were all similar among the seven groups, indicating penile erectile function was not impaired by Anti-LOX, HCG, or VED, whether alone or in combination (*p* < 0.05) (Fig. 4).

### LOX activity

As was shown, LOX activity in control group was the greatest and significantly higher than control, Anti-LOX, VED, Anti-LOX + VED, HCG + VED, and Anti-LOX + HCG + VED groups (*p* < 0.05). While VED also revealed higher LOX activity than Anti-LOX, Anti-LOX + VED, HCG + VED, and Anti-LOX + HCG + VED groups (*p* < 0.05) (Fig. 4).

### Hormone and AR level

**Figure 5:**
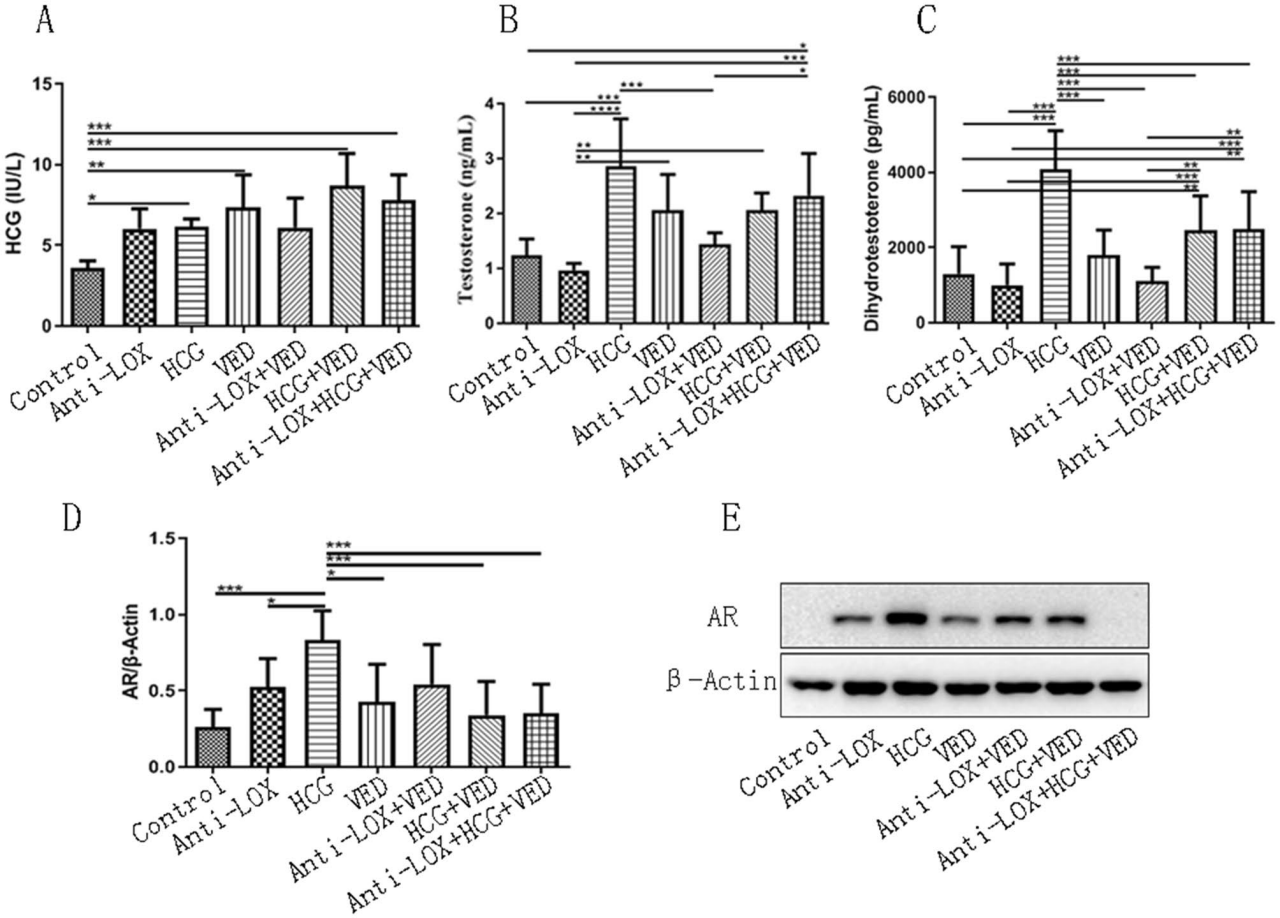
(**A**, **B**, and **C**) Statistical analysis of HCG, testosterone, and dihydrotestosterone, respectively. (**D** and **E**): Statistical analysis of androgen receptor (AR) protein expression and representative WB images. * < 0.05, ** < 0.01, *** < 0.001.

For HCG concentration, the HCG, VED, HCG + VED, Anti-LOX + HCG + VED groups were significantly higher than control group (*p* < 0.05), no other significantly difference was found among other groups (*p* > 0.05) (Fig. 5).

For testosterone level, HCG group was significantly higher than control, Anti-LOX, and Anti-LOX + VED groups (*p* < 0.05). Anti-LOX + HCG + VED group showed increased testosterone concentration that control, Anti-LOX, and Anti-LOX + VED groups (*p* < 0.05). Moreover, VED and HCG + VED also revealed more testosterone level than Anti-LOX group (*p* < 0.05) (Fig. 5).

As dihydrotestosterone secretion, HCG group presented higher concentration than control, Anti-LOX, VED, Anti-LOX + VED, HCG + VED, and Anti-LOX + HCG + VED groups (*p* < 0.05). While the HCG + VED and Anti-LOX + HCG + VED all demonstrated more dihydrotestosterone levels than control, Anti-LOX and Anti-LOX + VED groups (*p* < 0.05) (Fig. 5).

The WB analysis finally found that AR expression in HCG groups was the highest and significantly increased than the other six groups (*p* < 0.05), however, no significantly difference was found among other groups (*p* < 0.05) (Fig. 5).

## Discussion

Just like women pursue a bigger breast, men always desire a larger penis not only to impress partners but also to improve self-esteems^1,3^. Considering the huge personal requirements, more authors suggested to reappraise phalloplasties as an esthetic plastic surgery rather than reconstruction surgery^4^. However, the phalloplasties are controversial^6^ and experimental^1,5^ as the poorly standardized indications and inconsistent surgical methods^5,6^. So, it will be interesting and necessary to explore new strategies to lengthen penis.

We know that penile tunica albuginea which mainly composed by thick collagen bundles and some elastic fibers determines penile size during tumescence^13–16^. As an extracellular copper-dependent monoamine oxidase, Lysyl oxidase (LOX) catalyzes the crosslinking of collagen and elastin proteins into insoluble mature fibers to contribute tensile strength of collagen fiber and elasticity of elastin fiber^15,16,34,36–38^. Our previous study found that inhibiting LOX activity (Anti-LOX) promoted tunica albuginea remodeling by reducing collagen crosslinking, which finally increased penile length by 10.54% for adult rats, this effect achieved 17.71% when Anti-LOX was combined to a mechanical force from VED (Anti-LOX + VED)^15^. The lengthening effect was more obvious for pubertal rats, that Anti-LOX and Anti-LOX + VED significantly promoted penile length by 10.79% and 19.84%, respectively^16^.

Similar results were found for pubertal rats in this study, that Anti-LOX and Anti-LOX + VED lengthened penis by 15% and 26%, respectively. We speculated that this final penile length was measured under a VED pressure at − 300 mmHg, thus the size was longer than previous pubertal rats which measured at − 200 mmHg (10.79% and 19.84%)^16^. We further confirmed the lengthening effect by measuring exposed penile size, that Anti-LOX and Anti-LOX + VED increased length by 9% and 19%, respectively. Moreover, LOX activity in Anti-LOX, Anti-LOX + VED, and Anti-LOX + HCG + VED groups were all inhibited than control group. Combined with our previous researches^15,16^, we concluded the penile length was improved by remodeling tunica albuginea.

Testosterone is indispensable for penile development^11,19,39,40^ and androgen-dependent growth is responsible for 70–75% of adult penile length^19,41,42^. It is reported that testosterone level transient rises in the first 4–6 months after birth and then stabilizes less than 25 ng/dL during infancy and childhood, while penis only grows to 3 cm before 11 years old^9^. The activation of hypothalamic-pituitary-testicular axis in puberty induces expression of androgen receptor (AR) and stimulates secretion of testosterone and DHT^9,43,44^, which finally lead the penile growth spurt^9,43,45^. Disruption of androgen pathway thus inevitably attenuates penile growth and leads congenital micropenis, which affects up to 0.7% of newborn males^39^. Considering its critically role on penile development, testosterone supplement has been proven to improve penile growth and androgen replacement was recommended for hypogonadotropic hypogonadal micropenis since the 1970s^9,12,19,21,22^, although still accompanied with some controversy.

As the only FDA approved non-testosterone compounds for testosterone deficiency^23^, HCG stimulates endogenous testosterone production^23^ without impairing spermatogenesis^23,46^. We thus selected HCG rather than testosterone supplement which found that HCG administration (100 mg/kg twice per week for 1 month) significantly elevated testosterone and dihydrotestosterone levels, which was constant with previous studies^23,47,48^. For adolescent micropenis, Rajendra et al. revealed that HCG (1500–2000 IU once per week for 6 weeks) increased stretched penile length from 15.54 to 37.18 mm, while testosterone (25 mg once a month for 3 months) lengthened it from 26.42 to 64.28 mm^49^. In this study, we demonstrated that HCG significantly lengthened penis by 8%, however, no difference was observed for exposed penile size (3%). Some researchers also proved that postnatal testosterone therapy only advanced penile growth rather than improved the final length^17^, while others even claimed testosterone exposure in puberty accelerated AR loss and compromised eventual penile length in adulthood^15,50–54^. Authors inferred that androgen determined penile development in specific “masculinisation programming window” (MPW) while subnormal androgen could not be reversed by later supplement^17,19^. Moreover, penile growth involved not only androgen-dependent processes but also some independent procedures^17,19,55^ that 6/13 individual steps for human penile development and 5/11 for mice were reported to be androgen-independent^55^, while these nonandrogenic hormones like thyroxin, glucocorticoids or growth hormone might not be corrected simply by testosterone administration^56^. In conclusion, androgen replacement was not recommended to lengthen penis for healthy male alone considering the controversial effects and accompanied complications^50,51^.

Even though, we still explored whether HCG supplement synergized with Anti-LOX + VED could create miracle and promote penile growth, considering their respectively essential roles in penile development. As a result, although Anti-LOX + HCG + VED significantly lengthened penis by 22%, it was less effective than Anti-LOX + VED (26%), while the exposed penile length in Anti-LOX + HCG + VED (18%) was also shorter than Anti-LOX + VED (19%). These suggested that HCG supplement did not cooperate with Anti-LOX + VED to promote penile growth.

Ma et al.^19^ has reported that testosterone induced AR expression to dose-dependent stimulate penile growth. Although we found HCG increased AR level, some inconsistent conclusions have also been observed^19,57–60^. For instance, Gonzalez-Cadavid et al. found that androgen improved *AR* mRNA level in rat penile smooth-muscle cells^19,59^ but Takane et al.^58^ shown that DHT declined it, while Ma et al. claimed that neither testosterone nor DHT was the major factor to physiologically down-regulate AR in corpora cavernosa^19,57^. Moreover, Baskin et al. demonstrated that AR positive cells in human fetal penis was similar among normal, castrate, and super testosterone hosts, while they concluded that testosterone might regulated penile growth by extracellular stromal expansion^53^. As the limited value of testosterone on penile lengthening, we did not further analyze why HCG + VED and Anti-LOX + HCG + VED did not improve AR level.

What’s more, VED and HCG + VED lengthened penis by 12% and 10%, respectively; while increased exposed penile size by 9% and 11%, respectively. These proved that HCG replacement combined a VED force did not promote penile growth, suggesting androgen was not an effective supplement for the “first line” non-invasive penile lengthening technique^9^. Finally, Anti-LOX, HCG, and VED did not increase or decrease ICP and ICP/MAP ratio, neither alone nor in combination. Meanwhile they did not improve or deteriorate the levels of eNOS and α-SMA, indicating these procedures had no impacts on erectile function.

Even though, our study has several limitations. Firstly, androgen was essential for penile development^19,39,41,42^, meanwhile we previous demonstrated Anti-LOX + VED lengthened penis by remodeling tunica albuginea^15,16^. We thus explored whether HCG supplement synergized with Anti-LOX + VED to promote penile growth by accelerating tunica albuginea. However, our negative results suggested the simply combination might not achieve an effect of “1 + 1 = 2”. Secondly, androgen therapy was recommended to improve penile length for micropenis^9,12,15,19,21,22^ but not for normal individuals, more attention should be paid to clarify the unclear underlying mechanism. Finally, although we tried to explore new procedures to lengthen penis, intensive psychosexual counselling was still primary recommended before some safe and effective procedures were demonstrated.

## Conclusion

We confirmed that Anti-LOX promoted penile growth, especially when combined with a VED force. Although HCG administration slightly lengthened penis by stimulating testosterone and dihydrotestosterone secretion, the underlying mechanism was not clarified. Moreover, HCG supplement did not synergize with Anti-LOX + VED to accelerate tunica albuginea remodeling nor facilitate penile growth, although they had no impact on erectile function.

### Supplementary Information

Supplementary Information 1.

Supplementary Information 2.

Supplementary Information 3.

Supplementary Information 4.

Supplementary Figure S1.

## Data Availability

All data generated or analysed during this study are included in this published article [and its supplementary information files.
