# Supplementary material for: HCG supplement did not accelerate tunica albuginea remodeling to facilitate penile growth
Source: Sci Rep. 2023 Oct 2;13:16519. doi: 10.1038/s41598-023-38888-y (PMC10545796; doi:10.1038/s41598-023-38888-y)

Raw data for WB analysis of AR, eNOS, and aSMA

2022.04.29 AR

A	B	C	D	E	F	G
Control	Anti-LOX	HCG	-300	-300+Anti-LOX	-300+HCG	-300+Anti-LOX+HCG

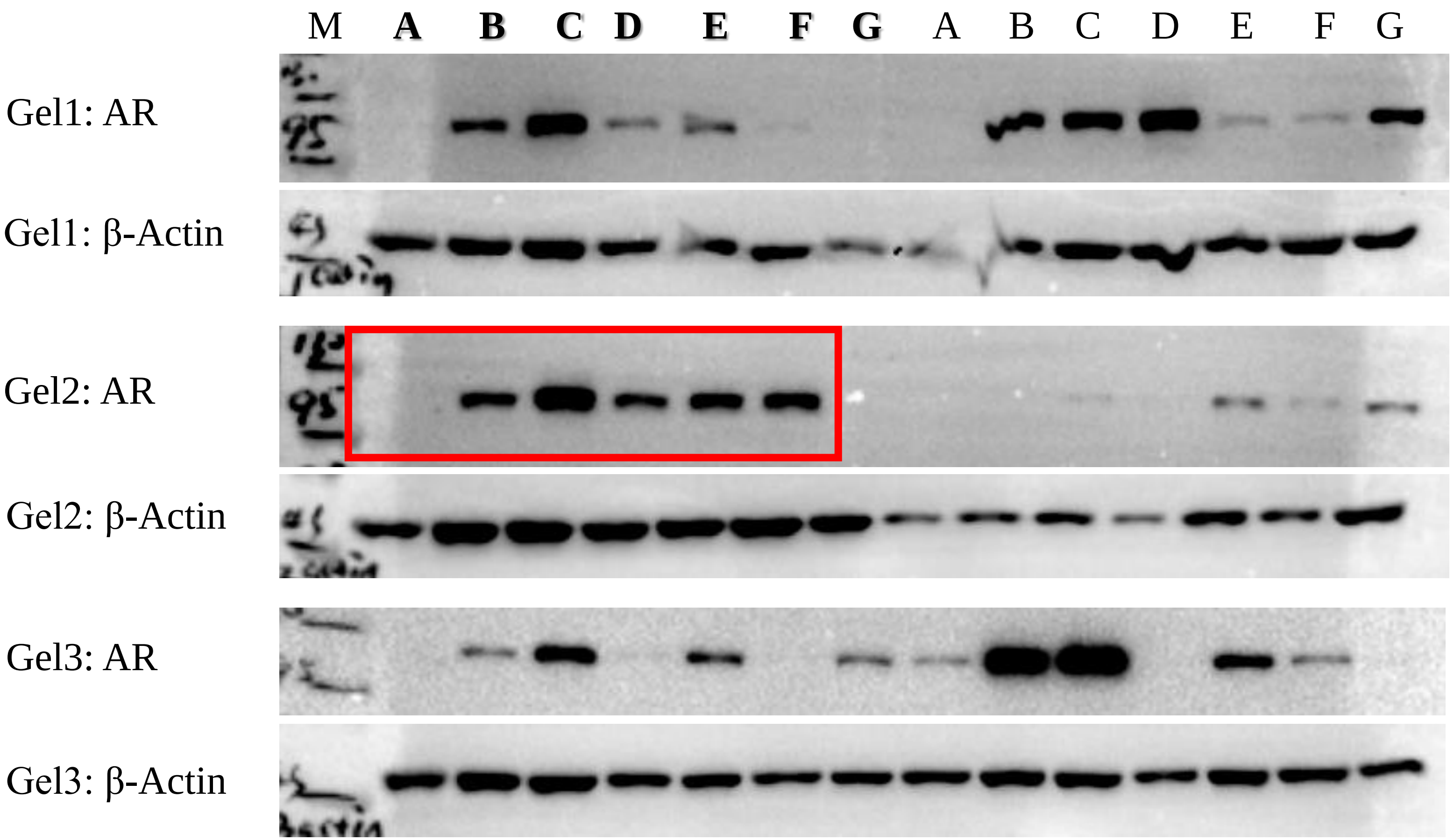

2022.05.31

eNOS

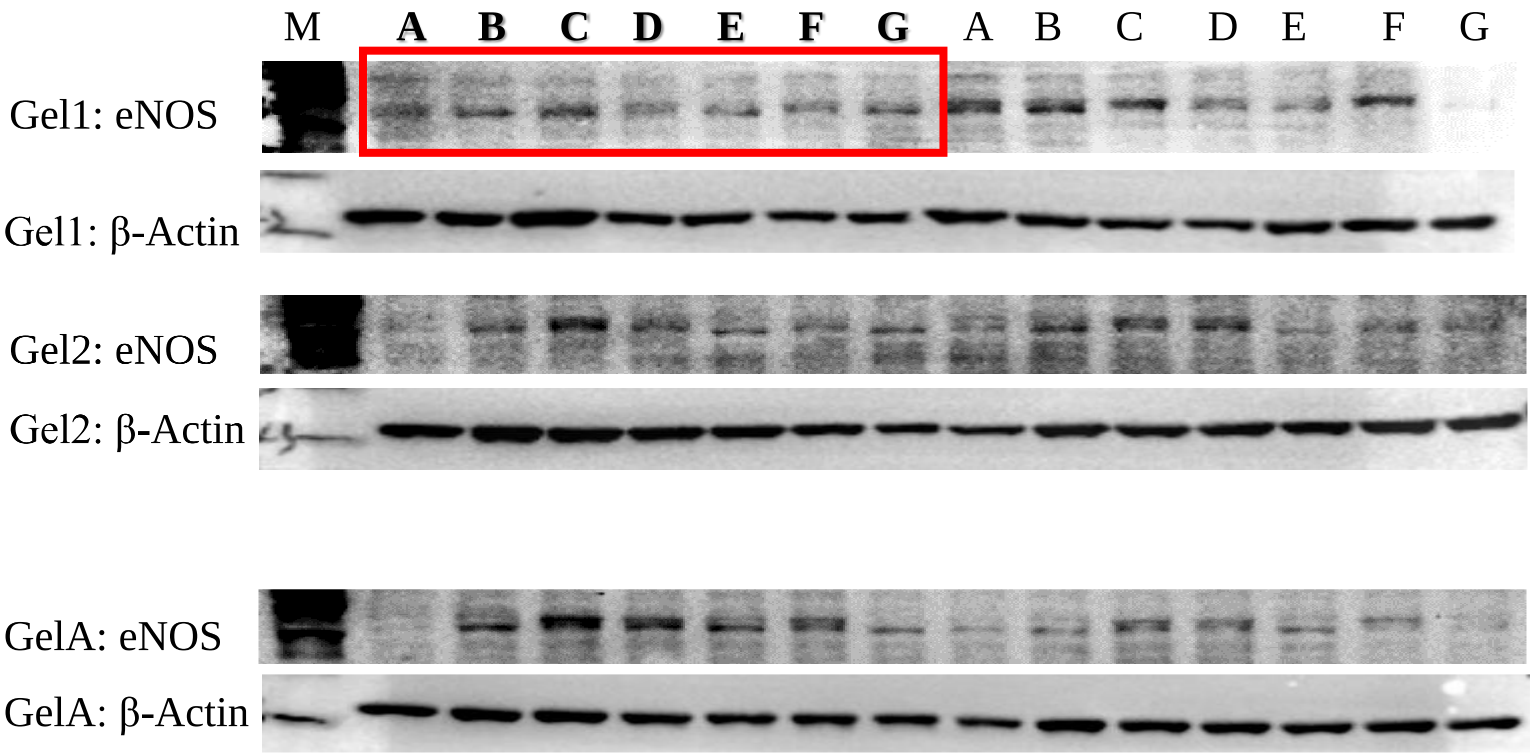

2022.05.06 **α-SMA**

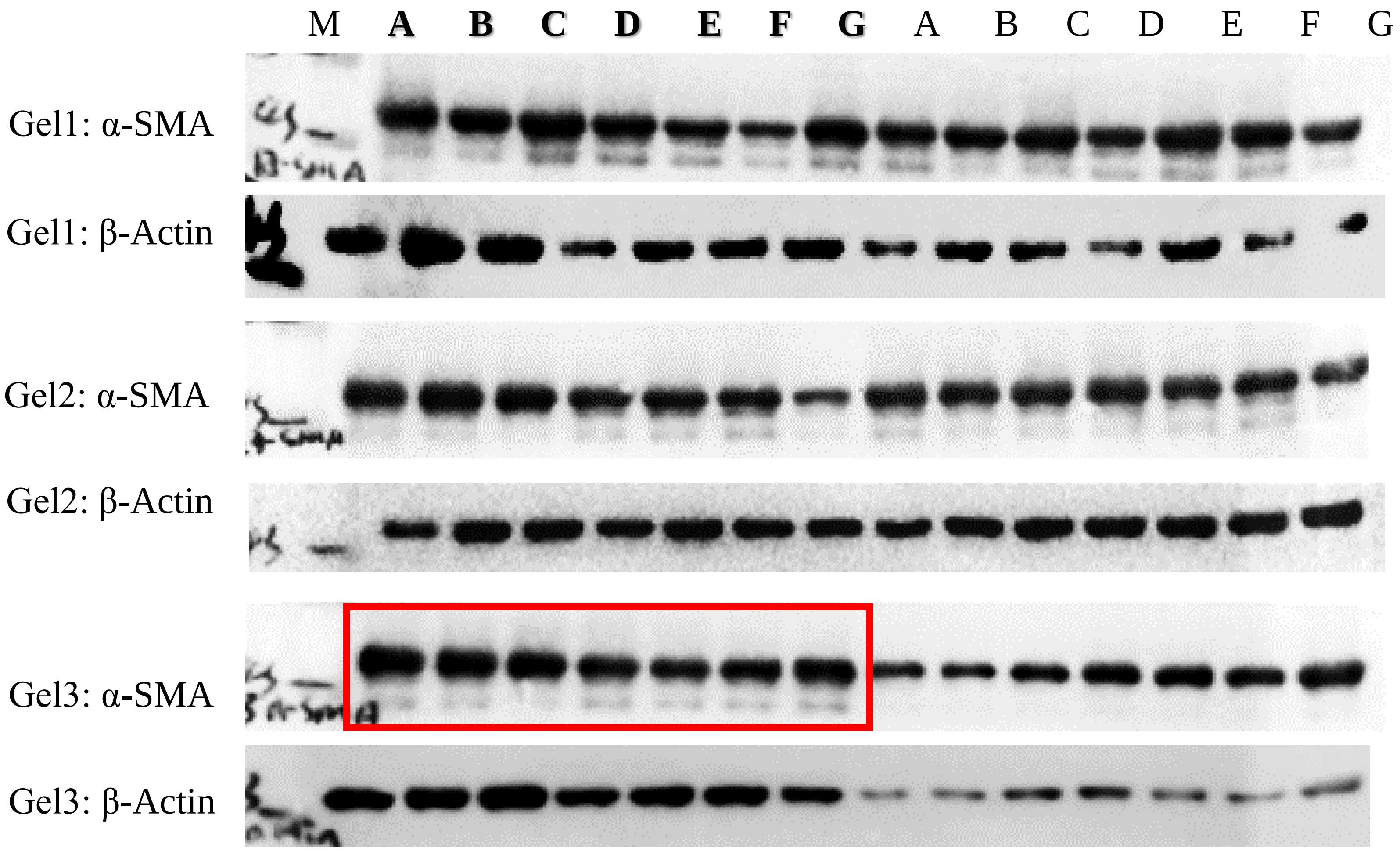

Supplement: Supplementary file 2 — Supplementary Information 2. [file 41598_2023_38888_MOESM2_ESM.pdf]
