# Supplementary material for: HCG supplement did not accelerate tunica albuginea remodeling to facilitate penile growth
Source: Sci Rep. 2023 Oct 2;13:16519. doi: 10.1038/s41598-023-38888-y (PMC10545796; doi:10.1038/s41598-023-38888-y)

Control 1

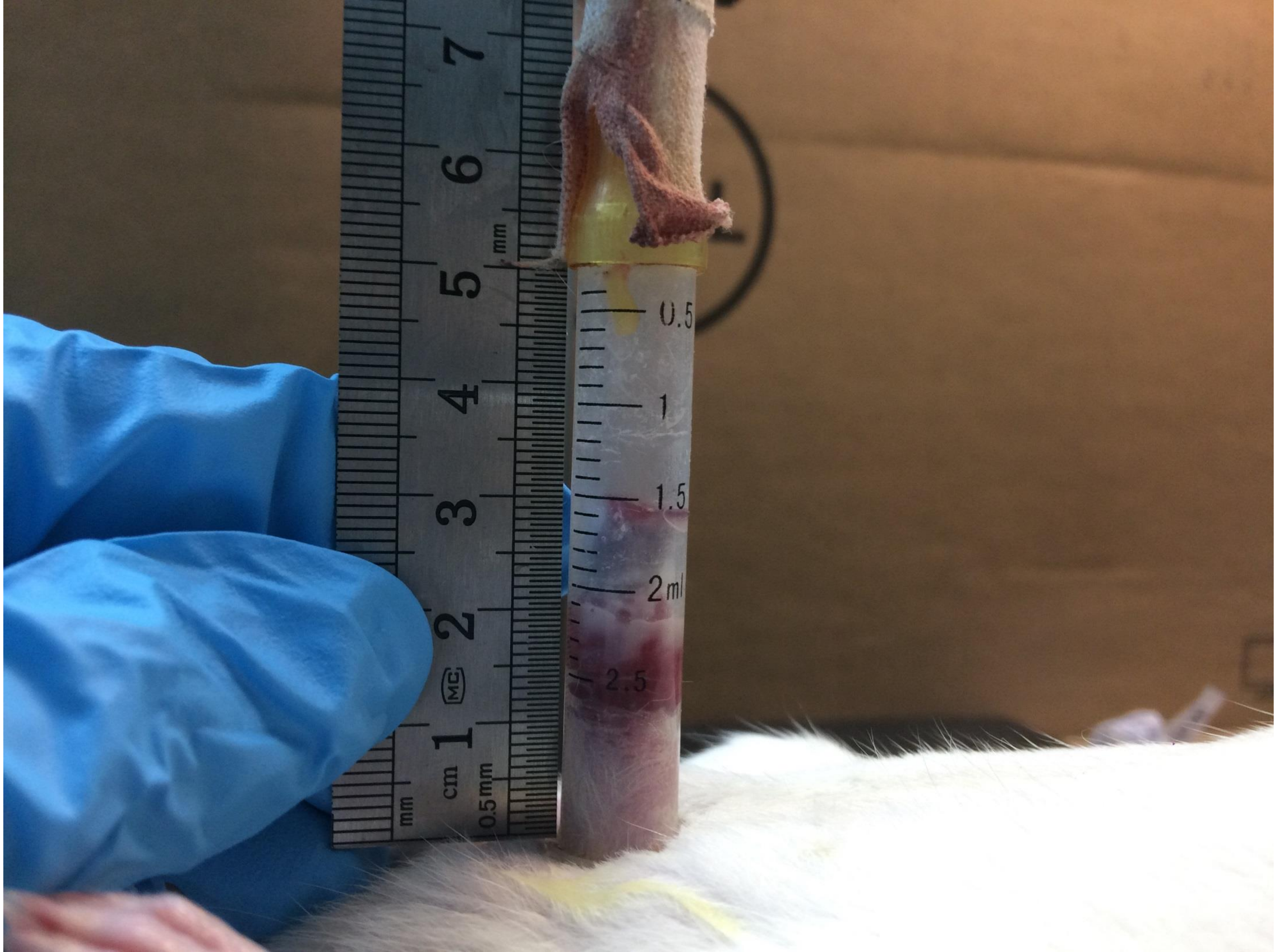

Control 2

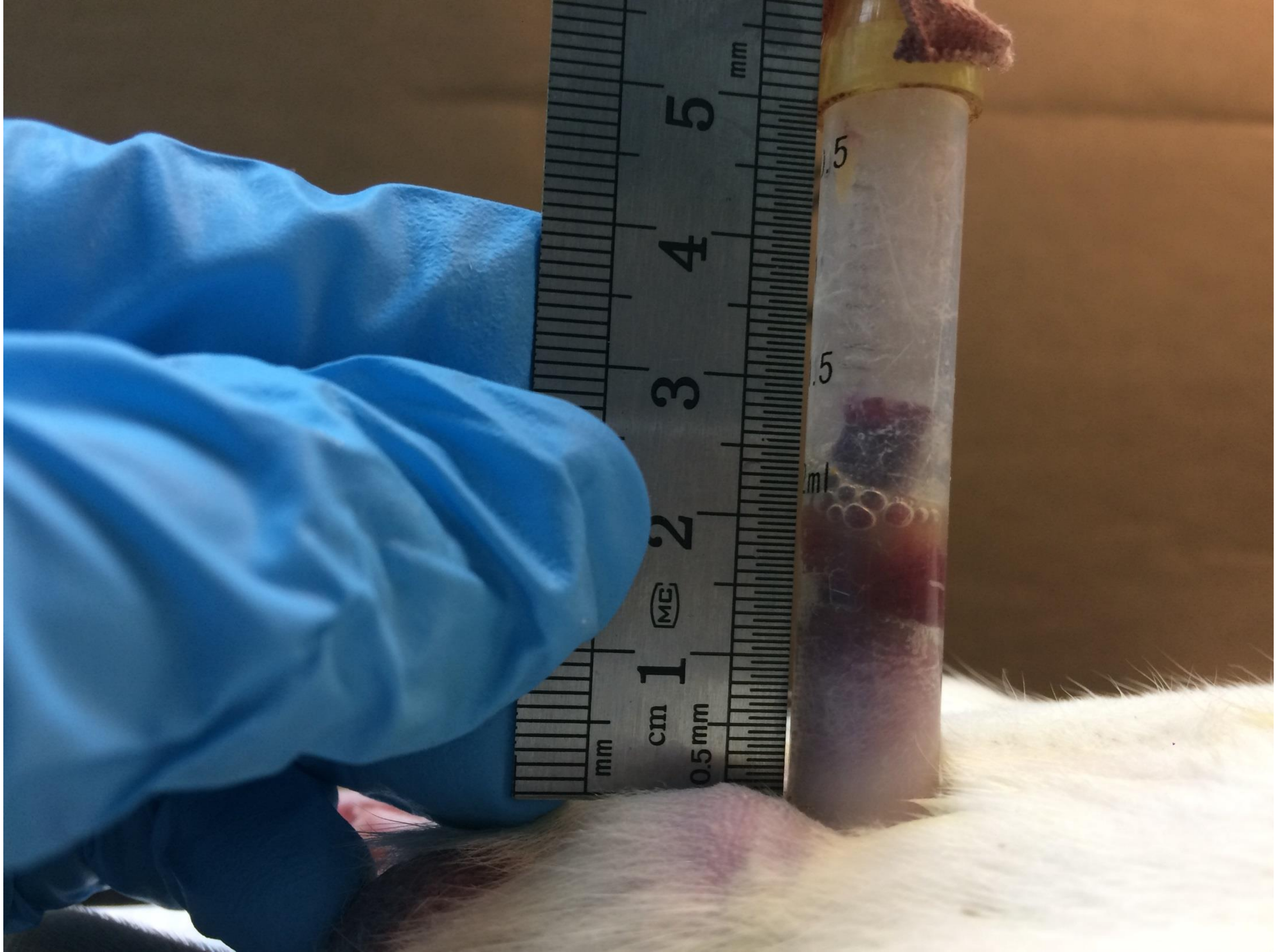

Control 3

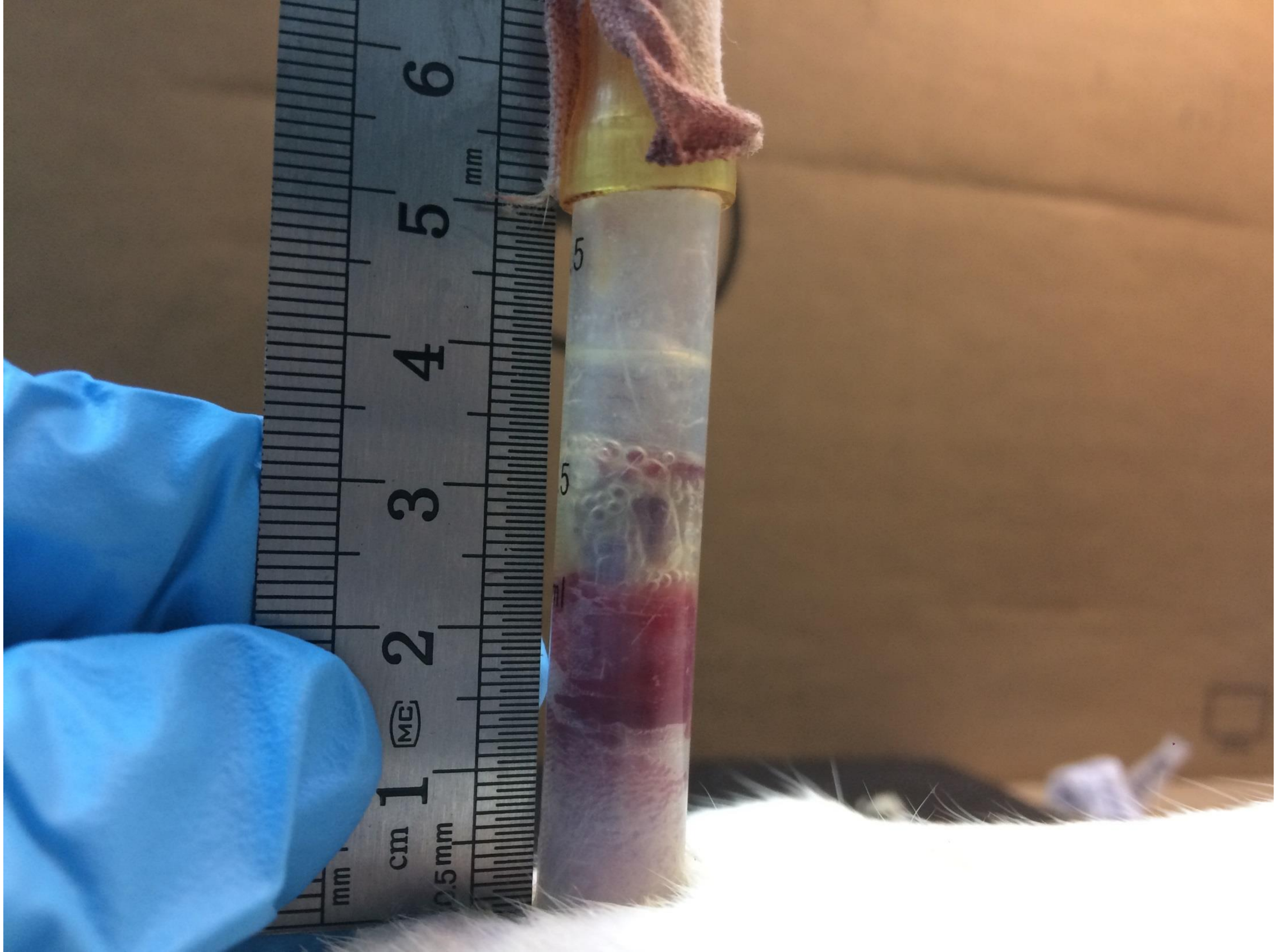

Control 4

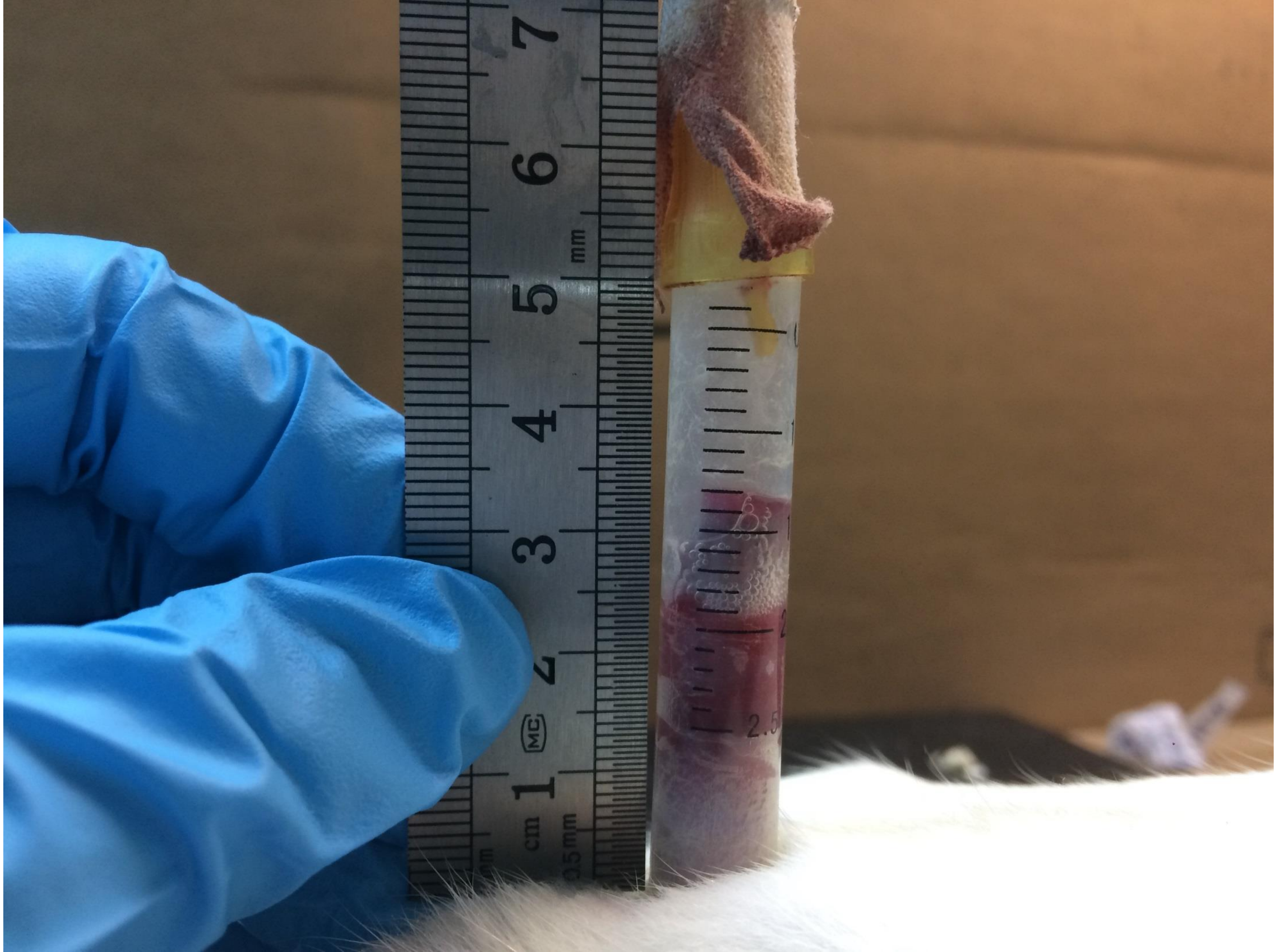

Control 5

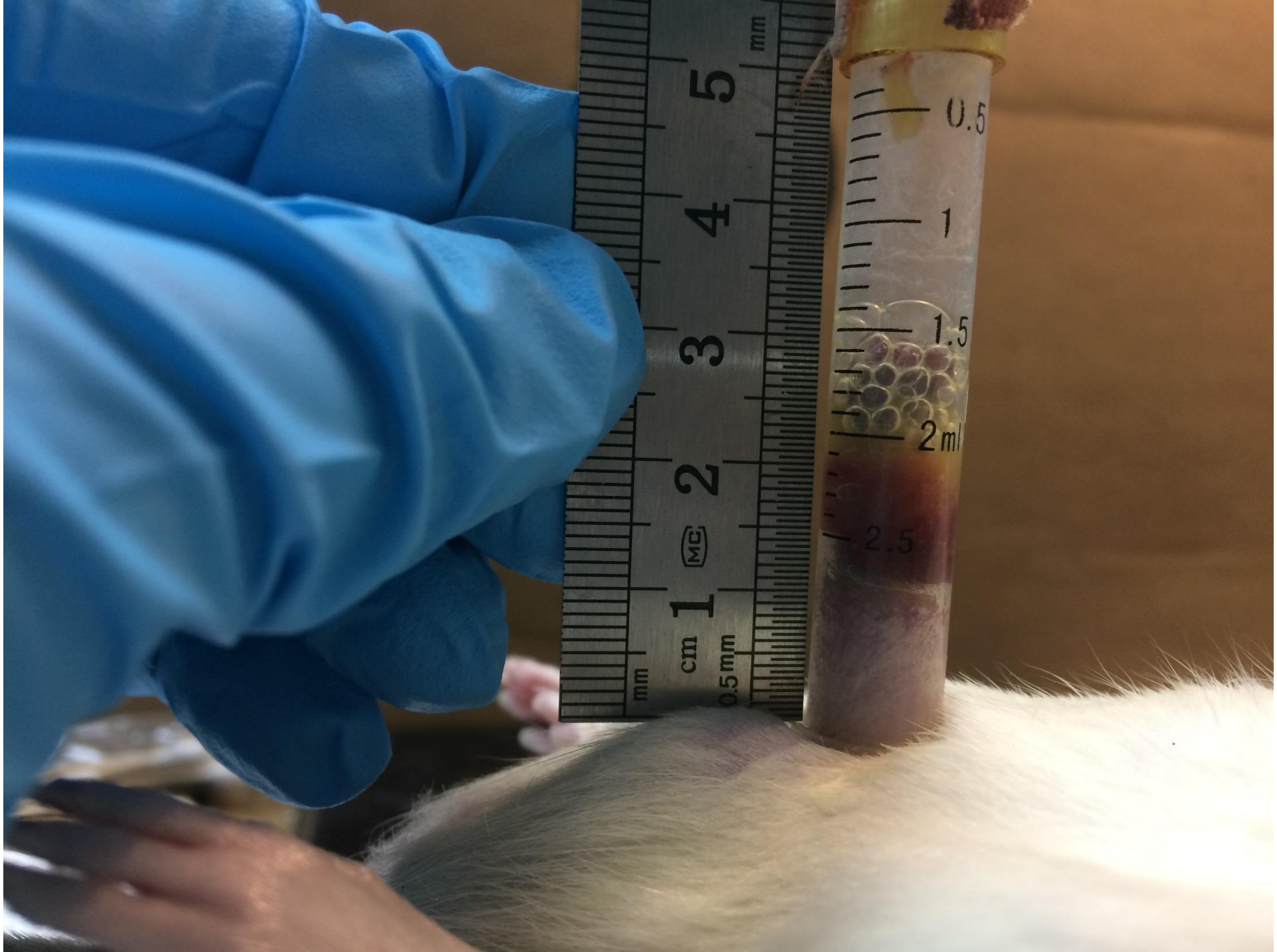

Control 6

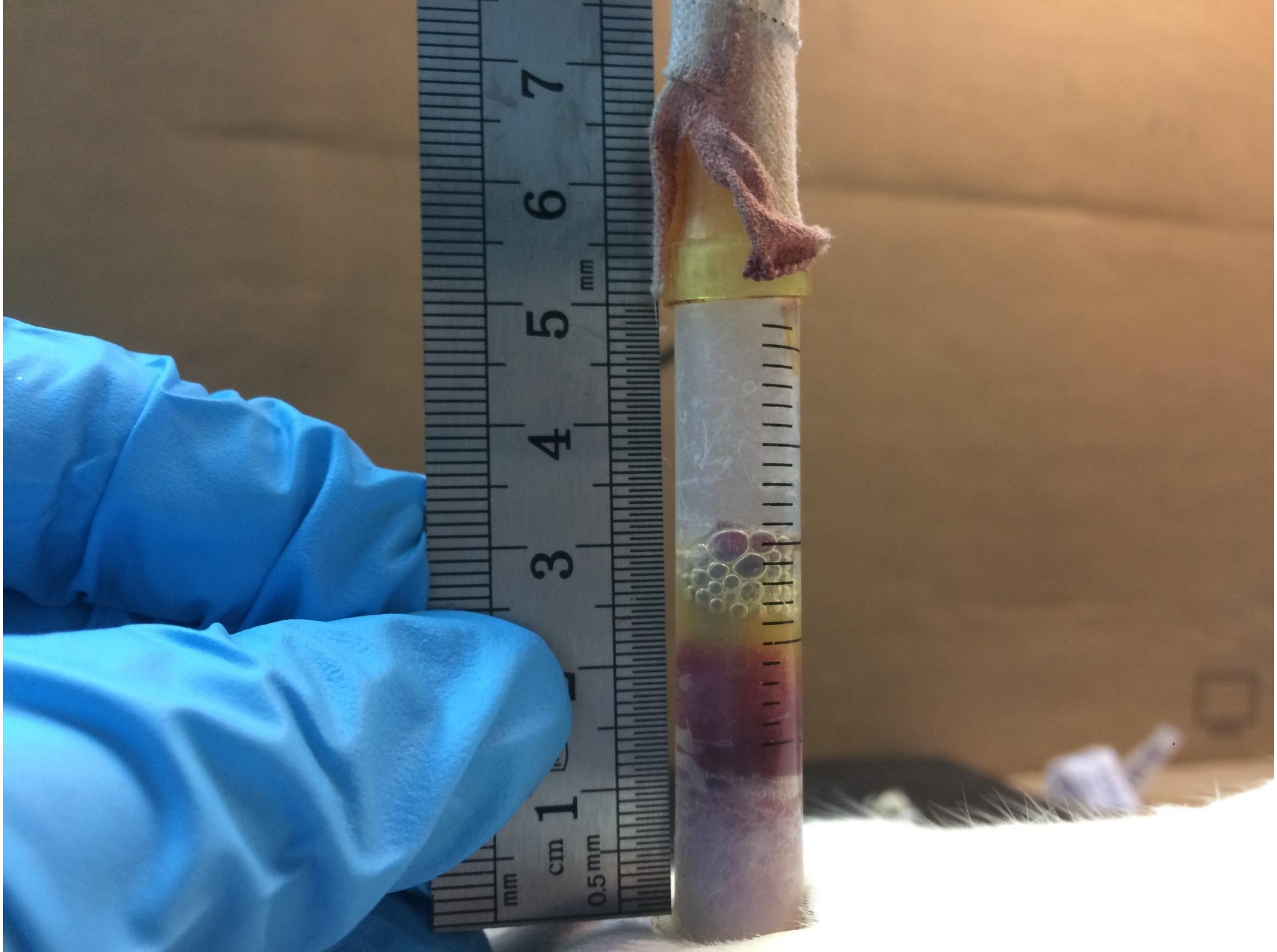

Anti-lox 1

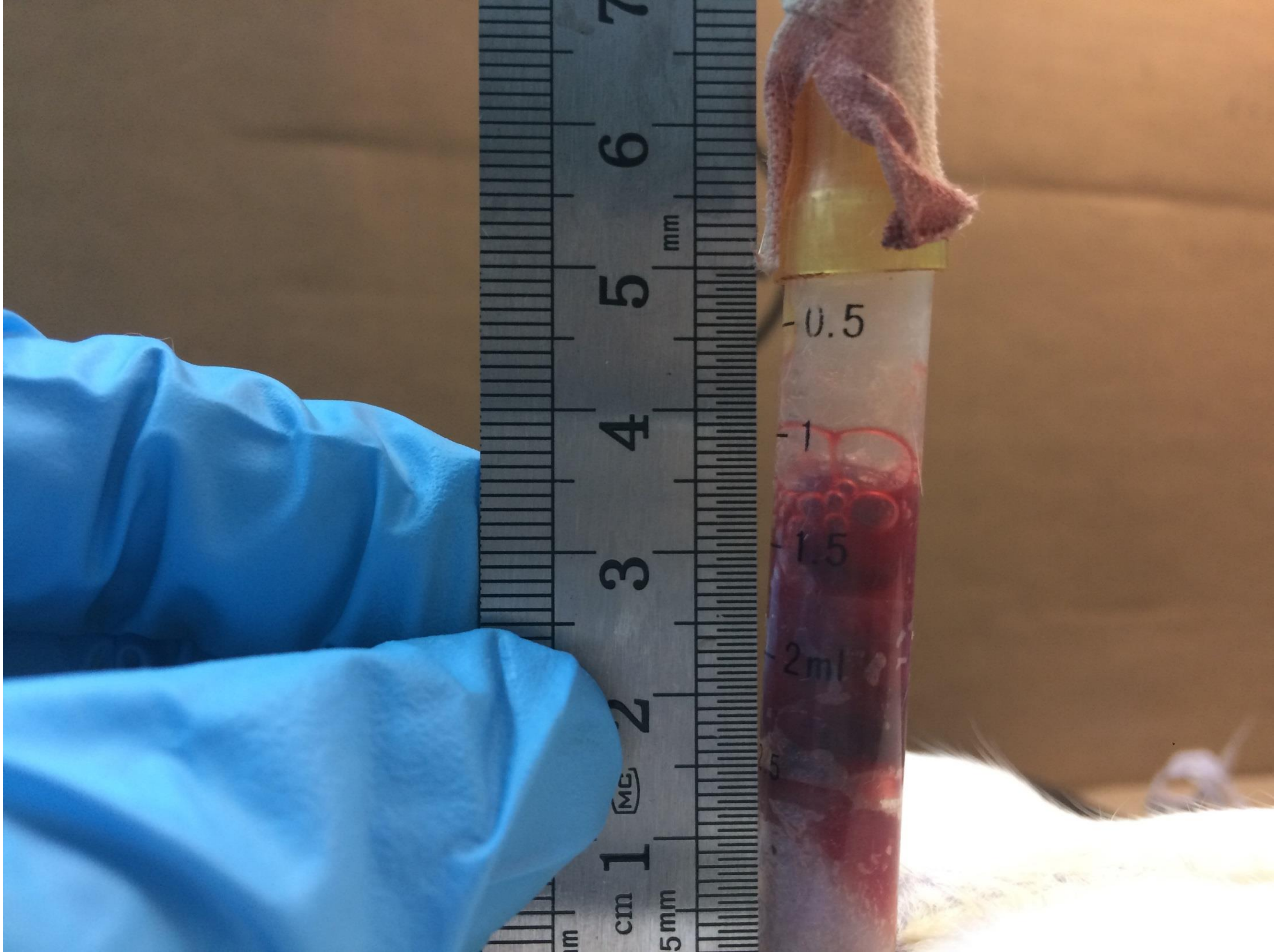

Anti-lox 2

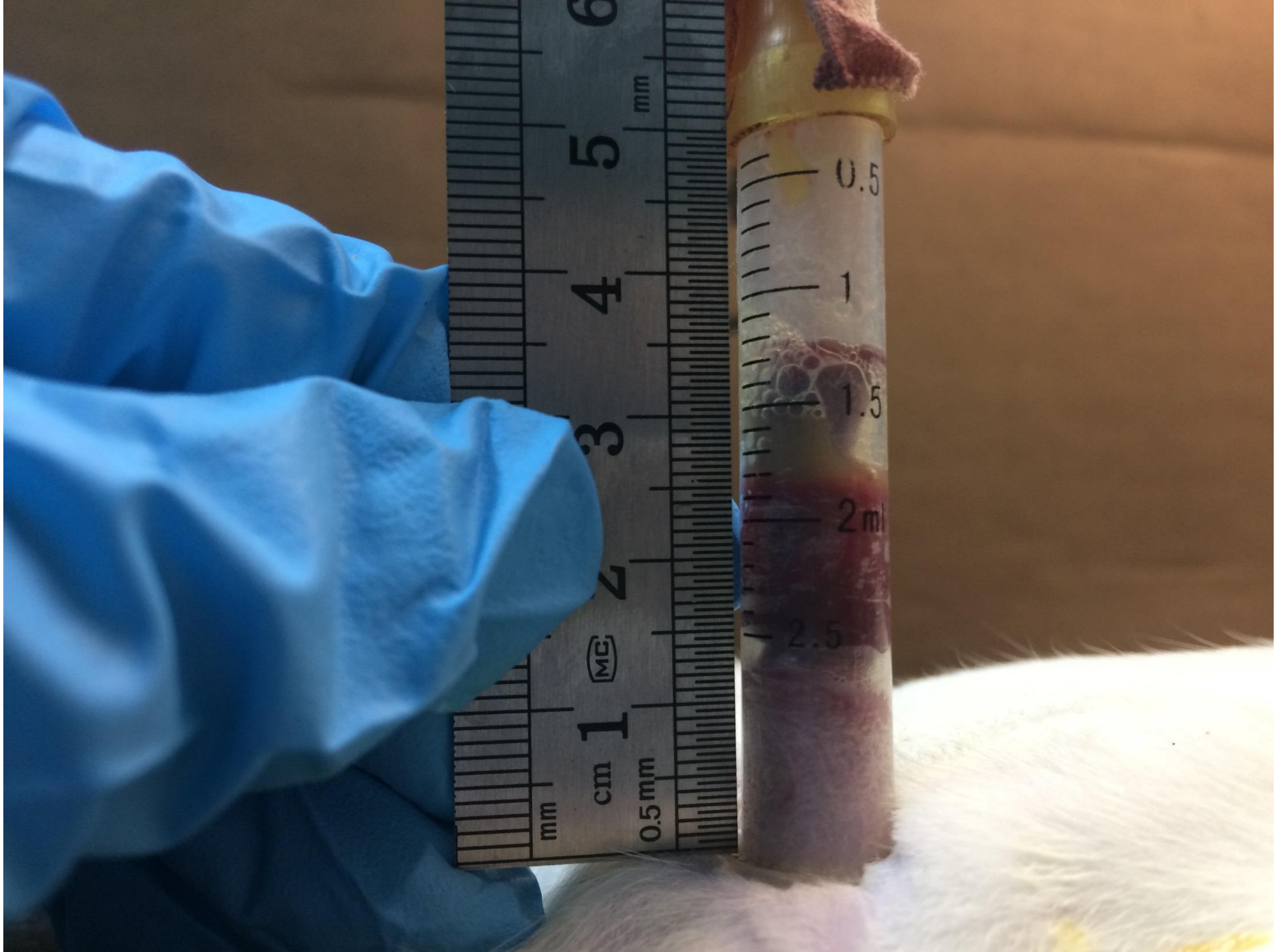

Anti-lox 3

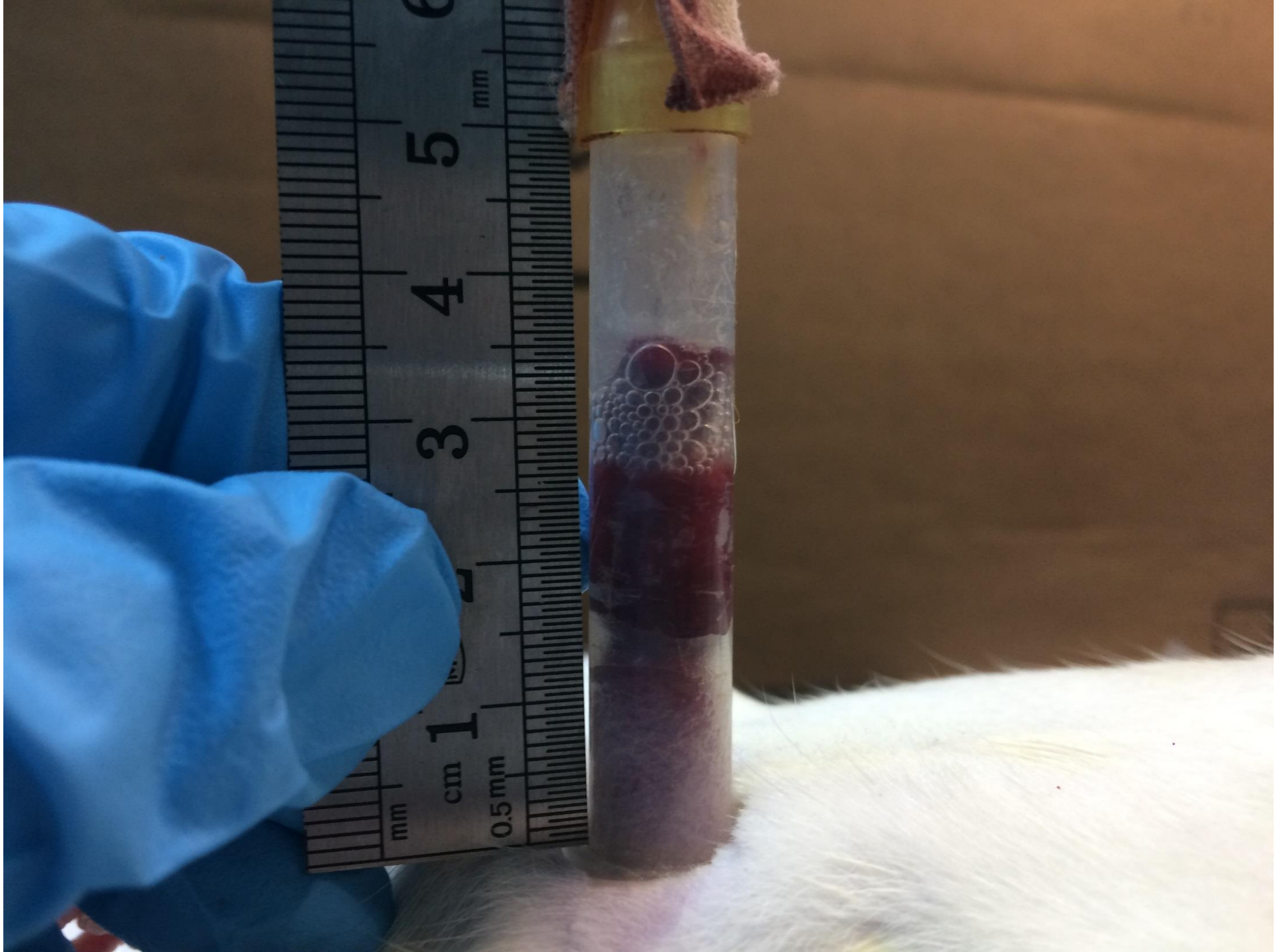

Anti-lox 4

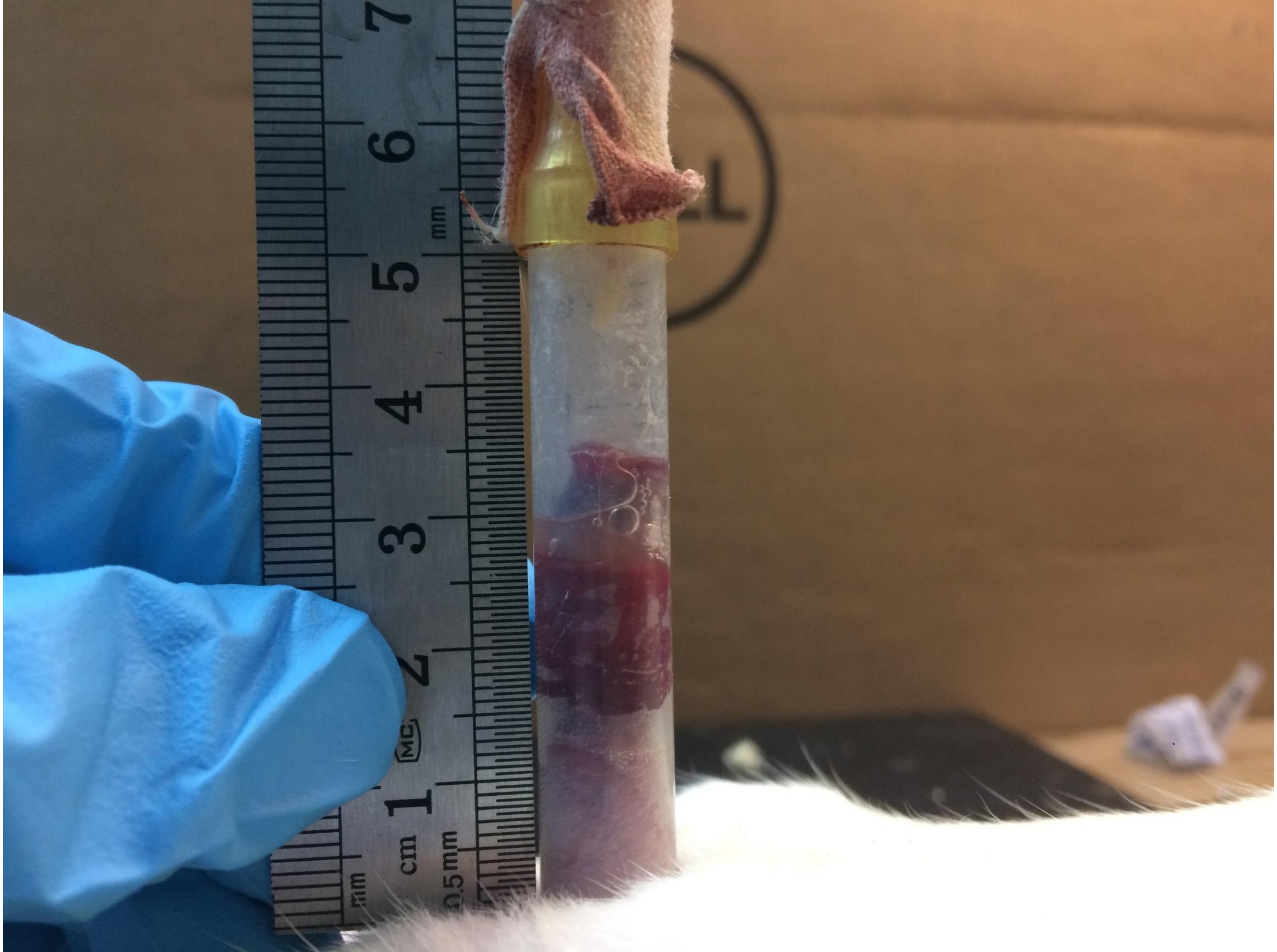

Anti-lox 5

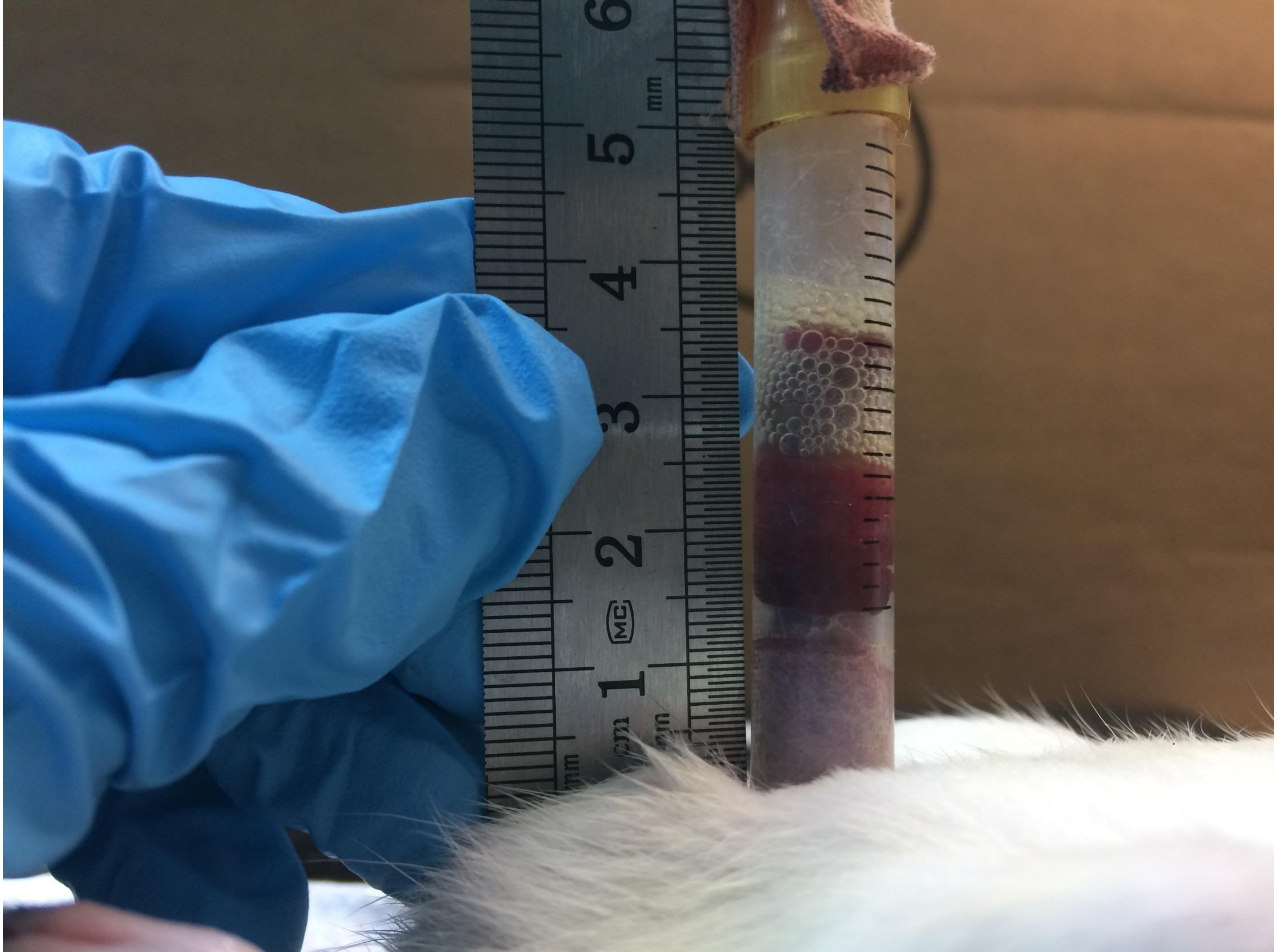

Anti-lox 6

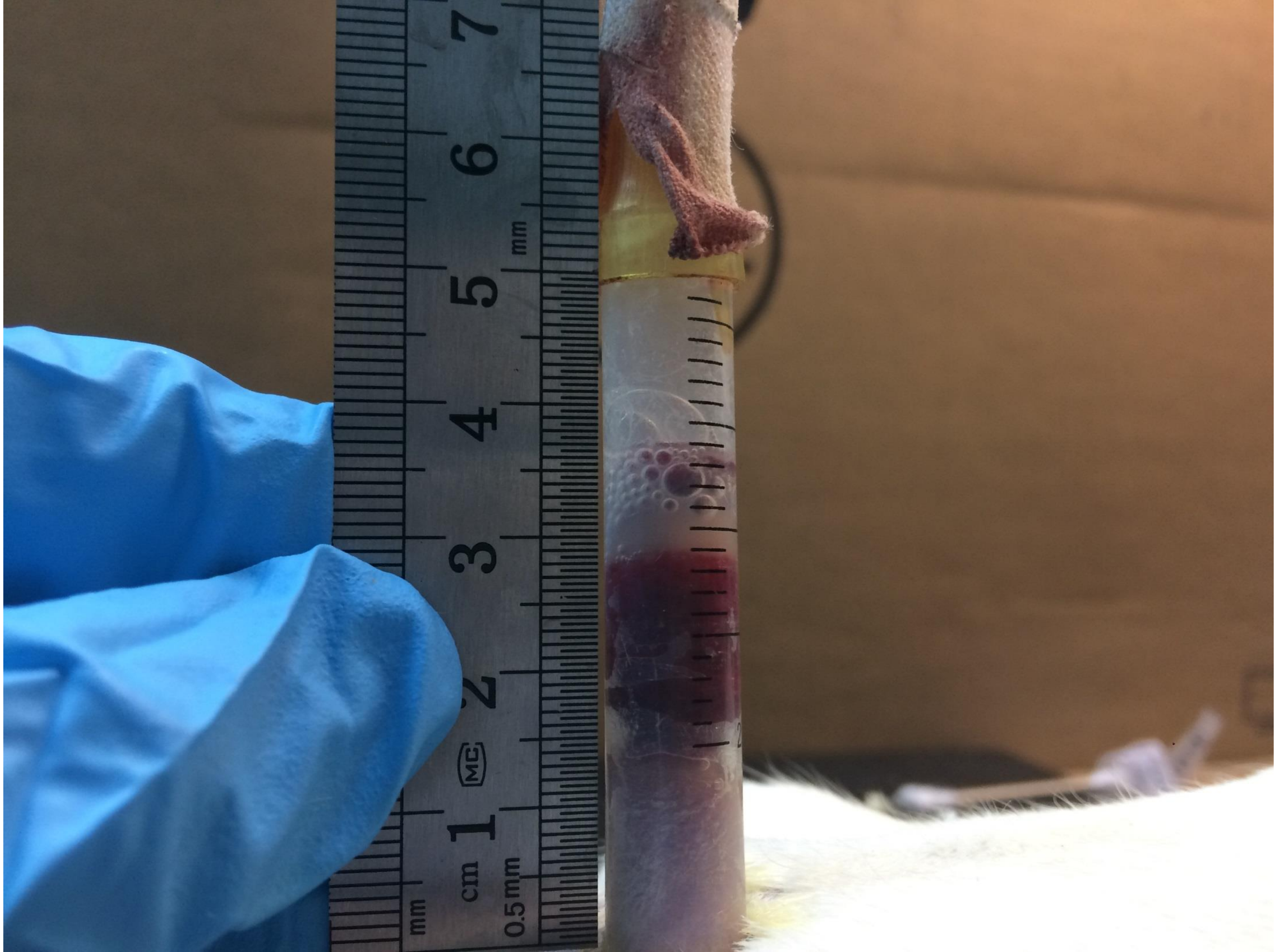

HCG 1

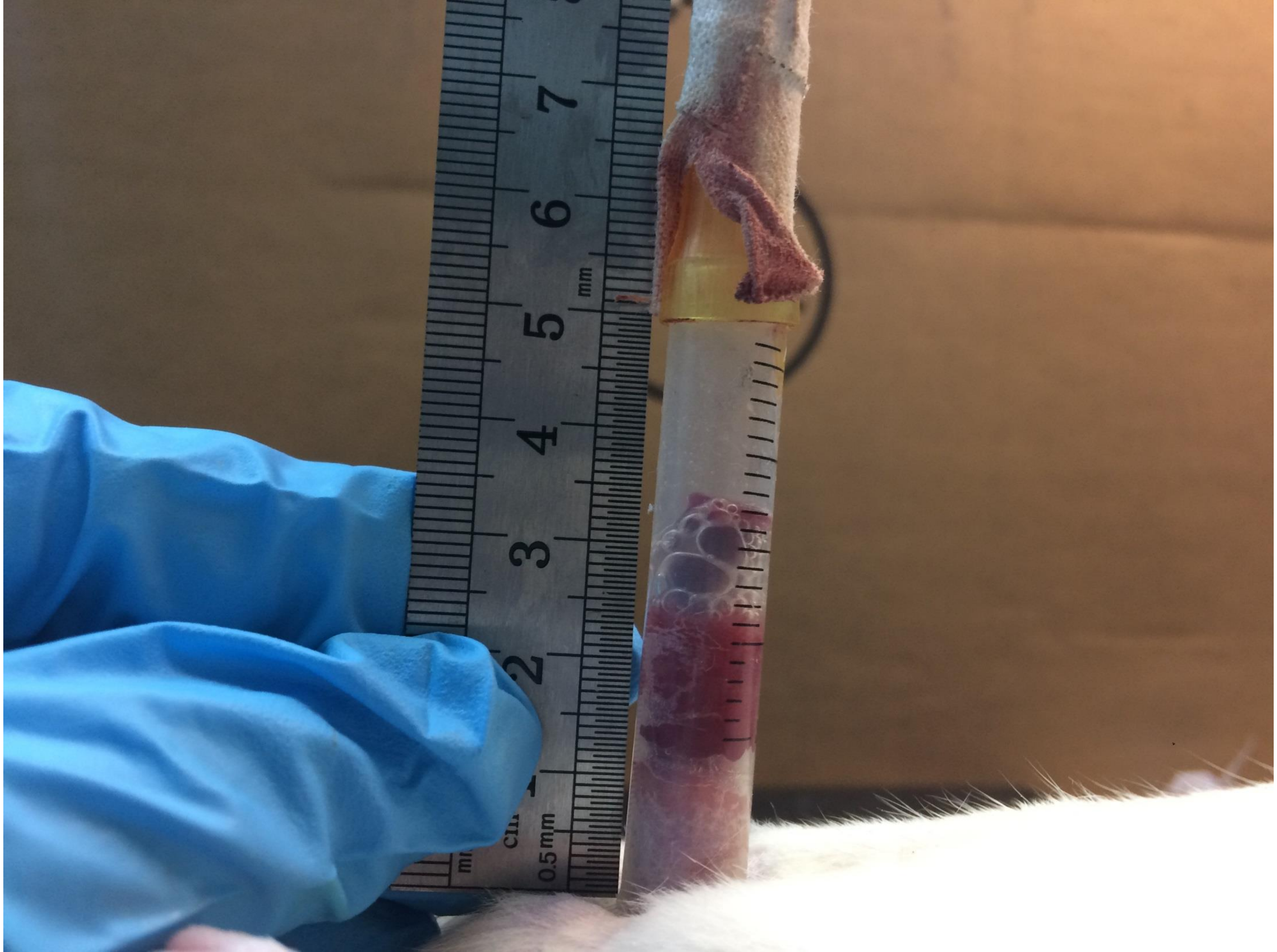

HCG 2

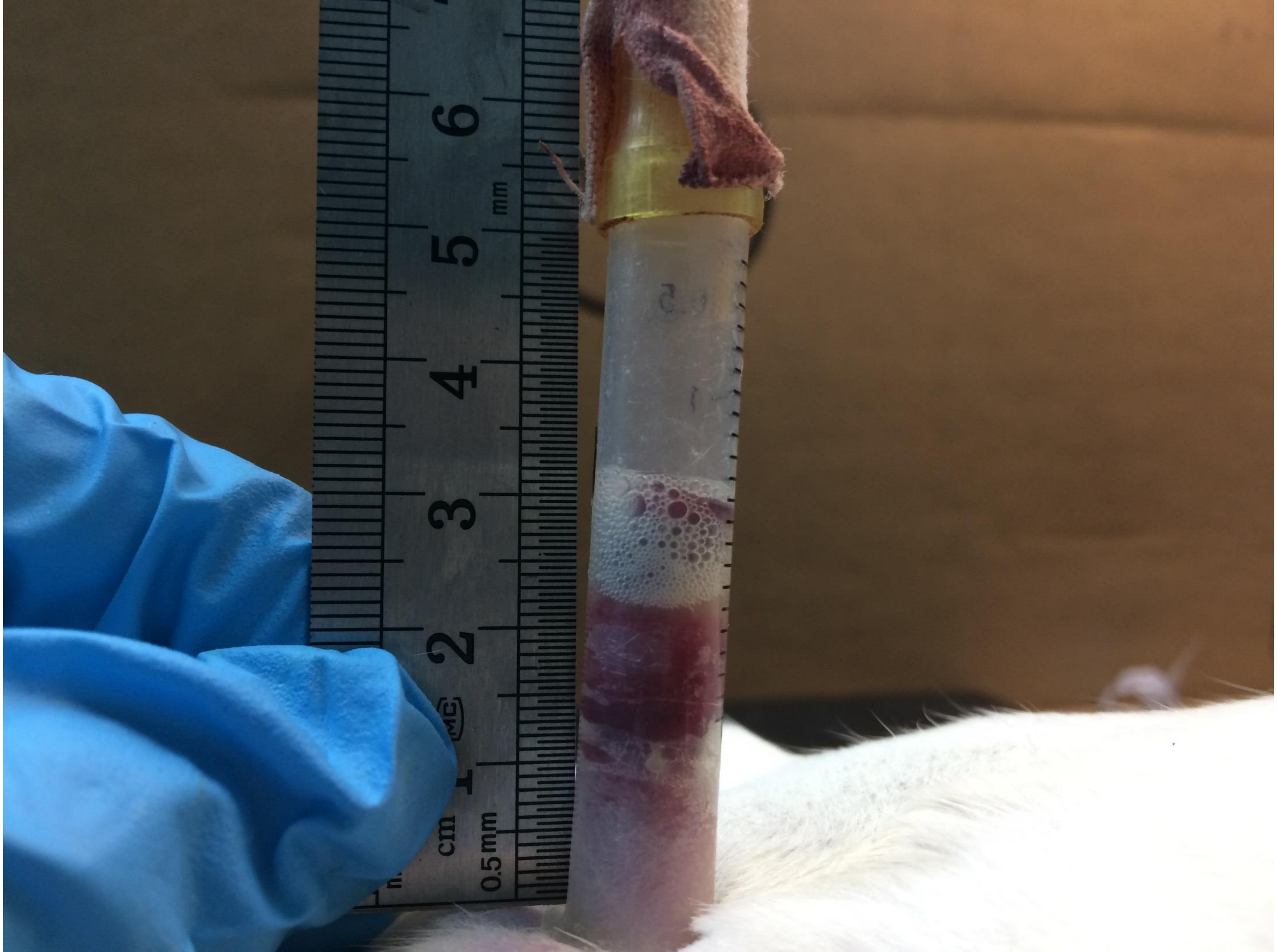

HCG 3

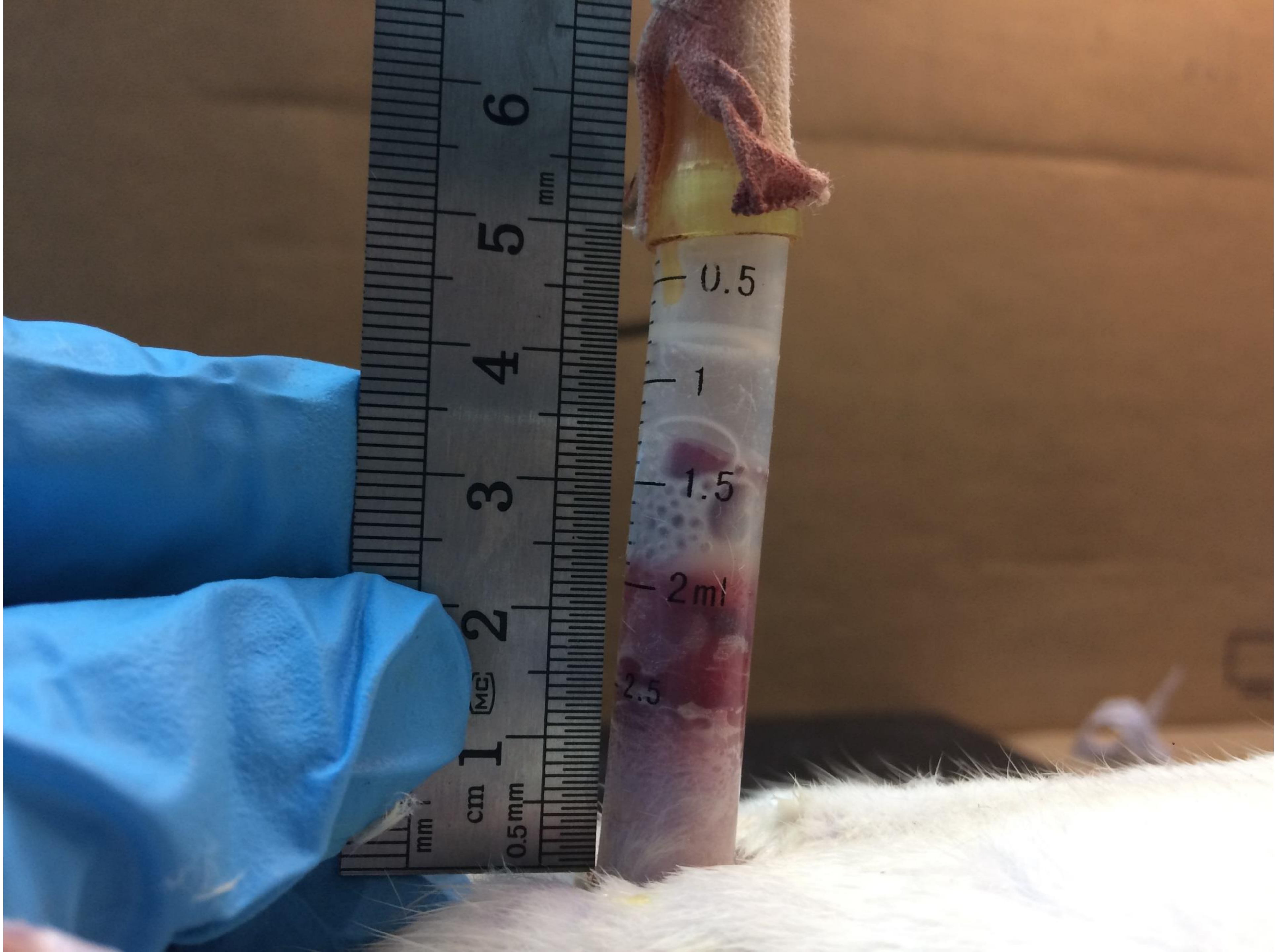

HCG 4

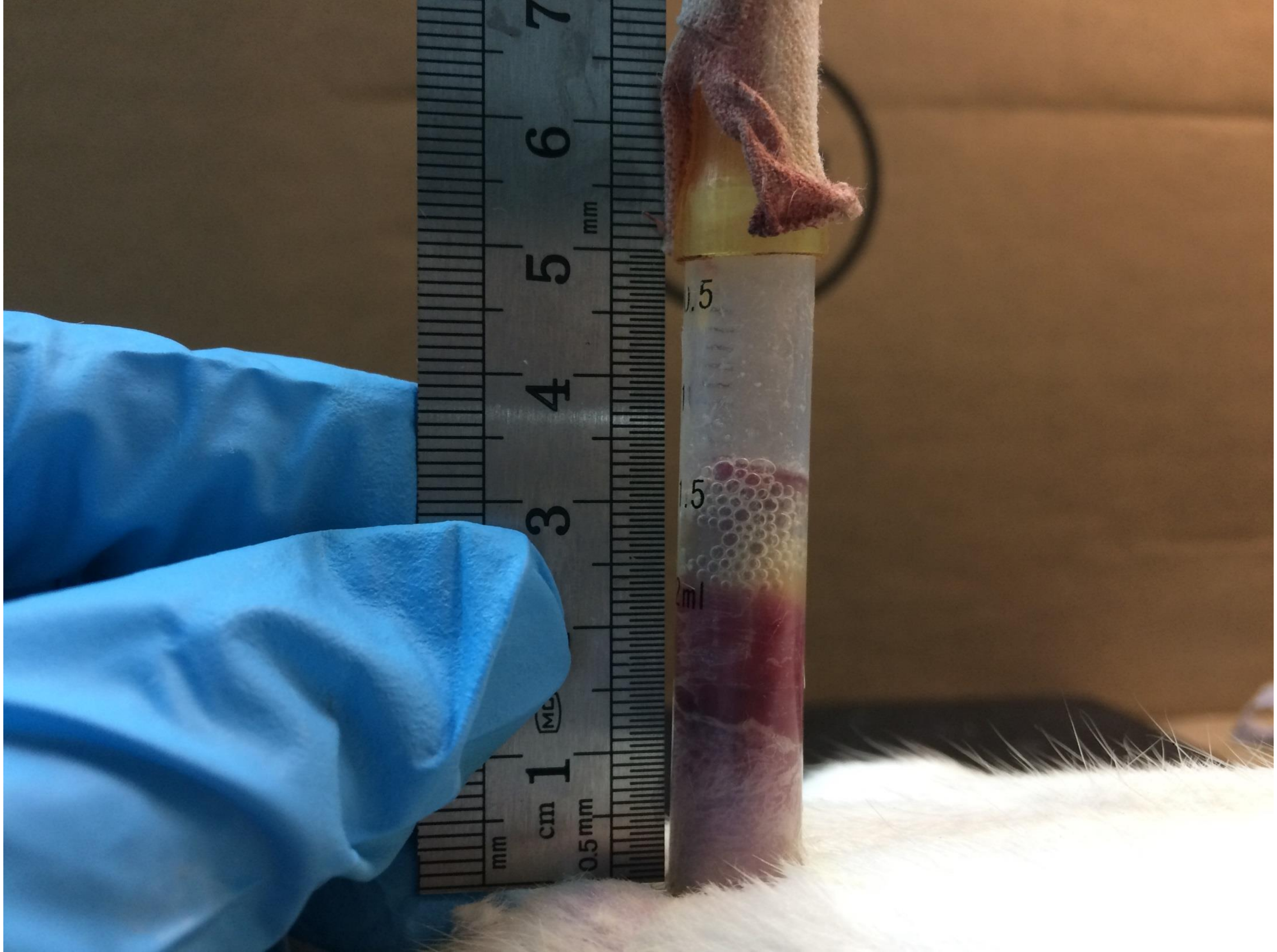

HCG 5

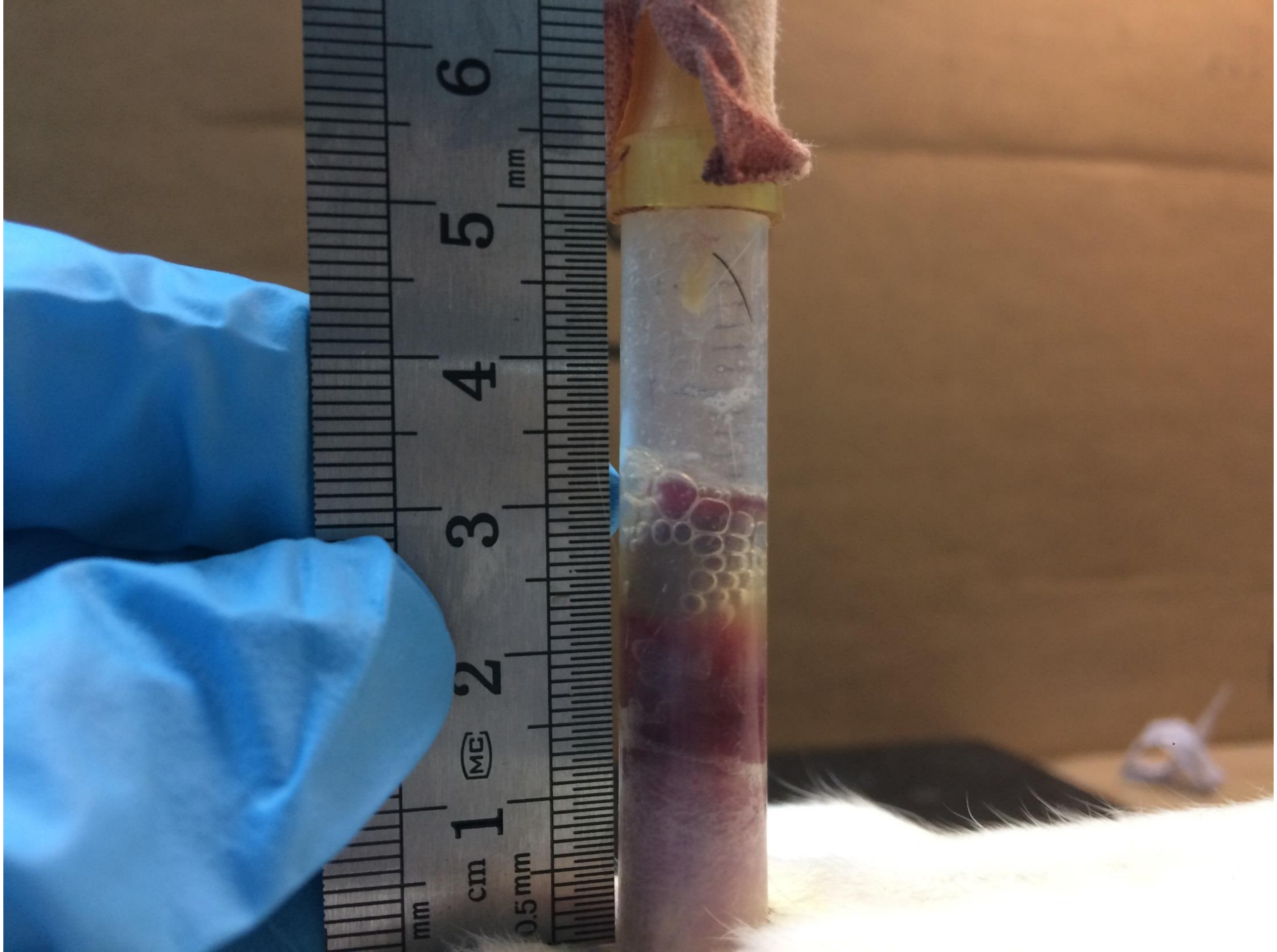

HCG 6

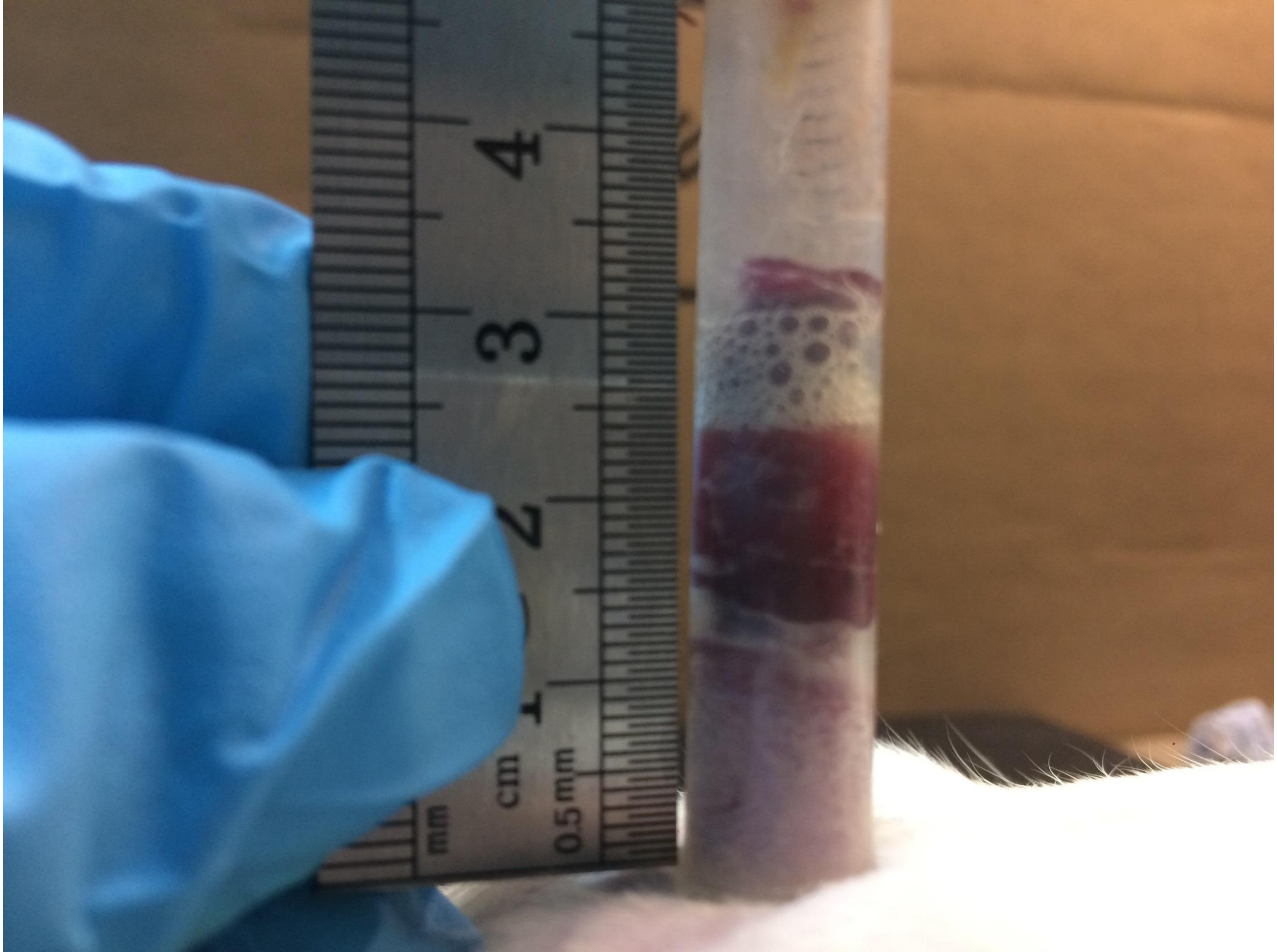

-300 1

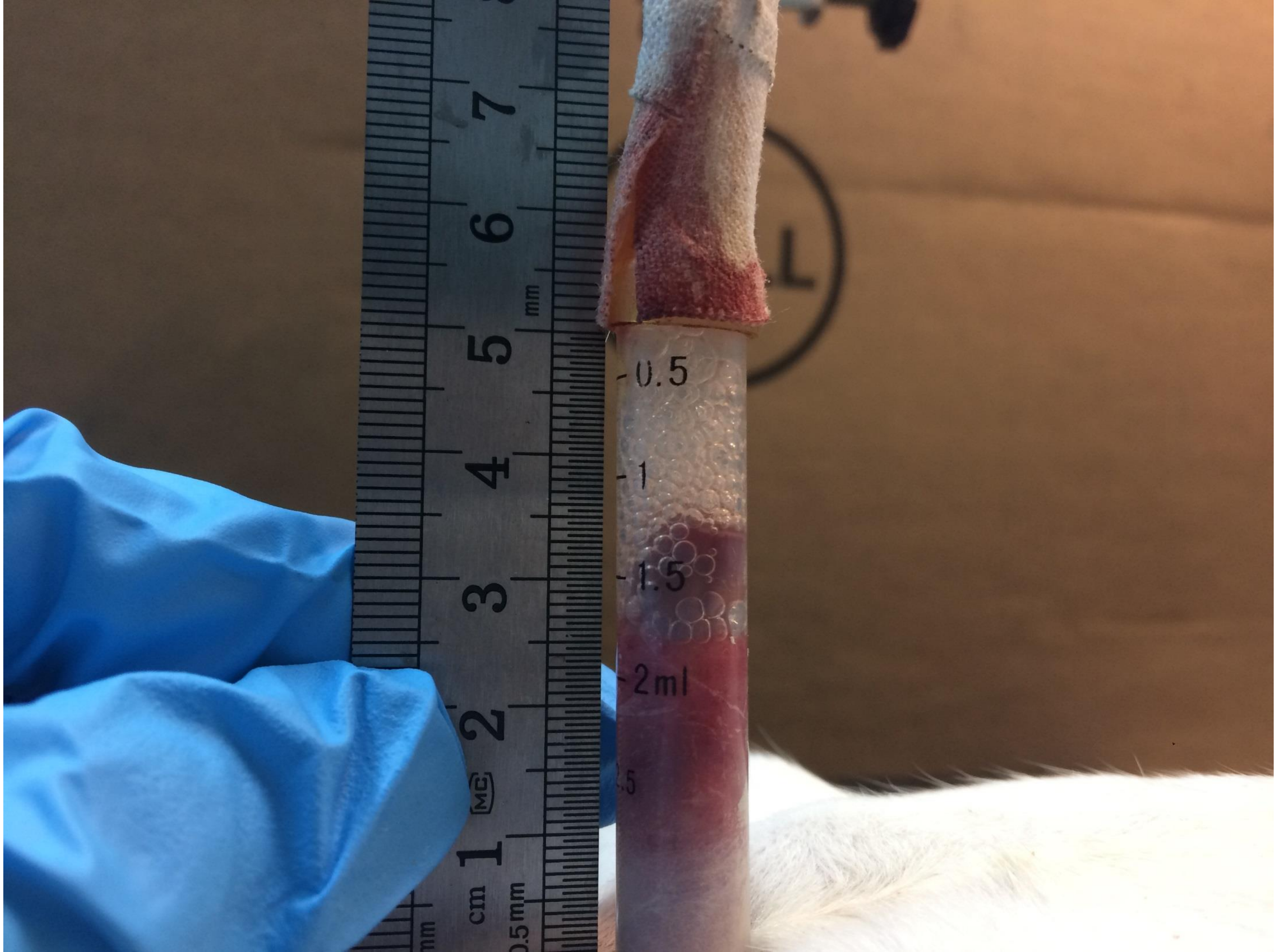

-300 2

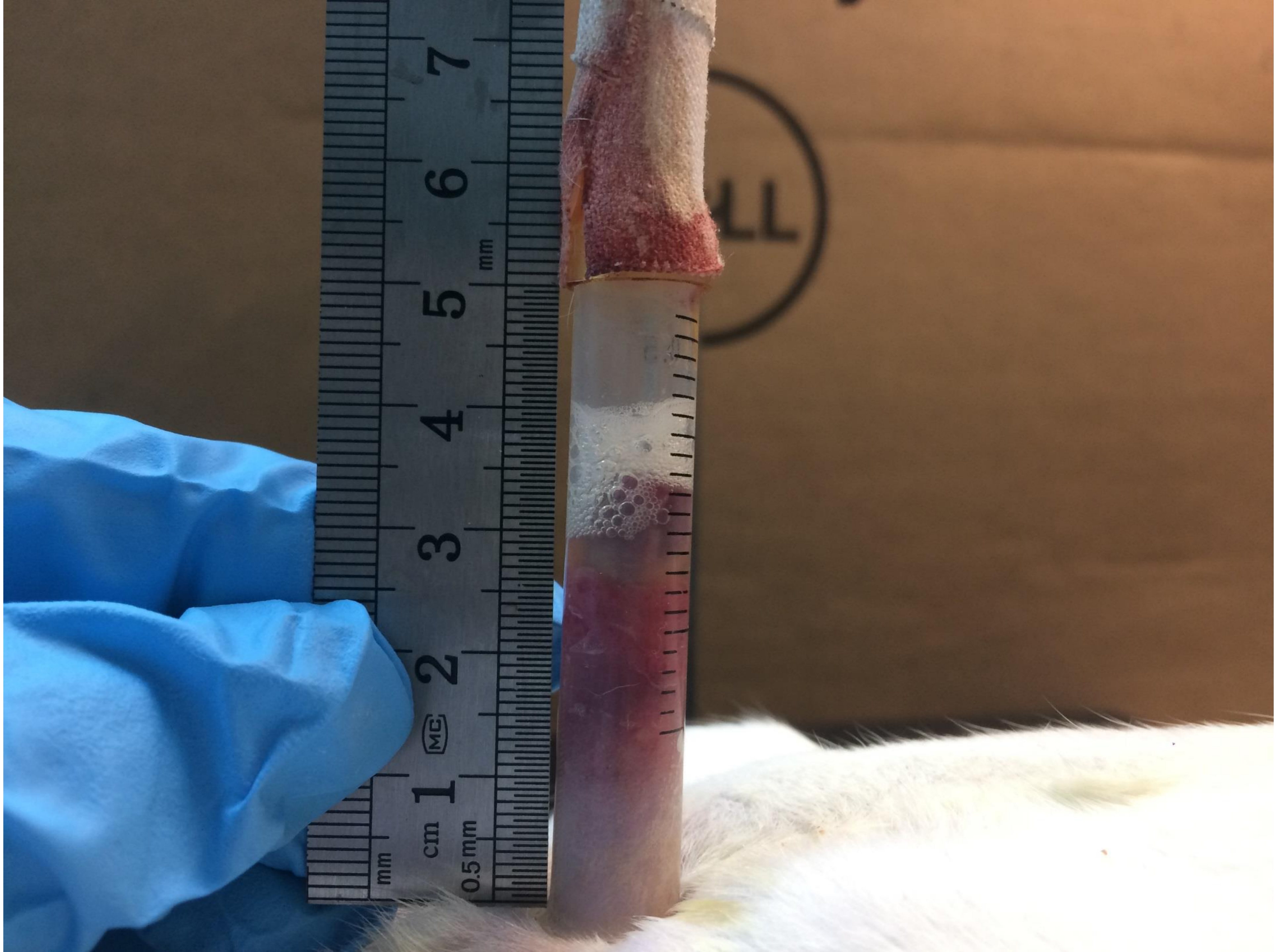

-300 3

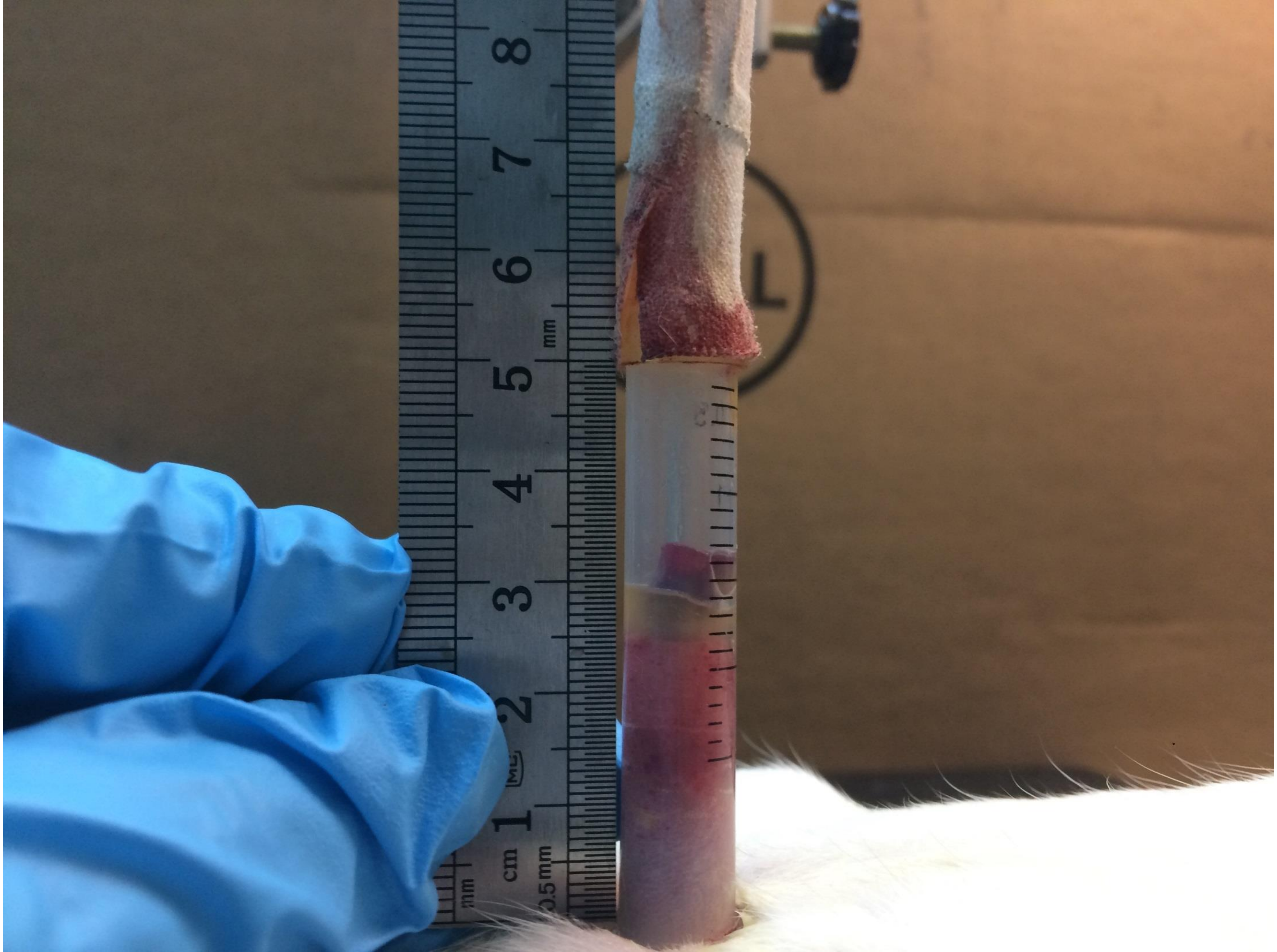

-300 4

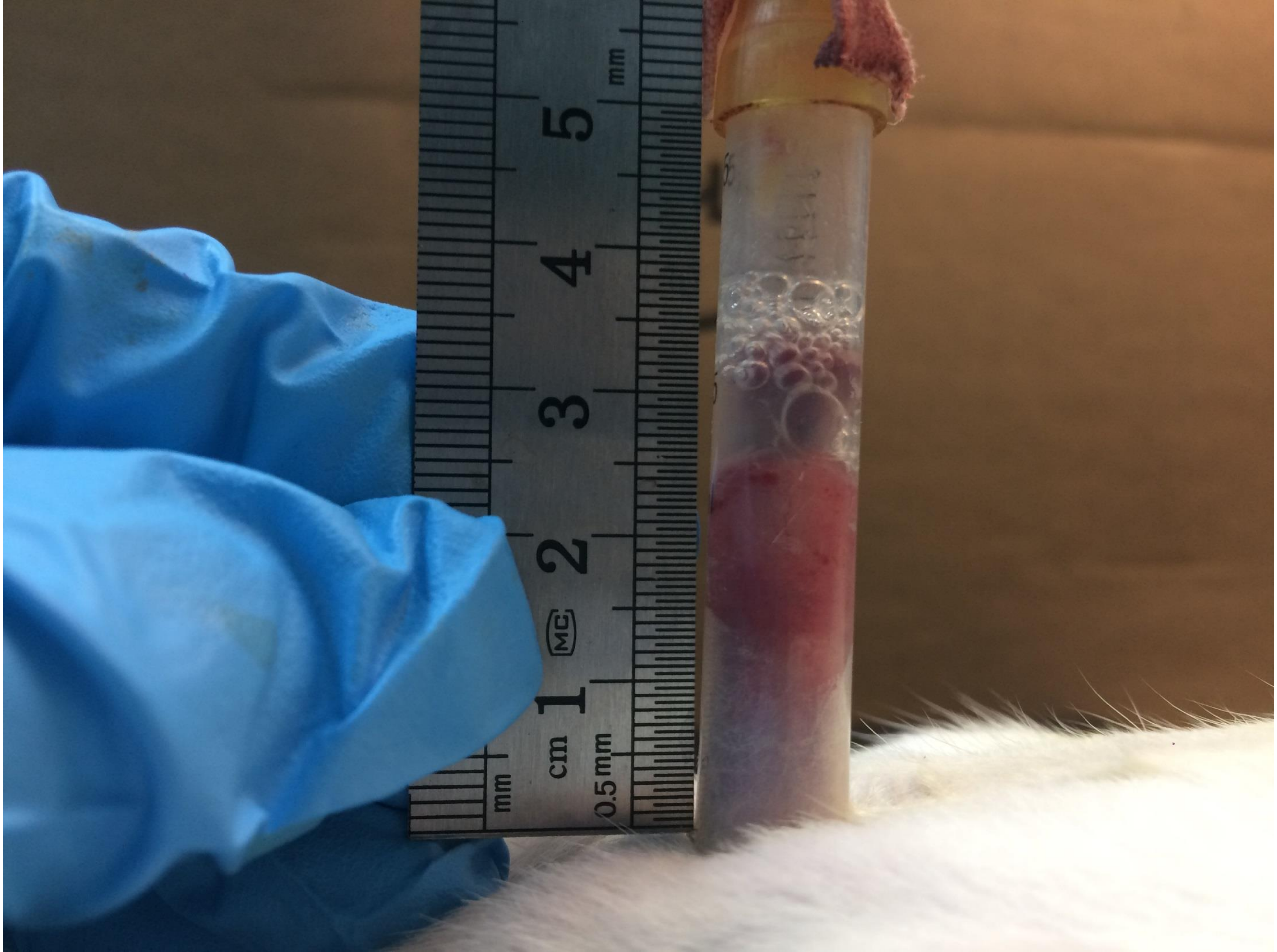

-300 5

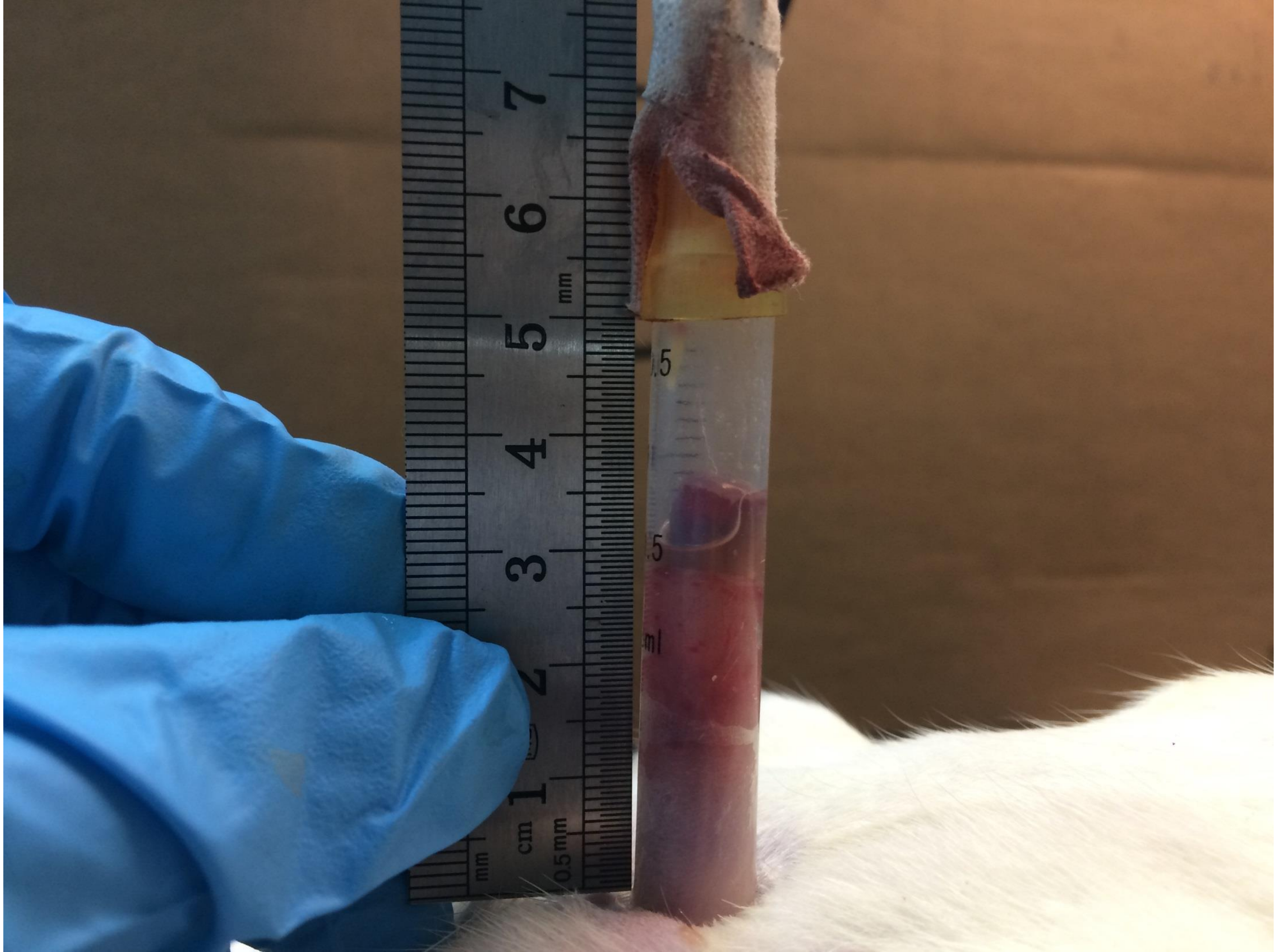

-300 6

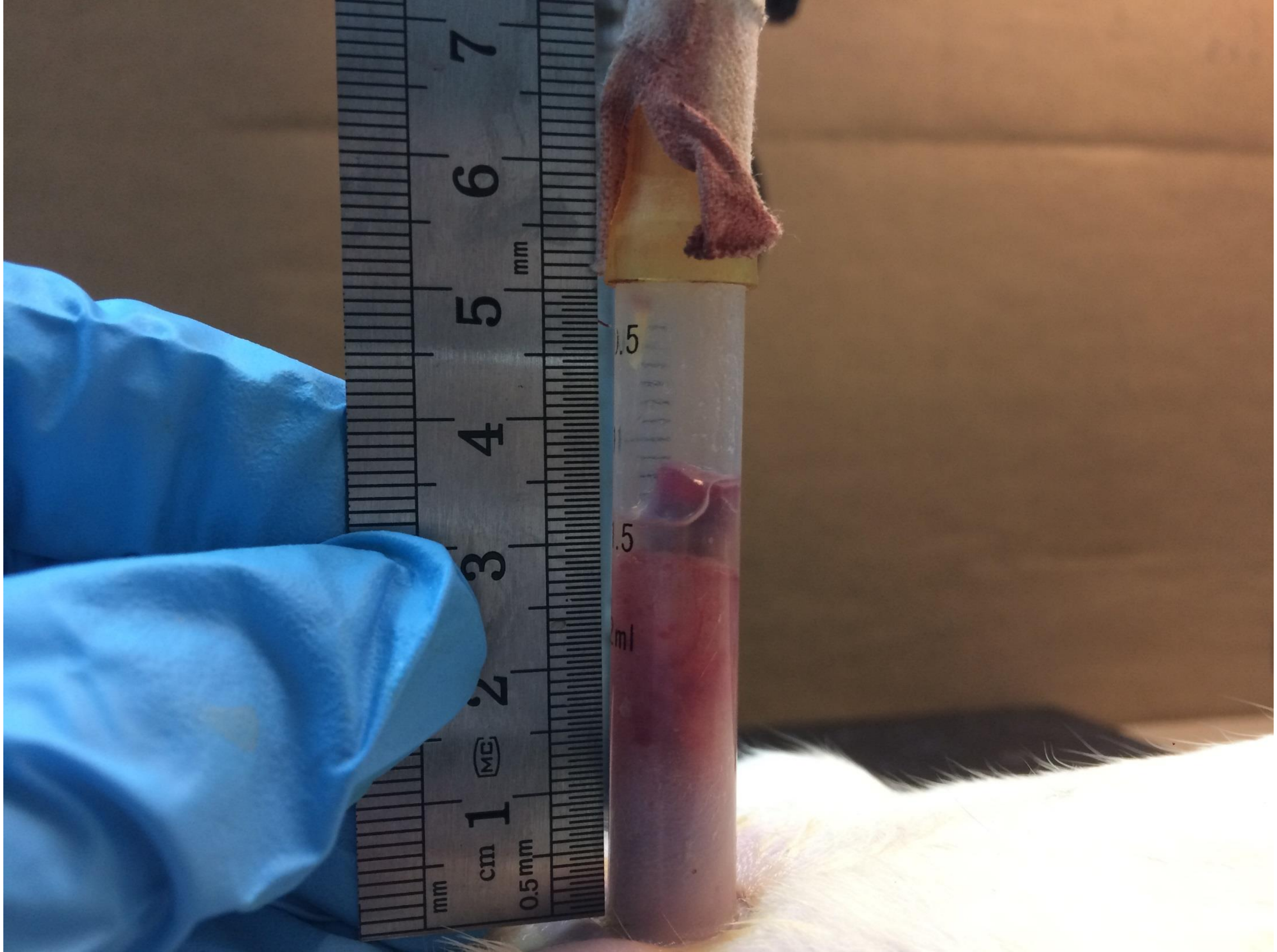

-300+Anti-LOX 1

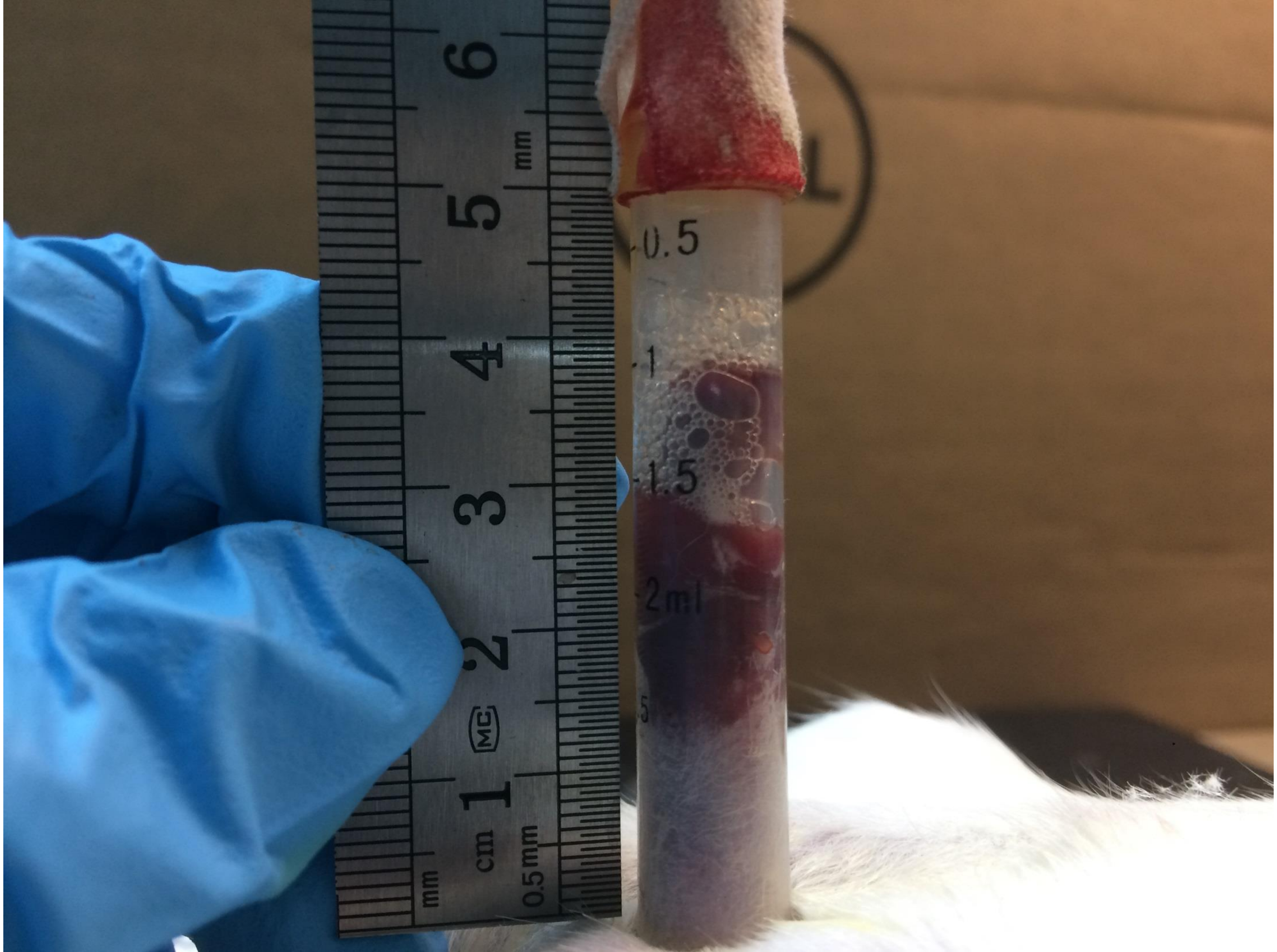

-300+Anti-LOX 2

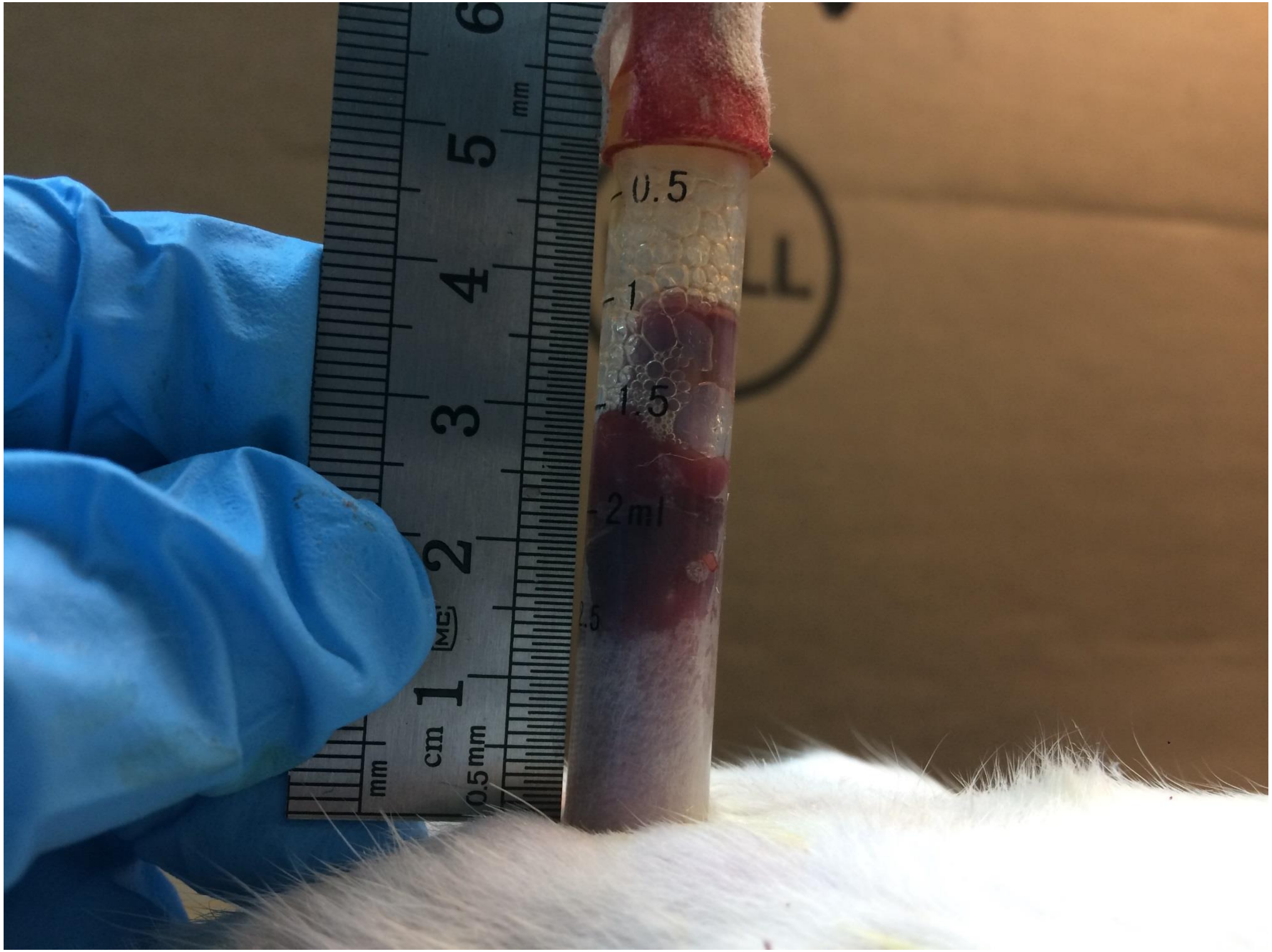

-300+Anti-LOX 3

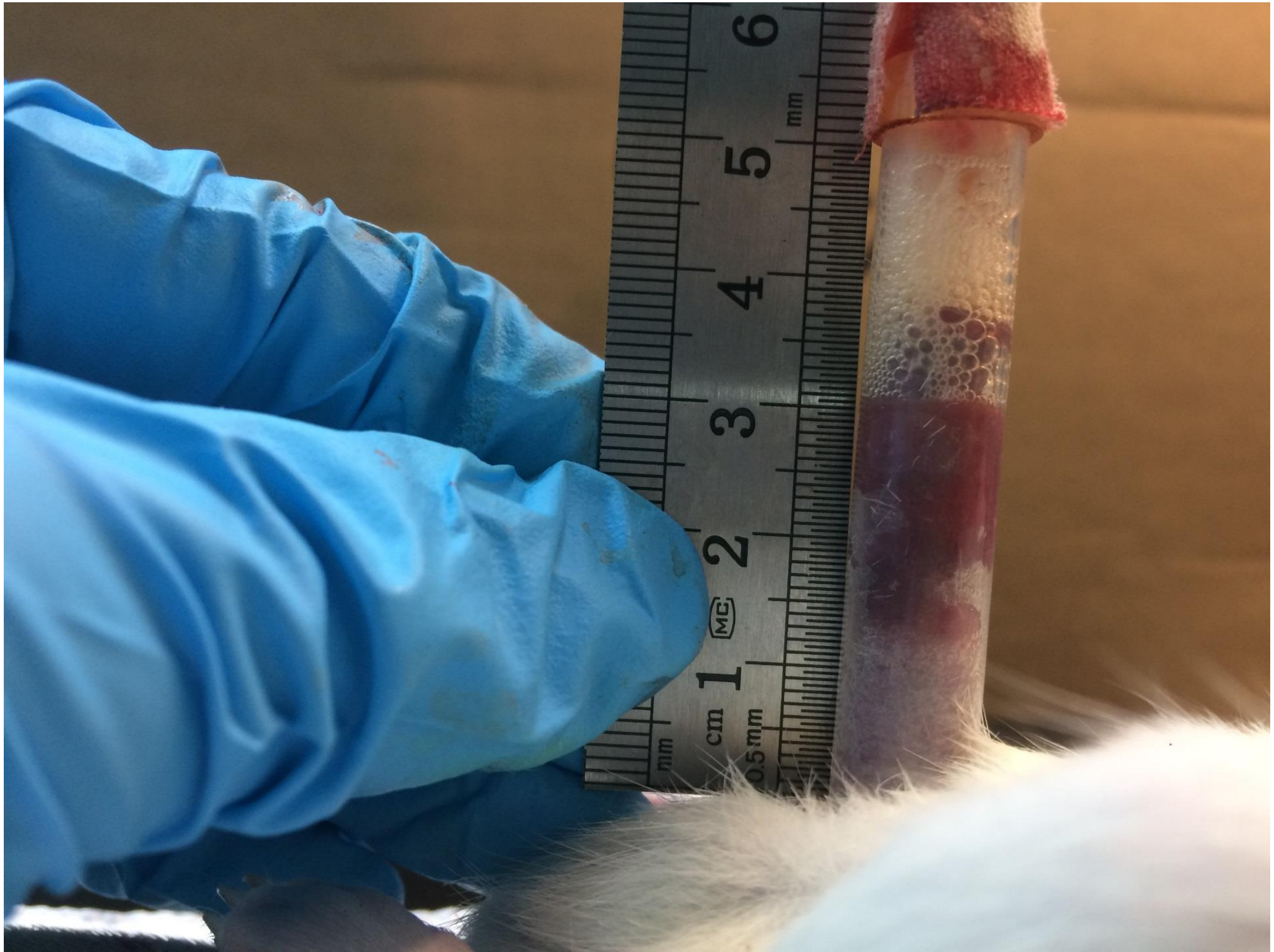

-300+Anti-LOX 4

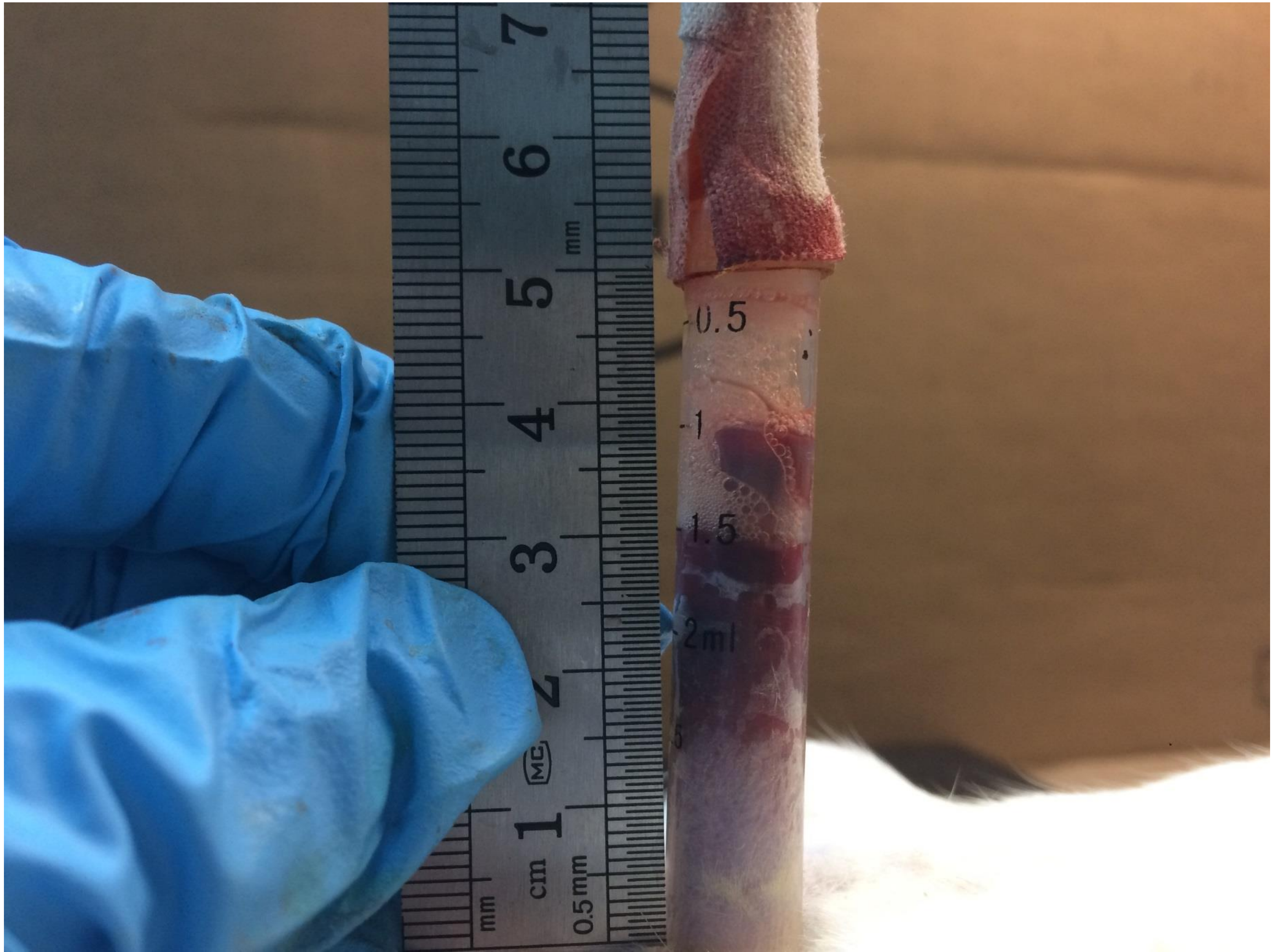

-300+Anti-LOX 5

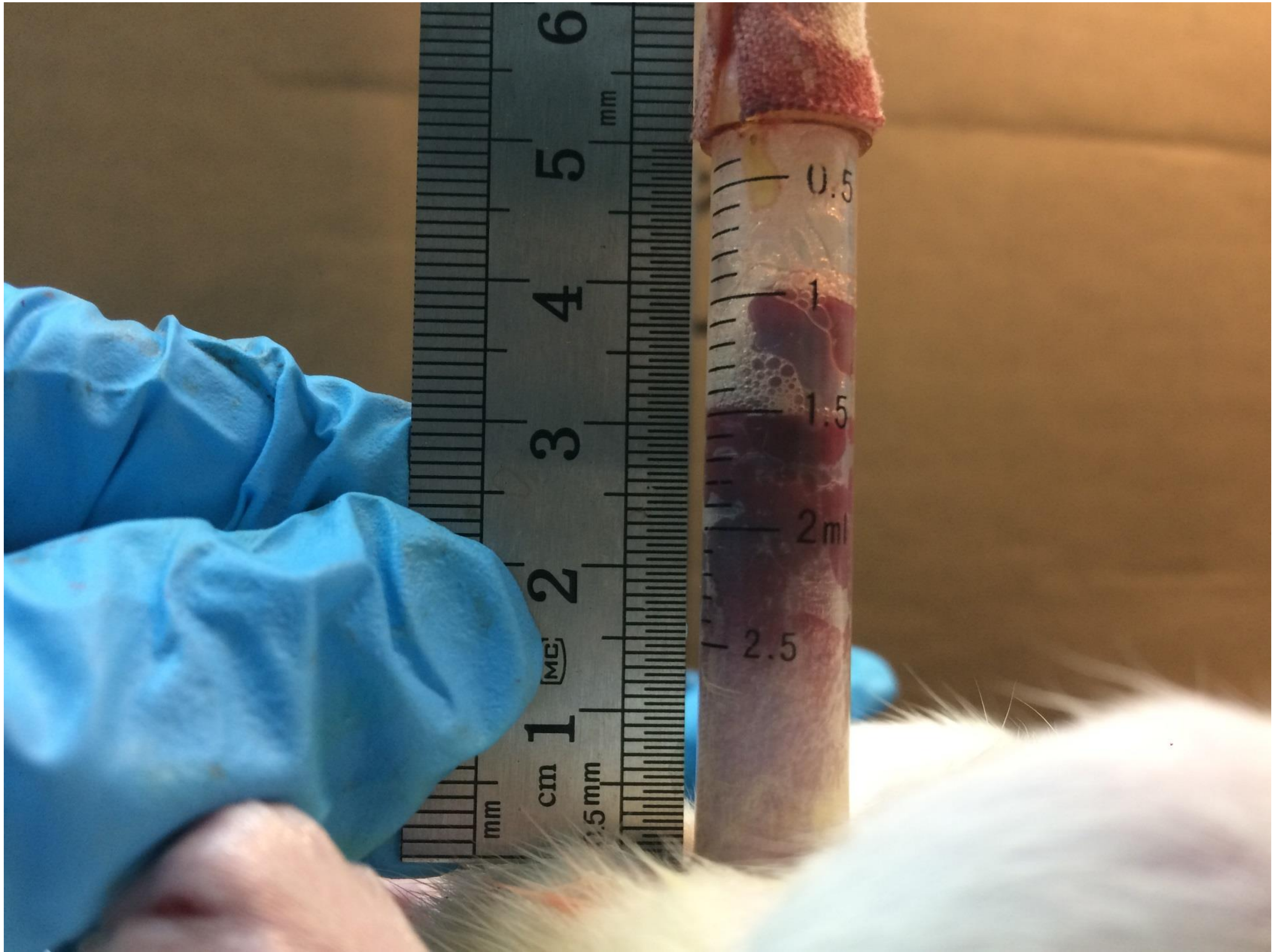

-300+Anti-LOX 6

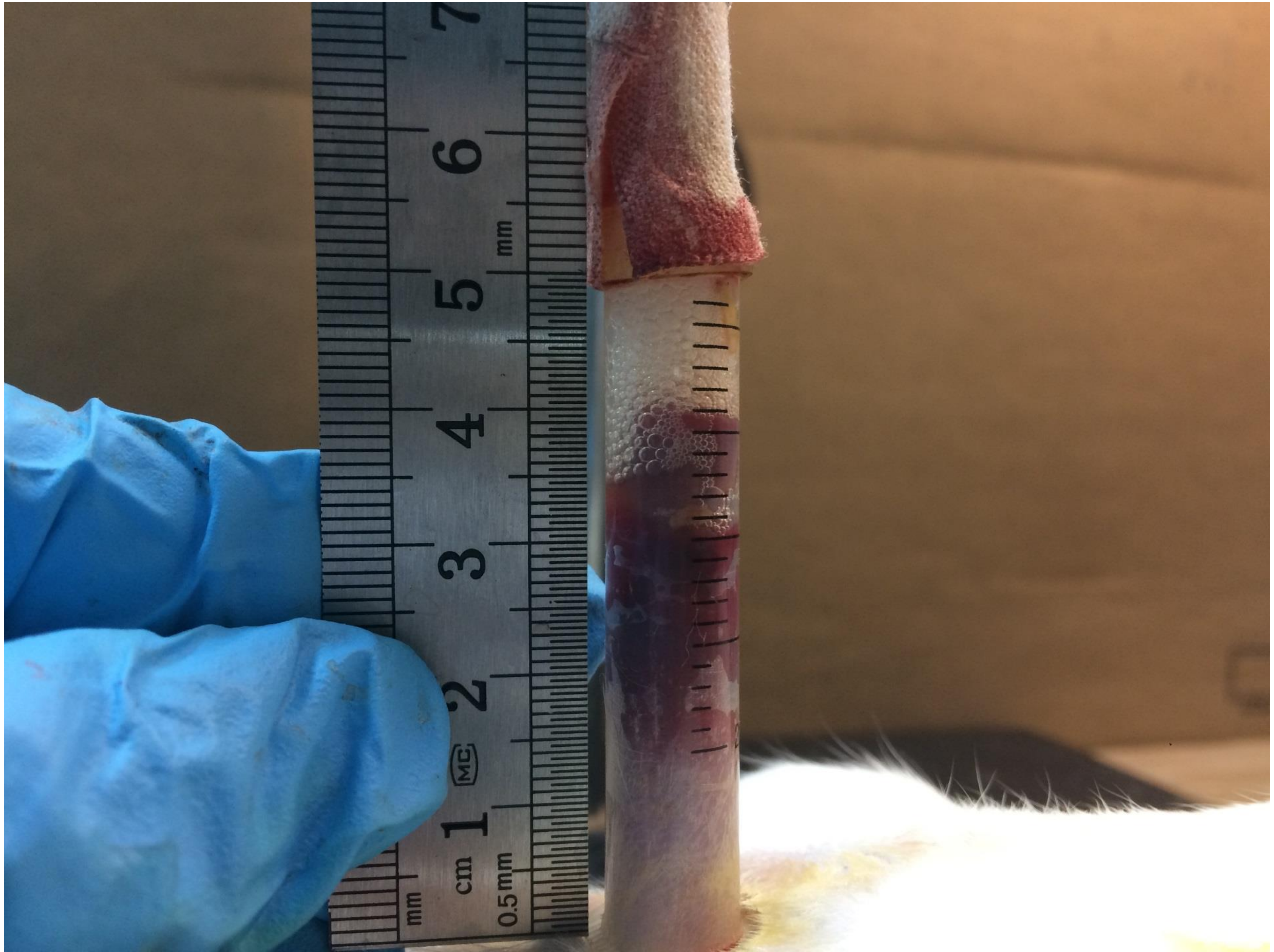

-300+HCG 1

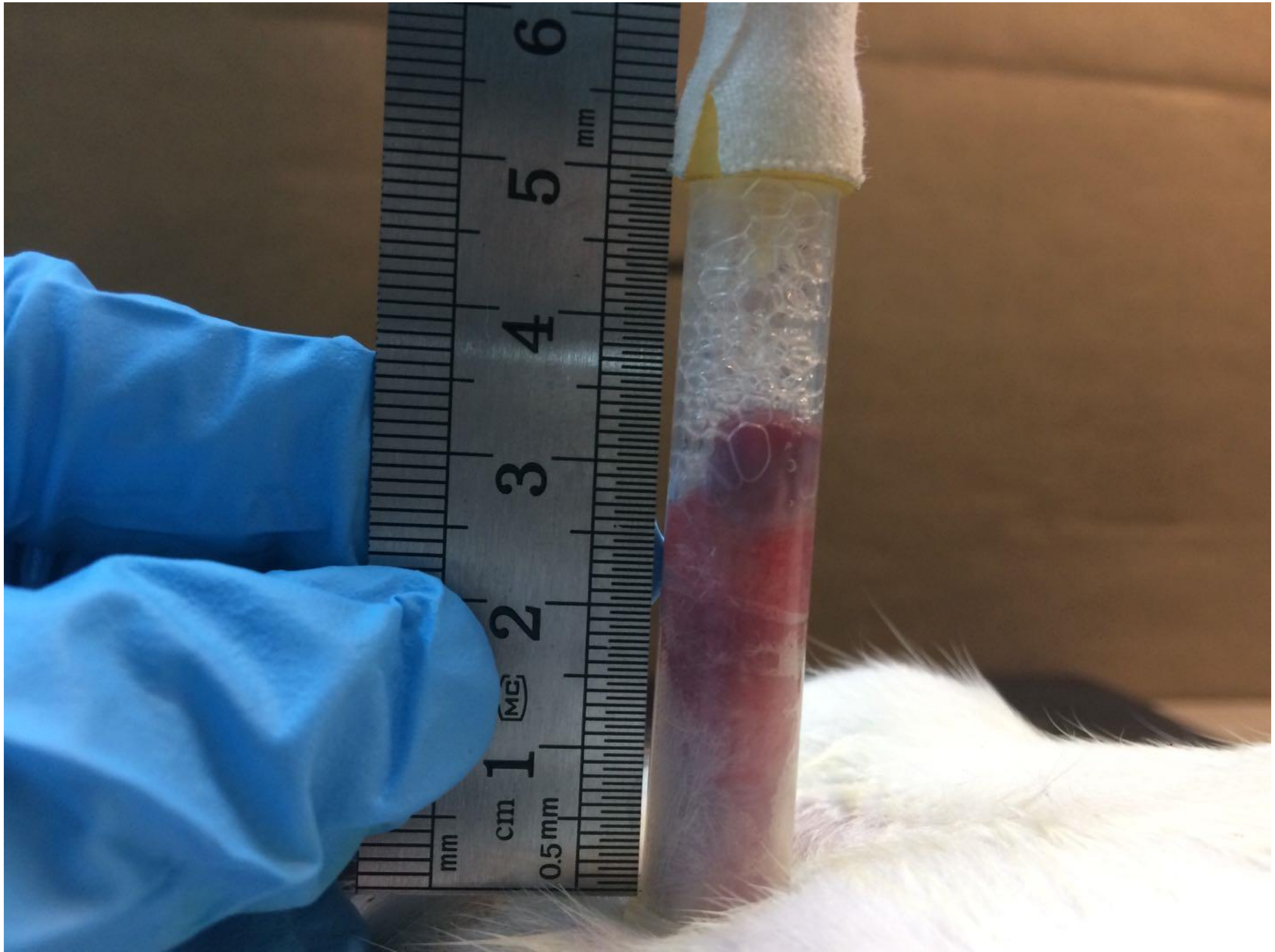

-300+HCG 2

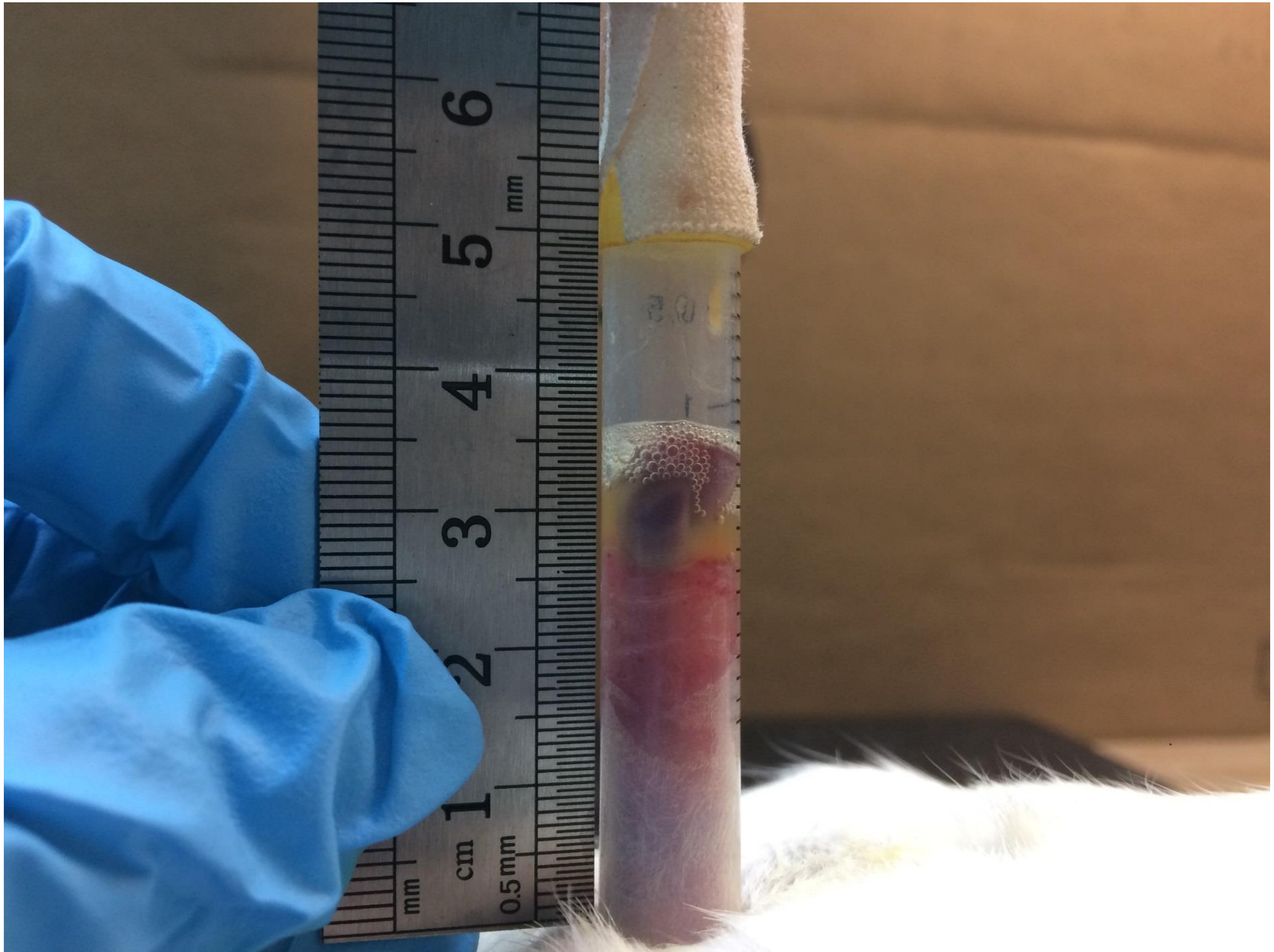

-300+HCG 3

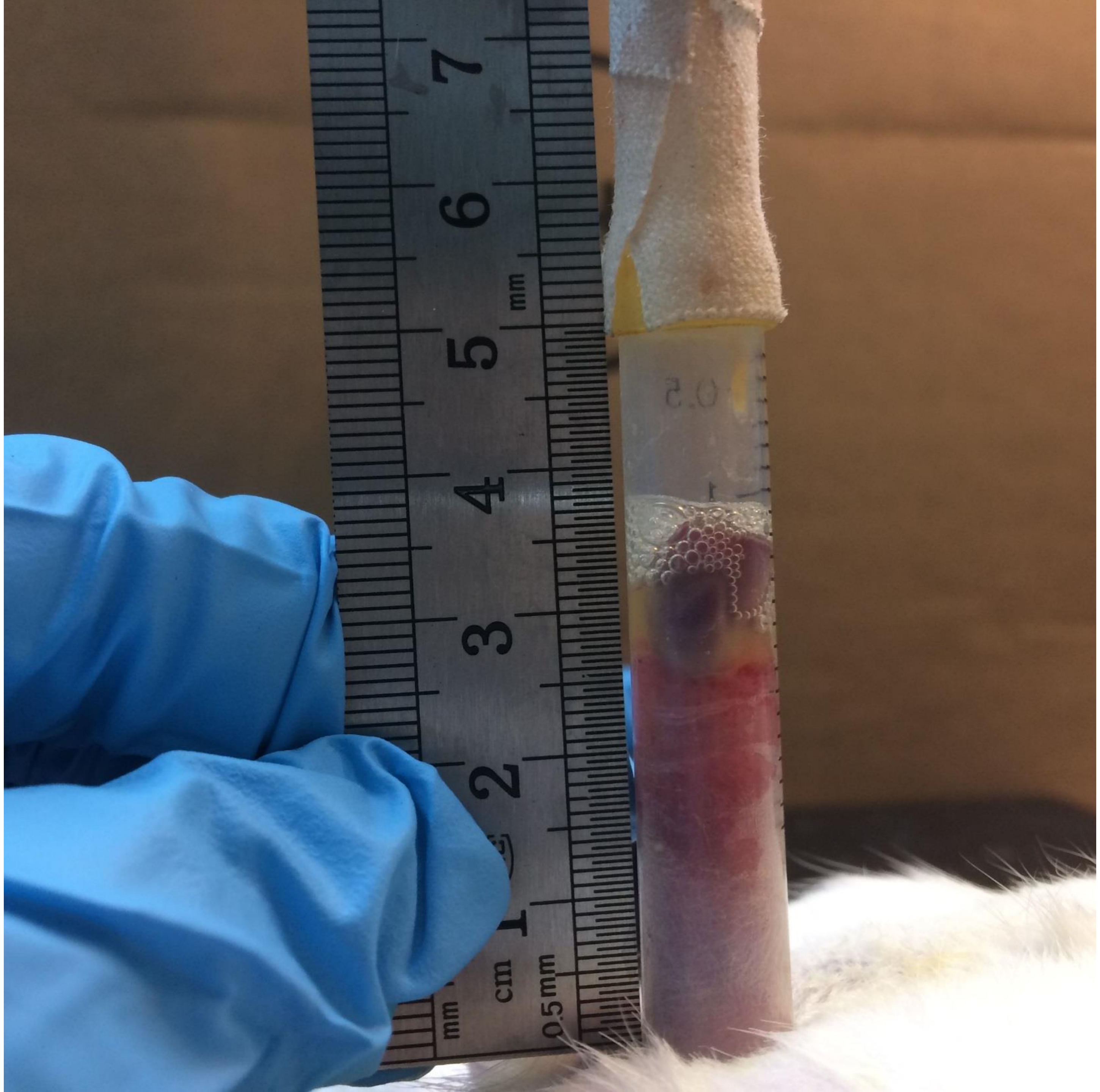

-300+HCG 4

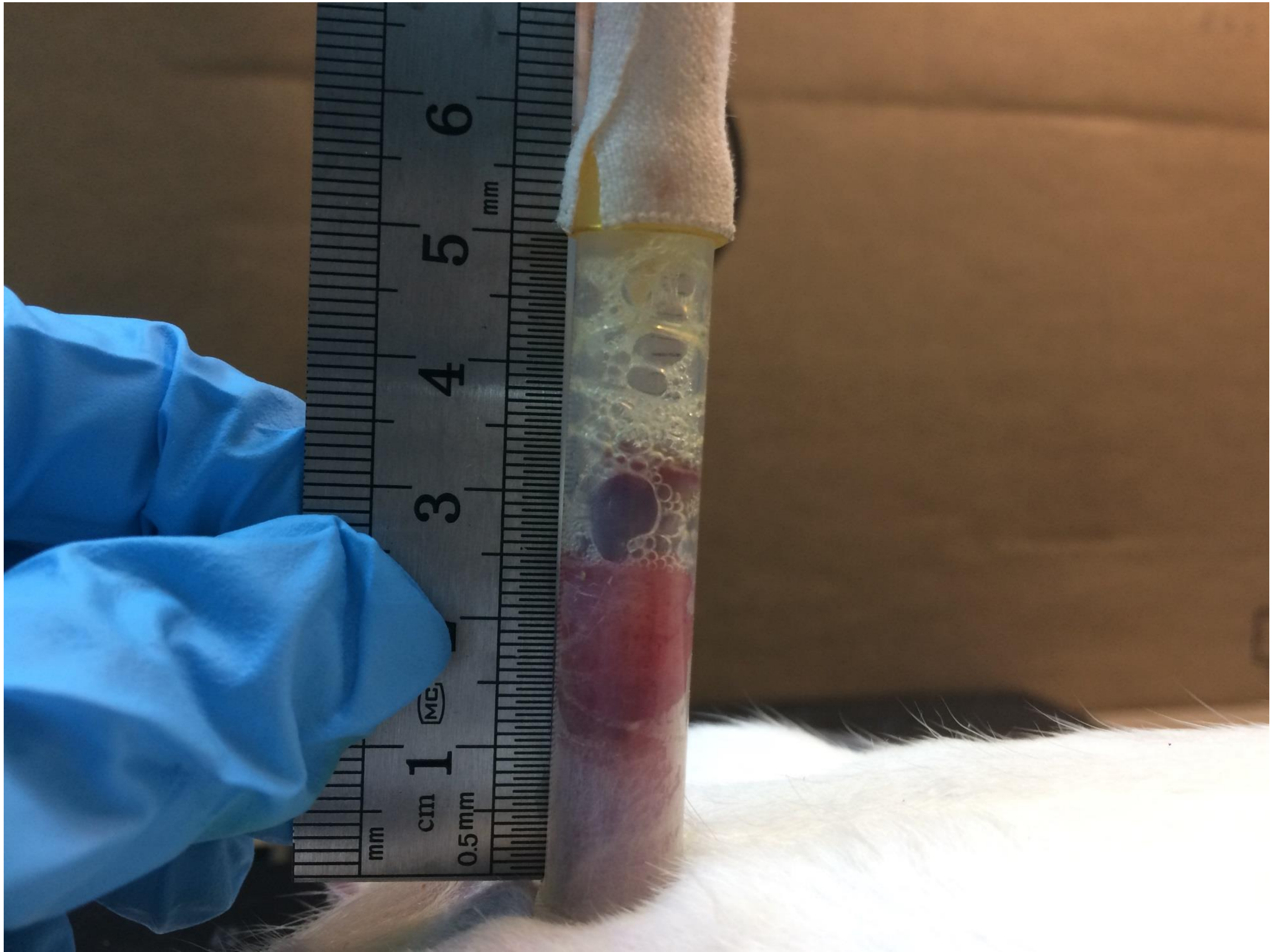

-300+HCG 5

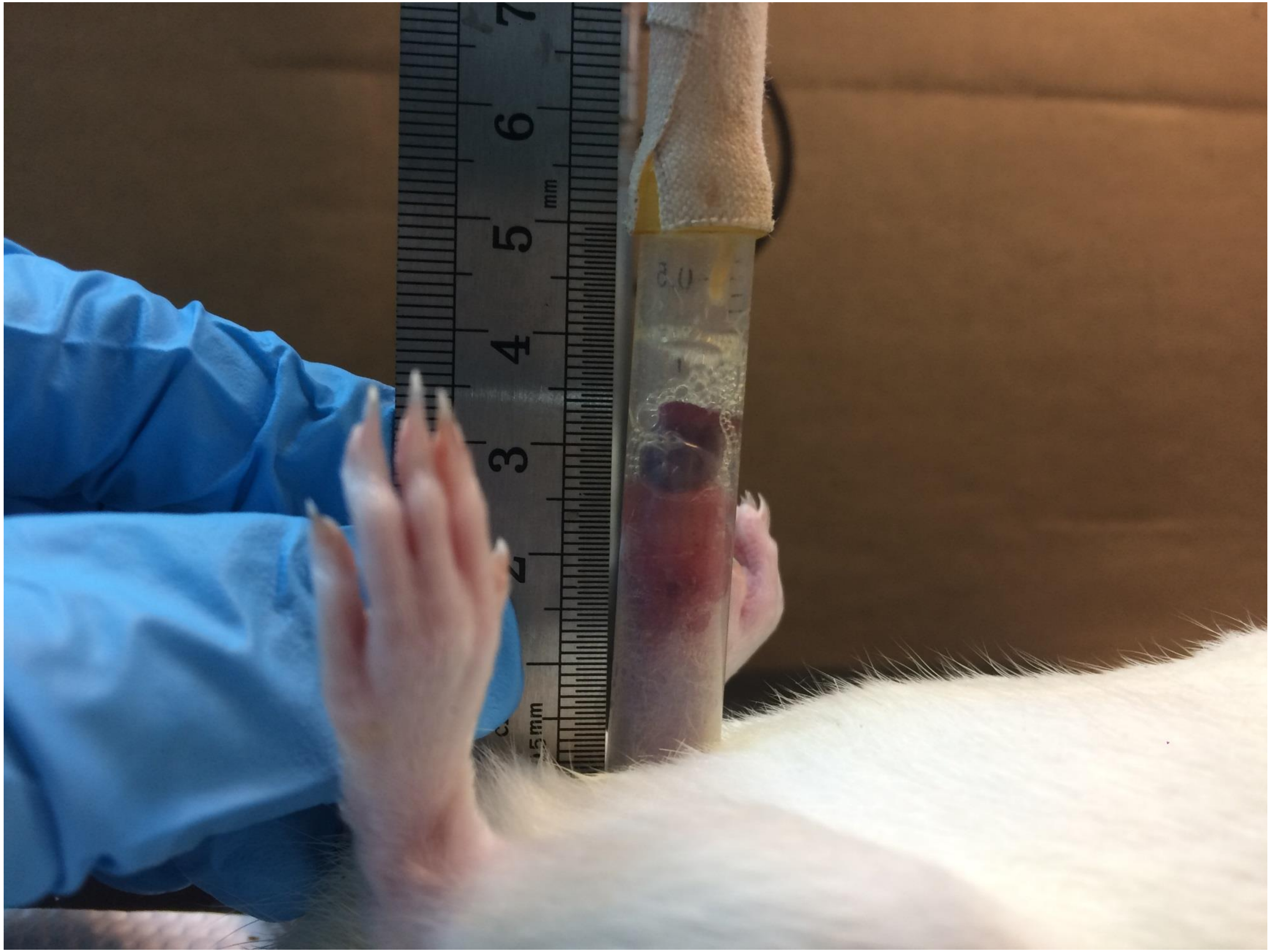

-300+HCG 6

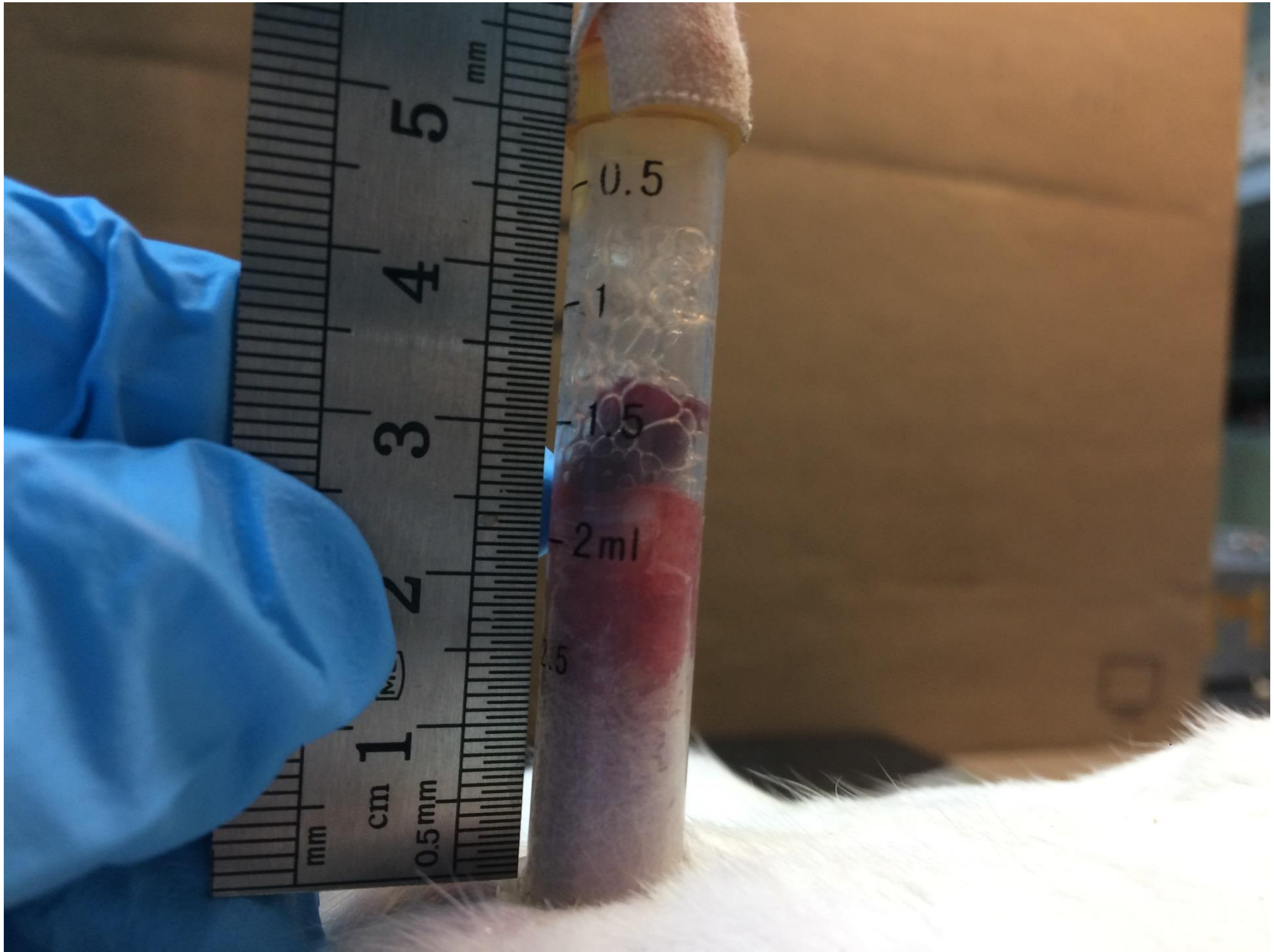

-300Anti-lox+HCG 1

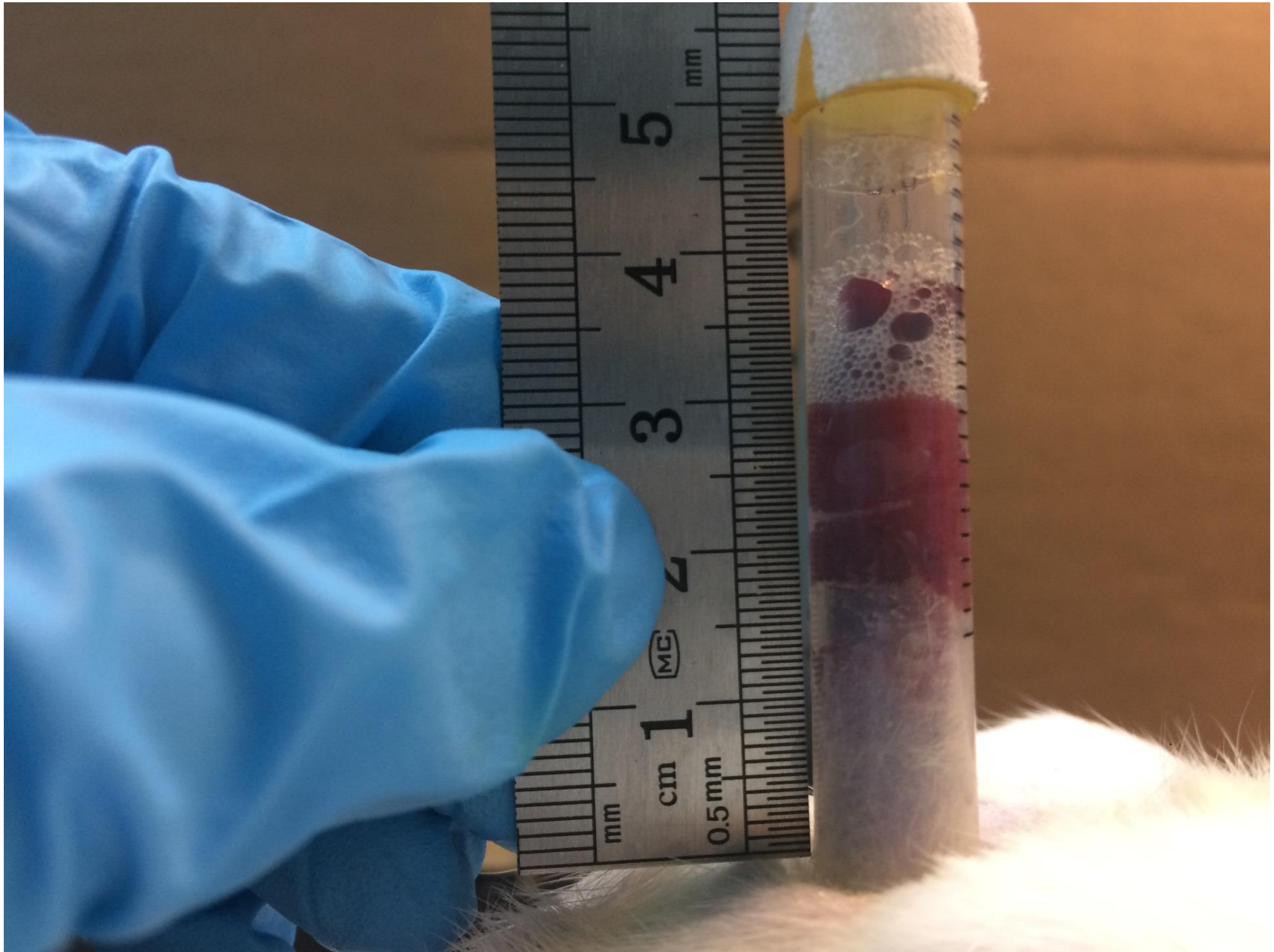

-300Anti-lox+HCG 2

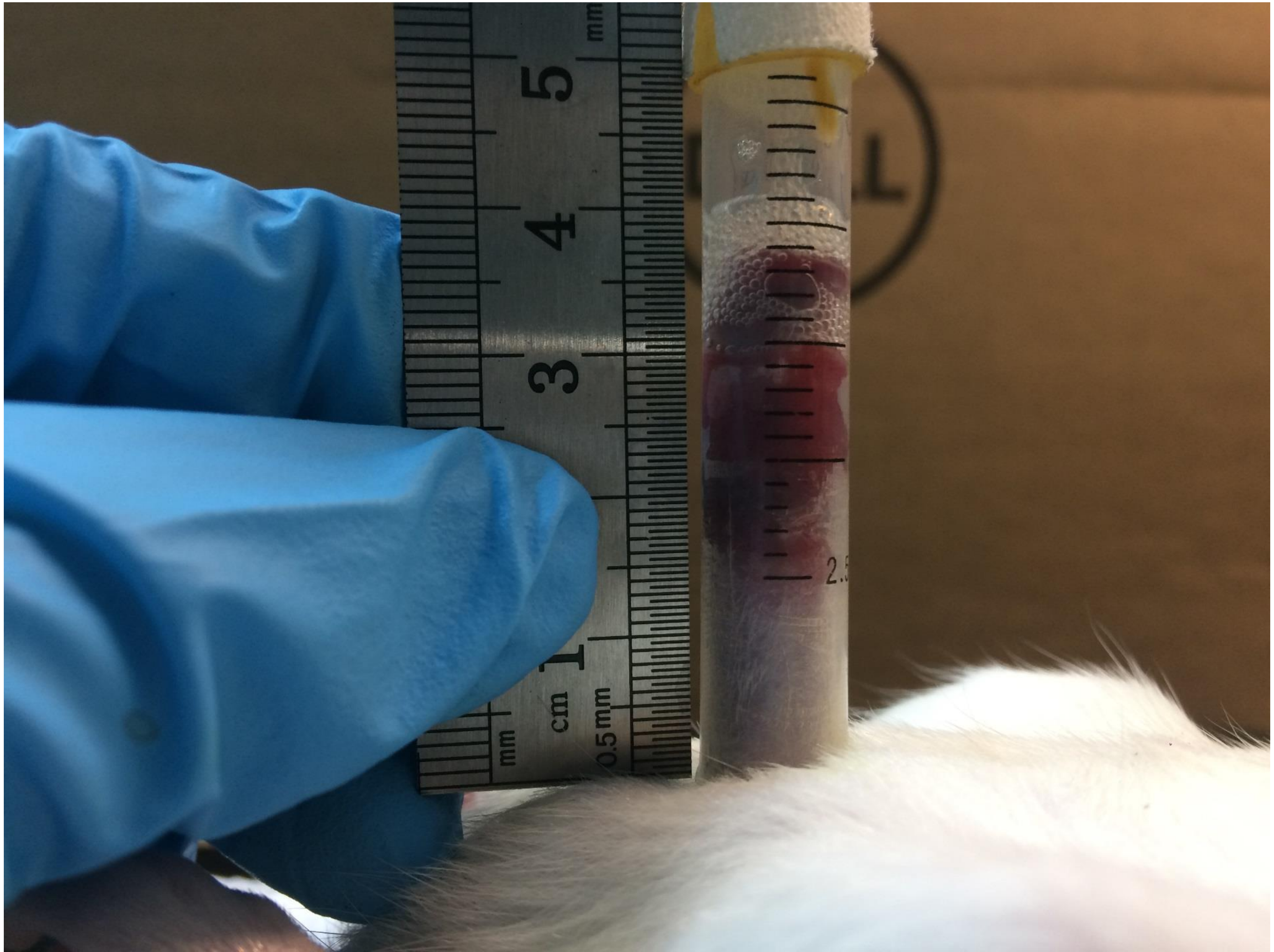

-300Anti-lox+HCG 3

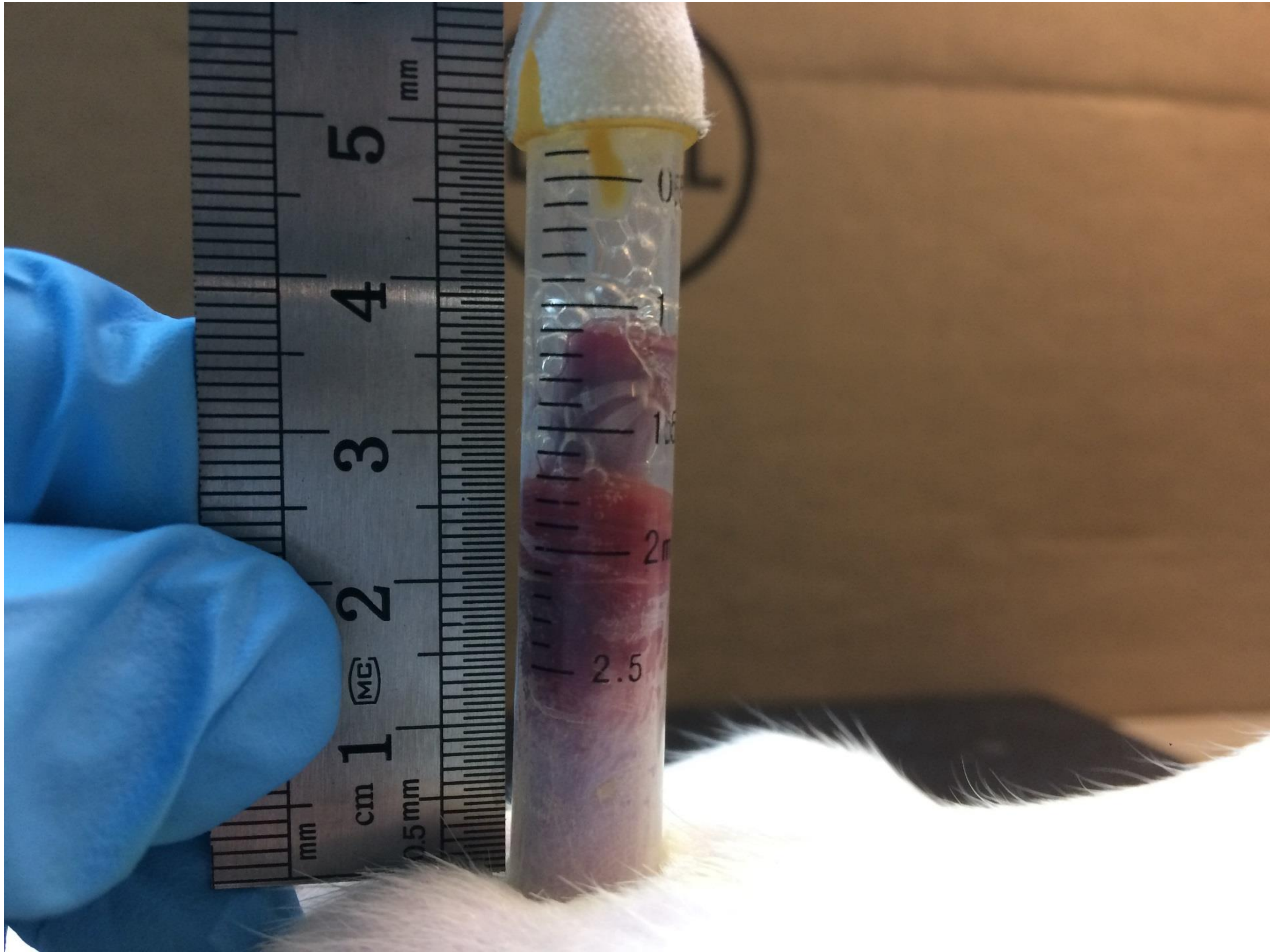

-300Anti-lox+HCG 4

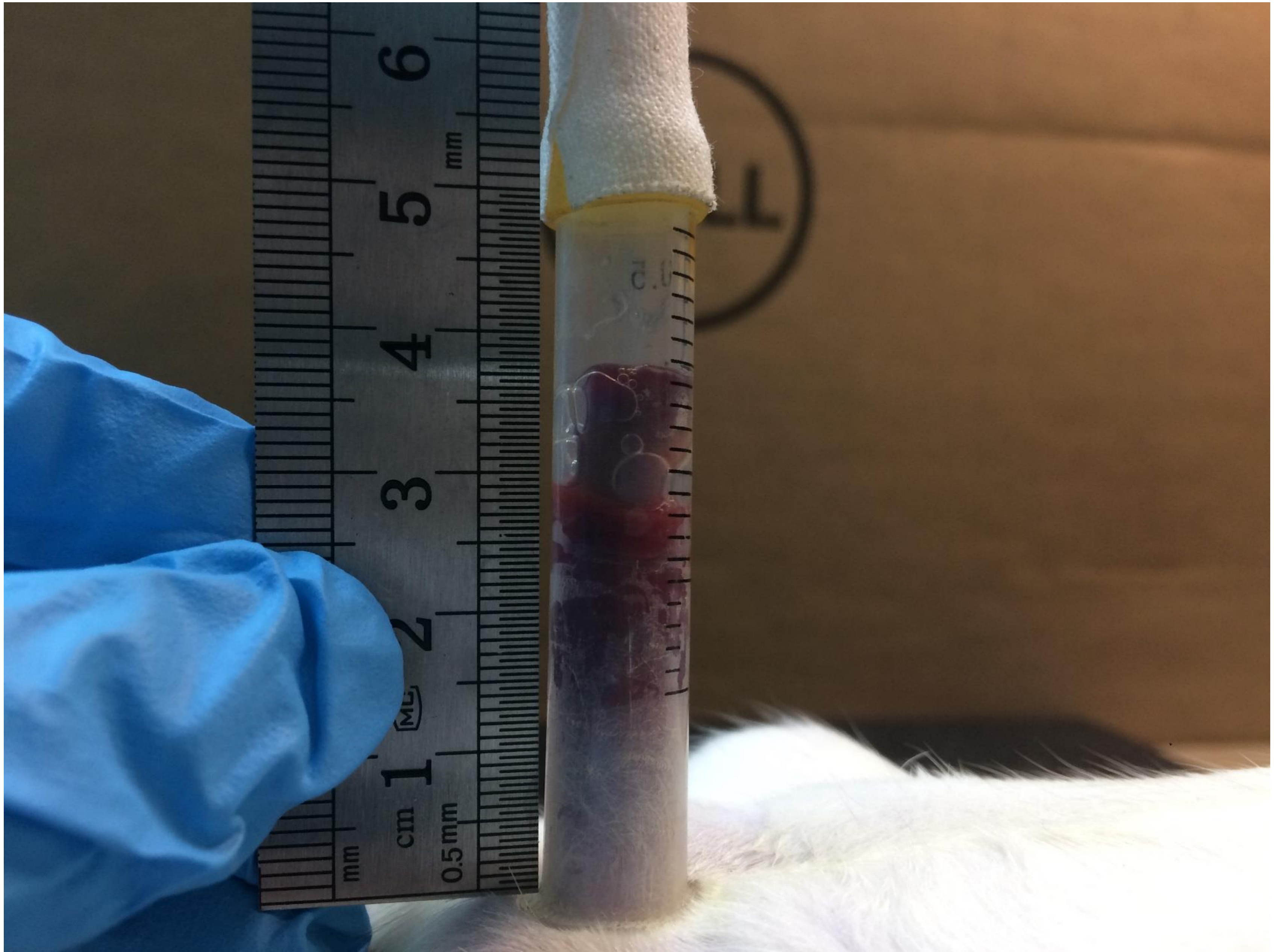

-300Anti-lox+HCG 6

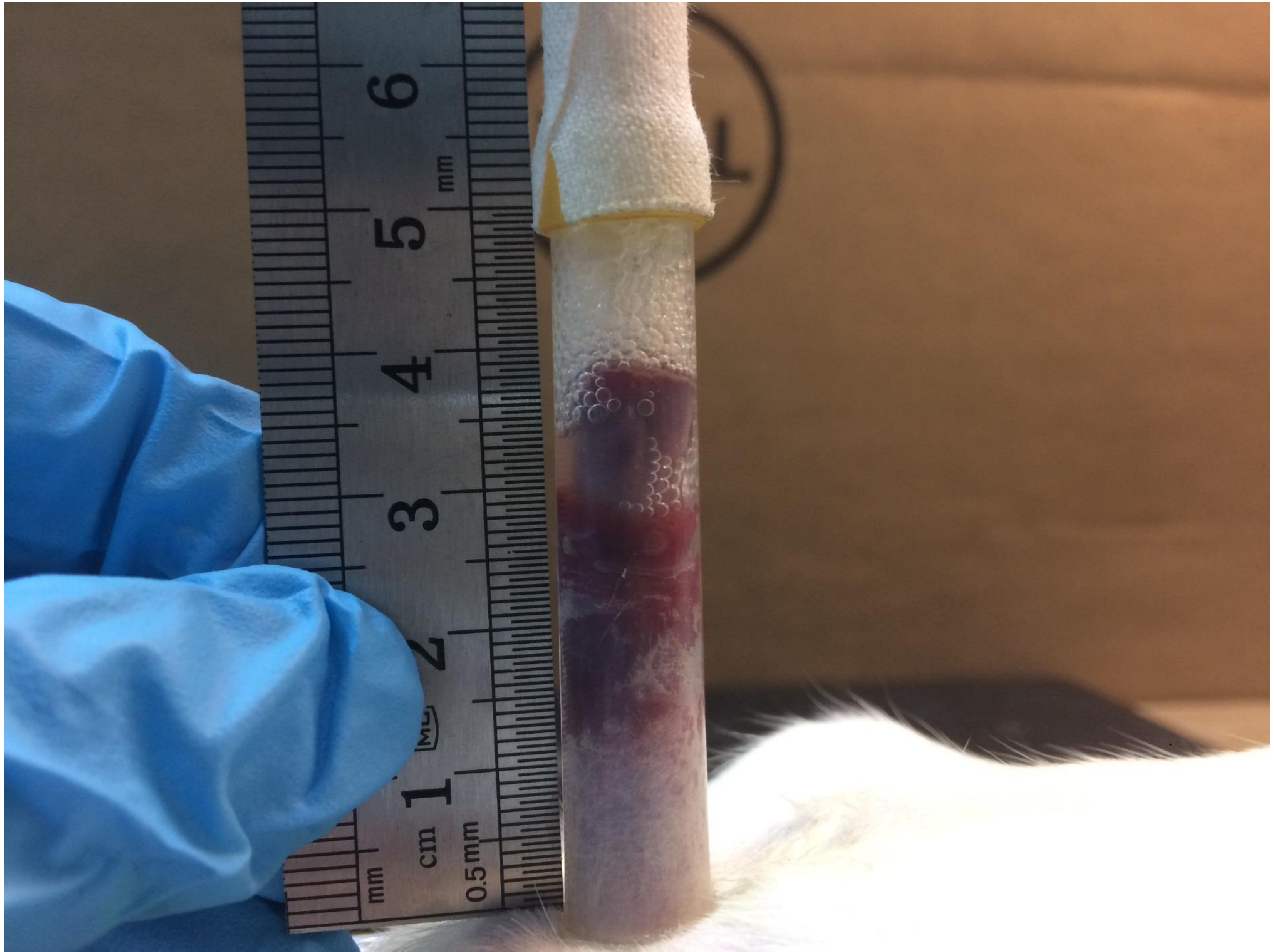

-300Anti-lox+HCG 6

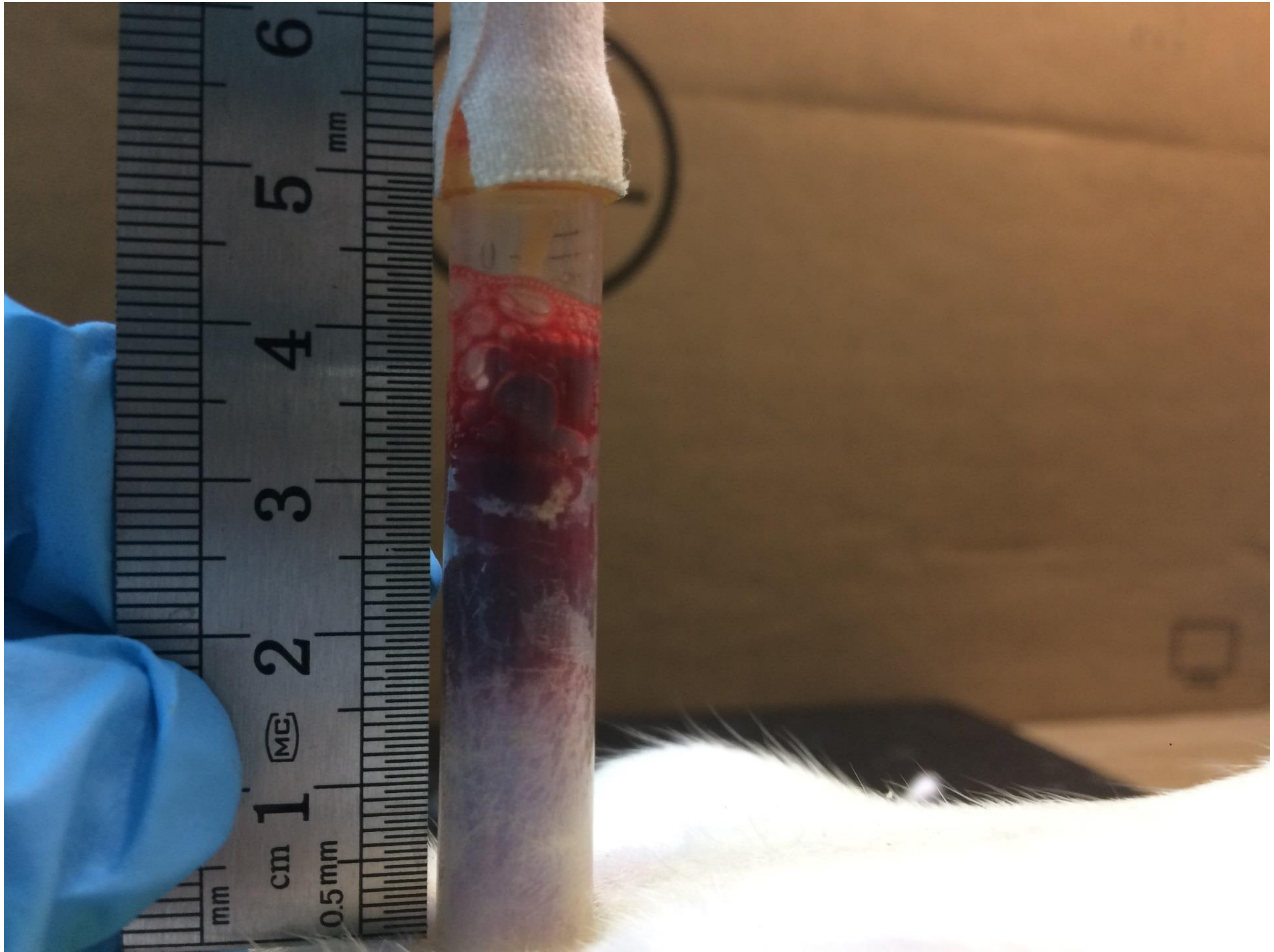

Supplement: Supplementary file 4 — Supplementary Information 4. [file 41598_2023_38888_MOESM4_ESM.pdf]
