# Supplementary figures and images for: HCG supplement did not accelerate tunica albuginea remodeling to facilitate penile growth
Source: Sci Rep. 2023 Oct 2;13:16519. doi: 10.1038/s41598-023-38888-y (PMC10545796; doi:10.1038/s41598-023-38888-y)

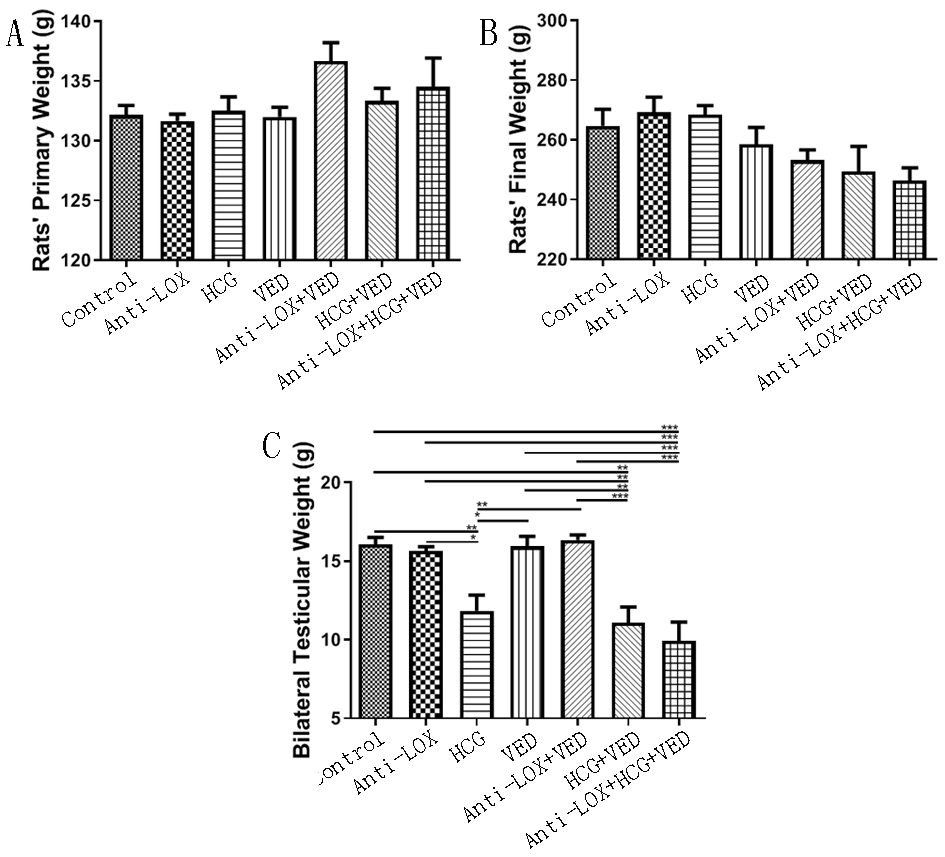

Supplement: Supplementary file 5 — Supplementary Figure S1. [file 41598_2023_38888_MOESM5_ESM.tif]
